# Relevance of ddRADseq method for species and population delimitation of closely related and widely distributed wolf spiders (Araneae, Lycosidae)

**DOI:** 10.1038/s41598-021-81788-2

**Published:** 2021-01-26

**Authors:** Vladislav Ivanov, Yuri Marusik, Julien Pétillon, Marko Mutanen

**Affiliations:** 1 Department of Ecology and Genetics, University of Oulu, Oulu, Finland; 2 Institute for Biological Problems of the North, RAS, Magadan, Russia; 3 Department of Zoology and Entomology, University of the Free State, Bloemfontein, 9300 South Africa; 4 UMR CNRS ECOBIO, Université de Rennes 1, Rennes, France

**Keywords:** Phylogenetics, Population genetics, Taxonomy, Zoology

## Abstract

Although species delimitation is often controversial, emerging DNA-based and classical morphology-based methods are rarely compared using large-scale samplings, even less in the case of widely distributed species that have distant, allopatric populations. In the current study, we examined species boundaries within two wolf spider species of the genus *Pardosa* (Araneae, Lycosidae), *P. riparia* and *P. palustris*. Wolf spiders constitute an excellent model for testing the relevance of traditional vs. modern methods in species and population delimitation because several closely related species are distributed over cross-continental geographic ranges. Allopatric populations of the two *Pardosa* species were sampled across Europe to Far East Russia (latitudinal range > 150°) and several dozen individuals were studied using morphological characters (morphometry of three measures for both sexes, plus five in males only and two in females only), DNA barcoding (COI sequencing) and double-digest restriction site associated DNA sequencing (ddRADseq). The results obtained allow for changing the taxonomic status of two Far East Russian populations to subspecies and ddRADseq proved to be a powerful tool for taxonomic research despite scarce sampling and inherent subjectivity of species delimitation in allopatry. Overall, this study pleads for both multi-criteria and more population-based studies in taxonomy.

## Introduction

Species delimitation is often challenging and controversial. Species is a central taxonomic category in various fields of biological research, yet the reality of species is still debated^1–3^. One of the reasons is the application of different operational criteria or species concepts results in various numbers of species under identical circumstances^4^. Combining different types of data in an integrative approach has the advantage of providing more informed decisions^5–8^. However, conflicts between results obtained through different species delimitation methods impose significant challenges for reaching uncontroversial conclusions^3,7^.

Traditionally, comparative morphology has served as the backbone in taxonomic routine^9^. Currently, broad datasets of DNA barcodes (portion of mitochondrial cytochrome *c* oxidase I in animals, COI) are often available along with morphological information. Studies implementing DNA barcodes and morphology on large geographic scales have shown correlations between COI divergence, morphological differences and geographical distance^10^. Operational species such as Barcode Index Numbers (BINs)^11,12^ provide a useful proxy for species boundaries. Yet, they do not always reflect them accurately as there are groups with high intraspecific or low interspecific variation^12–17^. The lumping of morphologically distinct spider species is reported from large German and Canadian datasets^12,18^. However, cases of shared mitochondrial DNA (mtDNA) between spider species have been known for a long time and are often explained by introgression or recent radiation^19–23^. As mtDNA can be identical between closely related species regardless of isolation or extent of geographical distance, further genetic data is required to elucidate true boundaries of species.

Reduced-representation genome methods such as restriction site associated DNA sequencing (RADseq), transcriptomes or genotyping-by-sequencing provide an unprecedented opportunity to “peek” into the genome^24^ and estimate divergence between populations, test for gene flow as well as compare closely related species. RADseq and similar methods have been successfully applied to species delimitation in different groups of organisms^25–30^ demonstrating a promising step toward the standardization of taxonomic routine. Despite the abundance of molecular data challenges have been reported^31,32^.

Geographical sampling plays a crucial role in species delimitation. Across a species range, morphological and/or genetic variations may be observed in different populations, thus further presenting challenges in determining the taxonomic status of a particular population^31–34^. Incomplete and biased sampling can easily lead to incorrect assignment of sampled populations. However, the reality of taxonomic routine is that there is often no possibility to obtain representatives from key locations although there is a demand to assign collected diversity to new or existing species^35^. DNA-based methods are prone to incorrect species inference in the absence of intensive sampling^36^, but they have the advantage of providing insights into historical relationships between target populations, estimating gene flow and placing examined specimens in a context of closely related taxa^34^. Biological processes, such as horizontal gene transfer, introgression and incomplete lineage sorting, further complicate species inference^37^.

The target taxa of the study are representatives of the family Lycosidae, wolf spiders. These spiders constitute an excellent model for testing the relevance of traditional versus modern methods in species and population delimitation as several closely related species are distributed over cross-continental geographic ranges^38^. Recent advances in spider molecular systematics^39,40^ suggest the family is relatively young with high to moderate diversification rates. Specialists in Lycosidae have taken advantage of molecular methods to study species boundaries and population genetics patterns in different genera but most studies were based on single or a few genes^22,41–53^. Species delimitation in Lycosidae is complicated by morphological homogeneity. However, courtship behavior^54–56^, ecological differentiation or geographical distribution^57^ may be a more reliable source of evidence for taxonomic decisions. A previous study^44^ suggested a clear association between morphospecies and genetic clades in sympatry when using double-digest restriction site associated DNA sequencing (ddRADseq) despite mitonuclear discordance.

In this study, the main interest was to evaluate the taxonomic status of allopatric populations in two separate cases of wolf spiders (Lycosidae) of genus *Pardosa* based on ddRADseq, DNA barcodes and morphology. Genus *Pardosa* is notorious for exhibiting shared mtDNA haplotypes in distinct morphological, ecological and behavioral species^12,18,44,58–62^ which complicates the use of DNA barcodes for specimen identification and species delimitation. The focus was on the following two wolf spider species, *Pardosa riparia* (C. L. Koch, 1833) and *P. palustris* (Linnaeus, 1758). Both species are represented in the current study by two distant populations from Finland and the Magadan region in Far East Russia (FER). The first species, *P. riparia*, belongs to the *pullata* species group and inhabits Europe, Turkey, Russia, Central Asia and Japan. The second, *P. palustris*, belongs to the *monticola* species group and has an even wider distribution, occurring in Eurasia and North America but not in Japan^63^. Initially, it was believed that the distribution of both species is uninterrupted as indicated by data in the World Spider Catalog. However, after an intense survey of the literature and consultation with colleagues in Russia, it is certain that populations of both species in the Magadan region are truly allopatric in relation to the rest of Russia and Europe (Fig. S1 and associated references, Supplementary material (SM) 1). Geographical barriers in this case are vast mountain ranges on the border of the Eurasian and North American tectonic plates (e.g., Verkhoyansky, Chersky, Kolymsky ranges). Spiders are capable of dispersal over thousands kilometers by ballooning^64–68^ but high mountains can impose a significant challenge for constant gene flow between populations. Thus, divergence between populations is likely to be driven by genetic drift as it is typical for the allopatric speciation process^69^. The initial hypothesis is that in both cases there are isolated populations of the same species which will be tested with molecular and morphological evidence. The current study suggests that ddRADseq could be superior or similar in its utility for species delimitation compared to classical morphological methods, especially in cases of genetically isolated populations where morphological evidence is of limited use.

## Results

### Morphological analysis

**Figure 1:**
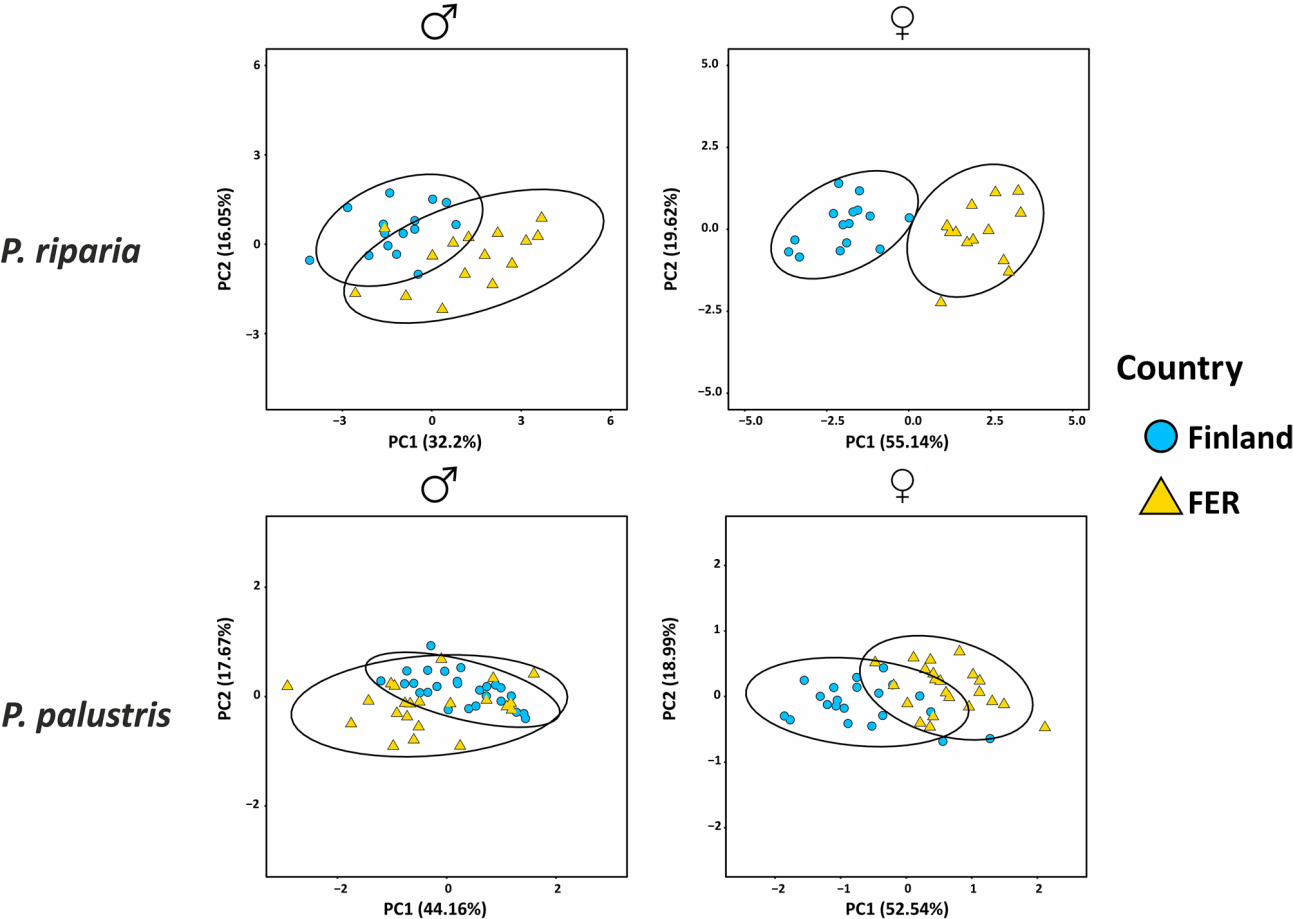
PCA of morphological measurements data for *P. riparia* and *P. palustris*. The proportion of the overall variance explained by each PC is shown in parentheses. Areas bordered by the line represent 95% confidence intervals.

No considerable qualitative differences in the structure of the copulatory organs were observed between the populations of *P. riparia* nor between the populations of *P. palustris*. Figures S2 and S3 in SM1 show the copulatory organs and habitus of the studied species. Some variations were detected, the first being the angle of the entrance duct openings relative to the septum in female *P. riparia* suggesting prezygotic isolation due to copulatory organs structural incompatibility (Fig. S2, SM1). The second variation was the terminal apophysis of *P. palustris* where in Finnish males it had long and sharp teeth on the distal margin, while in the Russian population it was smoother (Fig. S3, SM1). The PCA did not suggest differences between populations based on the measurements of morphological characters, except in the case of *P. riparia* females (Fig. 1). Boxplots (Fig. S5 and S6, SM1) indicated considerable overlap in copulatory organ measurements, while somatic characters showed greater divergence. Leg length (PatTib) showed the largest differences between populations in both species but failed the Bartlett test for homoscedasticity and could not be utilized for further analysis. The ANCOVA analysis suggested significant differences in the variances of male copulatory organ lengths in both species when taking into account body size. In females the size differences in copulatory organs were mostly insignificant. Additional t-test with unequal variances mostly corresponded with the ANCOVA (Table S6).

### ddRADseq assembly and datasets

Many datasets with various parsing were assembled to examine the effect of the amount of missing data and to investigate how clustering at different values of *m* affected the *p*-distance results (Table 1). The amount of missing data ranged from 15.6 to 82.3%. Percentage of SNPs was between 4.3 and 9.6 for different datasets. Rip_pal datasets included all sequenced specimens with outgroups, Pal_mon datasets (except _m10c85) included available specimens from the *monticola* group, Rip datasets included only the *pullata* group while the Pal_mon_m10c85 dataset contained only *P. palustris* and *P. ovtchinnikovi.* The datasets Rip_m10c85 and Pal_mon_m10c85 were used for STRUCTURE analysis to measure the extent of gene flow between populations and between closely related species. High *m* values were used in these analyses to diminish the proportion of missing data. A value of 85 was found to be optimal for the clustering threshold and was used for all datasets. Summary statistics for the assemblies are provided in SM2.

**Table 1:** Summary of the ddRADseq datasets used in the downstream analysis.

Matrix	*n*	Number of loci	Number of unlinked SNPs	Consensus sequences (bp)	VAR (%)	PIS (%)	Missing data (%)
Rip_pal_m3c85	36	64,629	57,072	11,844,992	5.4	1.5	82.3
Rip_pal_m6c85	36	23,616	22,974	4,350,581	7.4	2.9	75
Rip_pal_m9c85	36	8950	8906	1,660,667	8.8	3.7	68.3
Rip_pal_m12c85	36	3146	3129	588,319	9.4	4.1	59.5
Rip_pal_m15c85	36	1262	1256	236,744	9.6	4.3	51.2
Rip_pal_m18c85	36	568	567	106,350	9.6	4.1	44.7
Rip_pal_m22c85	36	124	124	23,168	9.0	3.9	35.8
Rip_Pal_IN_m6c85	32	23,372	22,675	4,306,371	6.8	2.9	72.8
Rip_m10c85	18	684	670	127,926	6.6	2.6	40.6
Pal_mon_m10c85	12	2123	2071	395,237	5.4	2.7	15.6
Rip_m6c85	18	5431	5340	1,016,811	6.4	2.4	58.4
Rip_m4c85	18	11,914	11,609	2,232,854	5.7	1.8	67.9
Pal_mon_m4c85	14	39,831	36,284	7,266,287	4.8	1.8	52.9
Pal_mon_m6c85	14	22,115	21,213	4,058,676	5.5	2.3	43.9
Pal_mon_m3c85	14	54,495	46,703	9,929,008	4.3	1.3	57.7

n: number of individuals; VAR: percentage of variable sites; PIS: percentage of parsimony informative sites. Dataset names are organized as following: Rip_pal—*pullata* and *monticola* groups combined plus outgroup species; Rip_pal_IN—same as previous but no outgroup; Rip—*pullata* group only; Pal_mon—*monticola* group only; m—minimum taxon coverage; c—clustering threshold.

### Phylogenetic inference, haplotype networks and species trees

Maximum likelihood trees for COI that included only specimens attempted for ddRADseq confirmed the previously reported DNA barcode sharing within the *pullata* group (Fig. S7, SM1). The same was observed in the ML COI tree that included all public records in BOLD (SM3). In the case of *P. palustris*, the pattern was similar, populations from Russia were clustered separately to the Finnish and Faroe Island specimens for the ddRADseq mimicking dataset (Fig. S7, SM1). The same was observed in the ML tree built in IQ-Tree and species ID tree built in BOLD for the *monticola* group (SM3 and SM4). However, when building a species ID tree in BOLD for the larger dataset of the *monticola* group, FER and American specimens fell within one of the European clusters (SM5). When the same was done for the *pullata* group, *P. riparia* becomes a sister group to all other species in the *pullata* group (SM4).

**Figure 2:**
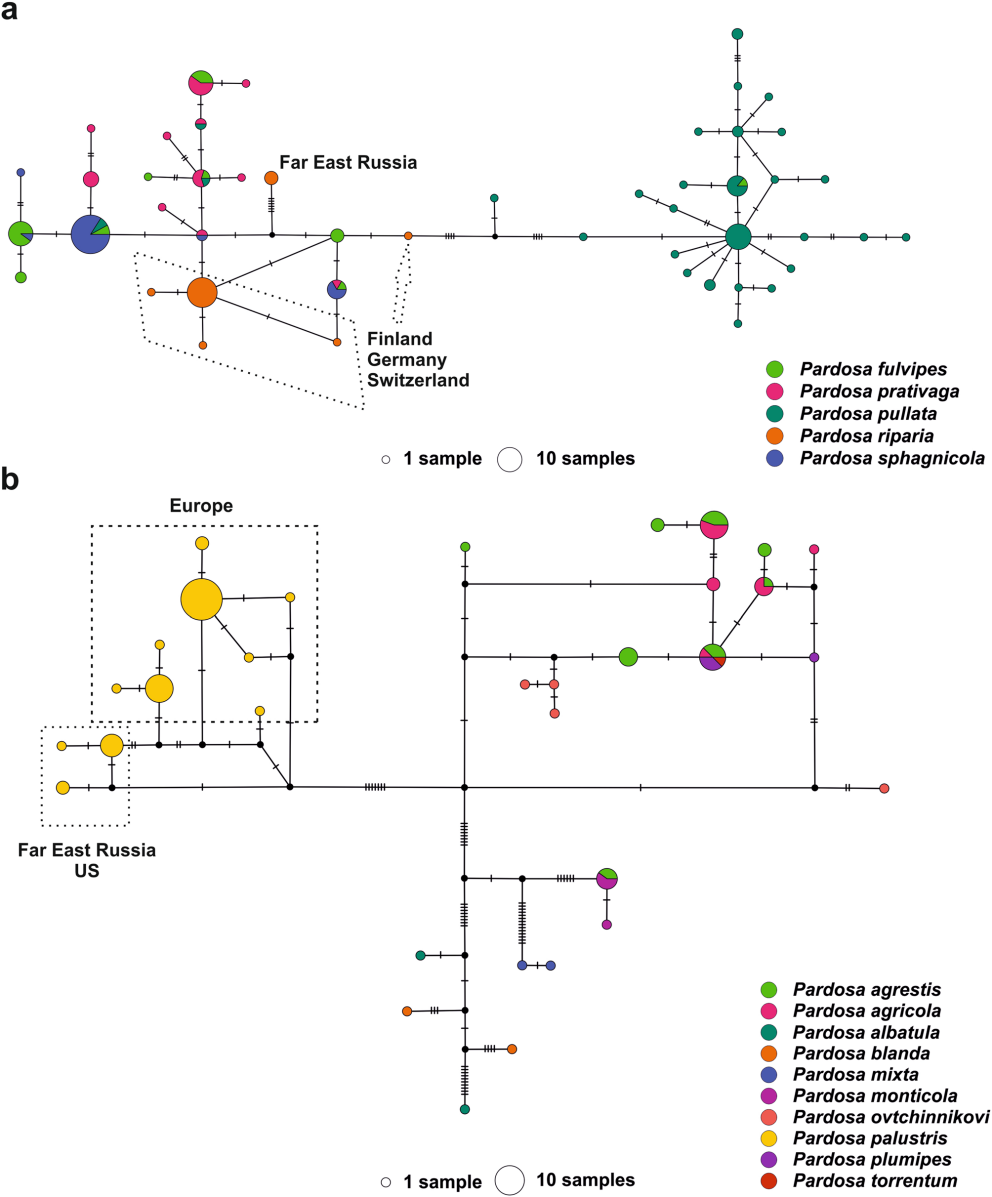
COI haplotype networks. (**a**) *pullata* species group, (**b**) *monticola* species group. Each circle represents a haplotype and circle size is proportional to strain frequency. Different colors represent different species. Lines between haplotypes and short solid lines are single mutational steps. Dotted lines outline haplotypes by populations. Black dots are predicted or missing haplotypes.

The haplotype network built for the *pullata* group (Fig. 2a) indicated that all sequenced *P. riparia* from FER belong to the same haplotype, while Finnish specimens had several haplotypes mixed with other representatives of the species group. In the case of *P. palustris* (Fig. 2b)*,* its position was similar to *P. pullata* in its own species group, i.e. it did not share haplotypes with other representatives of the *monticola* group. Specimens from FER and America nevertheless could be separated from the *P. palustris* haplotypes from other countries, thus indicating divergence in COI.

**Figure 3:**
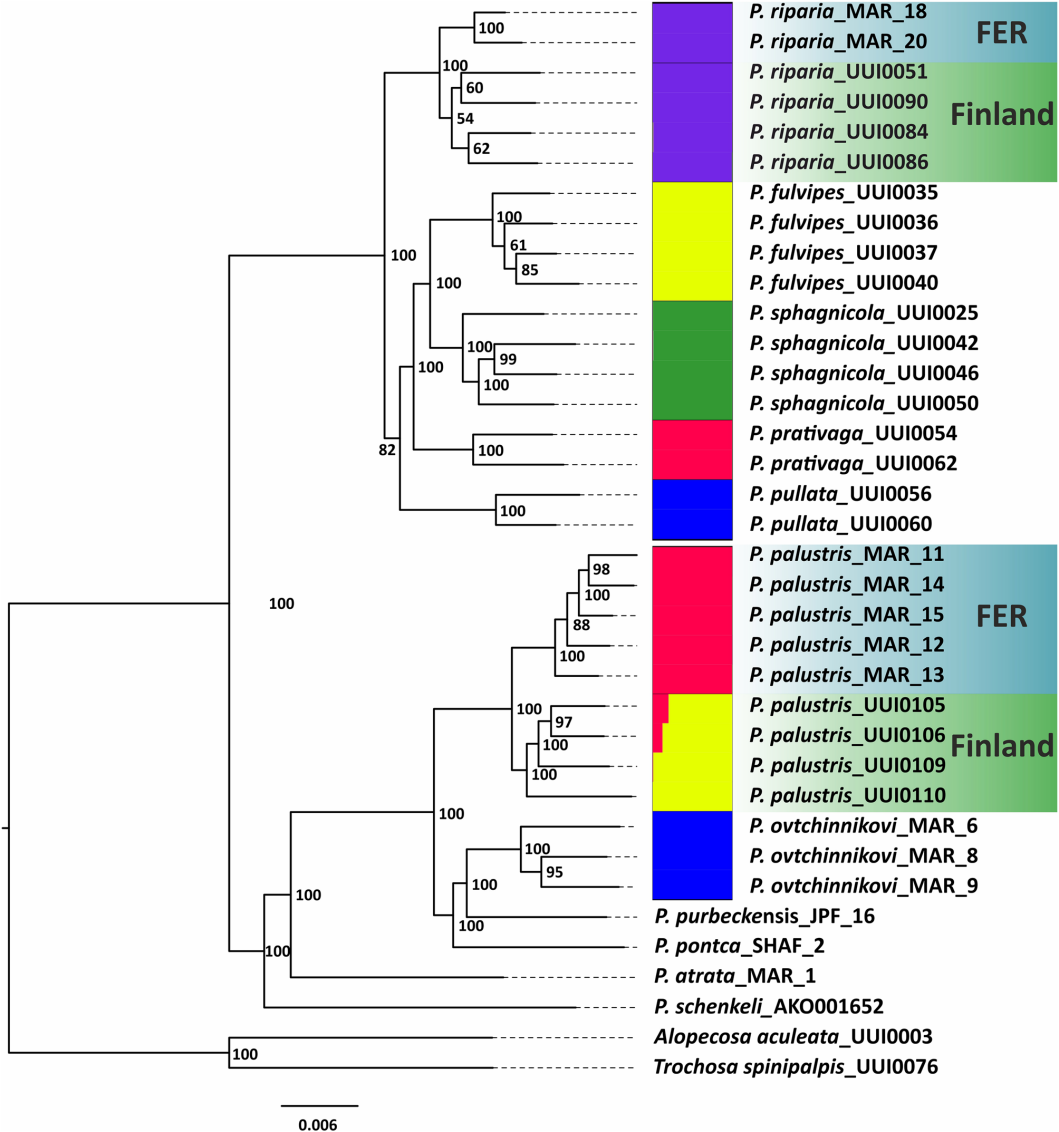
Maximum likelihood tree (Rip_pal_m6c85 dataset) and STRUCTURE barplots (Rip_m10c85 and Pal_mon_m10c85 datasets) based on ddRADseq data. Numbers indicate bootstrap support values for nodes. STRUCTURE barplots represent the patition with highest K supported where K = 5 for *pullata* group and K = 3 for *monticola* group.

The ML tree based on ddRADseq data distinguished between Russian and Finnish samples for both species but also recognized morphospecies of *pullata* and *monticola* groups as separate lineages (Fig. 3). Species trees calculated using SVDquartets implemented in PAUP* showed high support for splitting distant populations into two different species. However, the bootstrap support in inner nodes remained quite low (50), thus SVDquartets did not support *P. pullata* as a distinctly separate species. The same was observed within the *monticola* group, where the split between *P. palustris* and the rest of the species had a bootstrap value of 50 (Fig. S8, SM1).

### Species delimitation

BIN analysis conducted in BOLD in both cases assigned Russian specimens to already known European species (*P. riparia* BIN: BOLD:AAF7515; *P. palustris* BIN: BOLD:ACH2627). Species discovery methods GMYC and bPTP returned contradictory results based on COI analysis and were largely dependent on the dataset analyzed. GMYC oversplit both species when analyzed alone or in combination with species from the same species groups. When the two species groups were combined, the FER populations were lumped into one species in both cases. Results of bPTP were similar in that the number of suggested species decreases when more distant lineages were added but they were largely oversplit. Summary of the GMYC and bPTP analyses are in Tables S7 and S8 (SM1), figures of the trees are available upon request.

The Bayes factor calculated on the SNAPP results favors the two species hypothesis in both cases (381.01 for *P. riparia* and 295.22 for *P. palustris*).

### Testing for admixture

The STRUCTURE results suggested that *P. riparia* from Finnish and Russian populations belongs to the same gene pool in relation to other species of the *pullata* group. The highest probability was observed at K5 (Fig. S9, SM1). Population genetic analysis suggested that representatives of *P. riparia* from FER and Finland belong to the same species (Fig. 3). The situation with *P. palustris* is different. STRUCTURE results suggested that the Russian and Finnish populations had limited shared ancestry (Fig. 3). The mean estimated probability was higher for K3 than for K2 (Fig. S9, SM1), thus *P. palustris* populations appeared more diverged than *P. riparia* populations.

### Pairwise distance patterns in ddRADseq datasets

**Figure 4:**
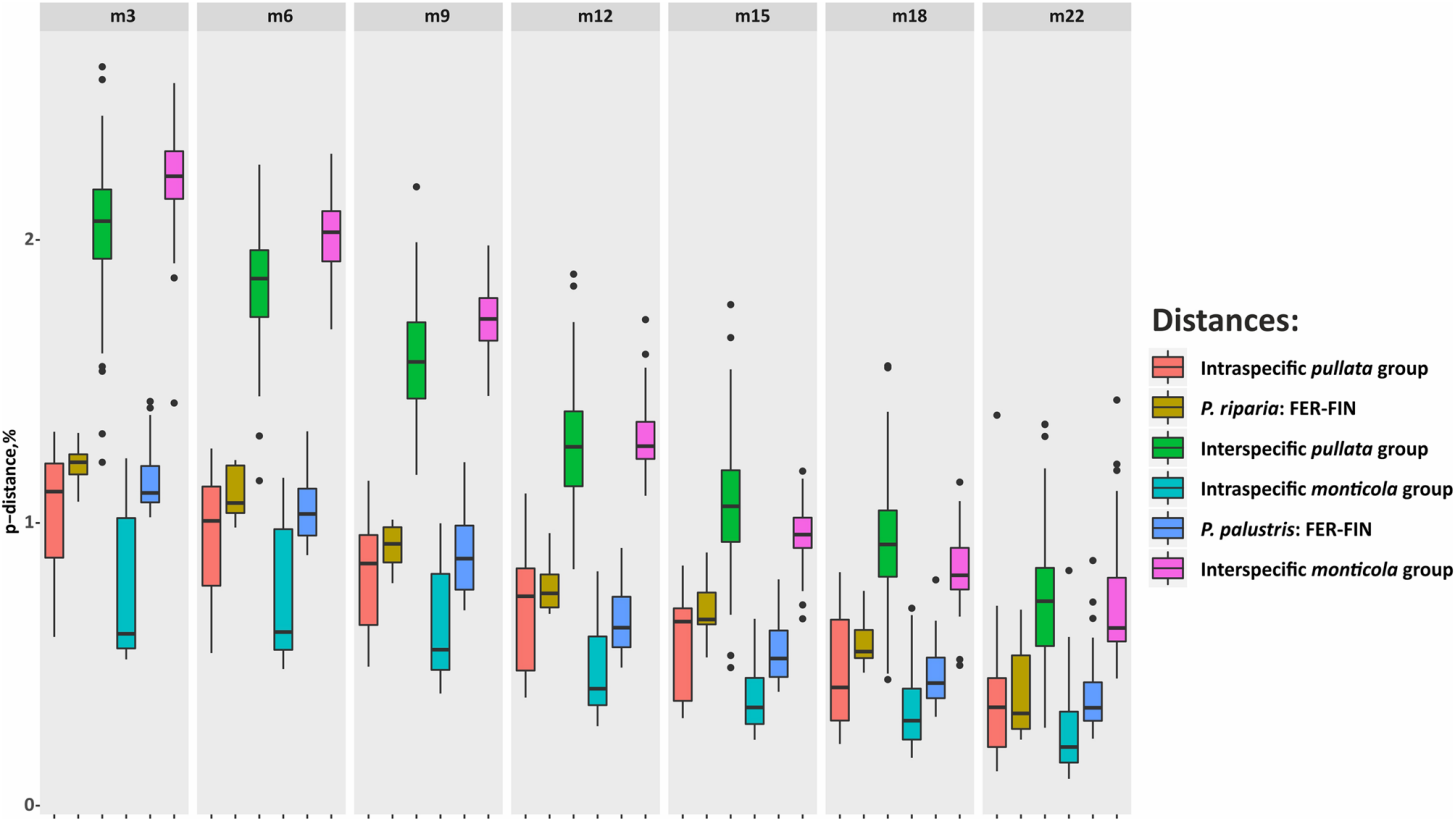
Boxplots of absolute values of *p*-distance. Each section represents Rip_pal_ datasets assembled at a particular *m* value indicated at the top of the section. The *p*-distances for *pullata* and *monticola* species groups and between focus populations of *P. riparia* and *P. palustris* are calculated separately. Boxplots show median, quartiles and 95% confidence intervals.

Intra- and interspecific *p*-distances varied dramatically depending on *m* value. The clear difference between intra- and interspecific distances was observed from *m* = 3 to *m* = 9 in the *pullata* group and from *m* = 3 to *m* = 15 in the *monticola* group (Fig. 4). Concurrently, *p*-distances between the FER and Finnish populations for both species remained within the 95% confidence interval of intraspecific distance irrespective of *m.* In addition, means of *p-*distances between *P. palustris* populations remained non-overlapping while *p*-distances between *P. riparia* populations were closer.

**Figure 5:**
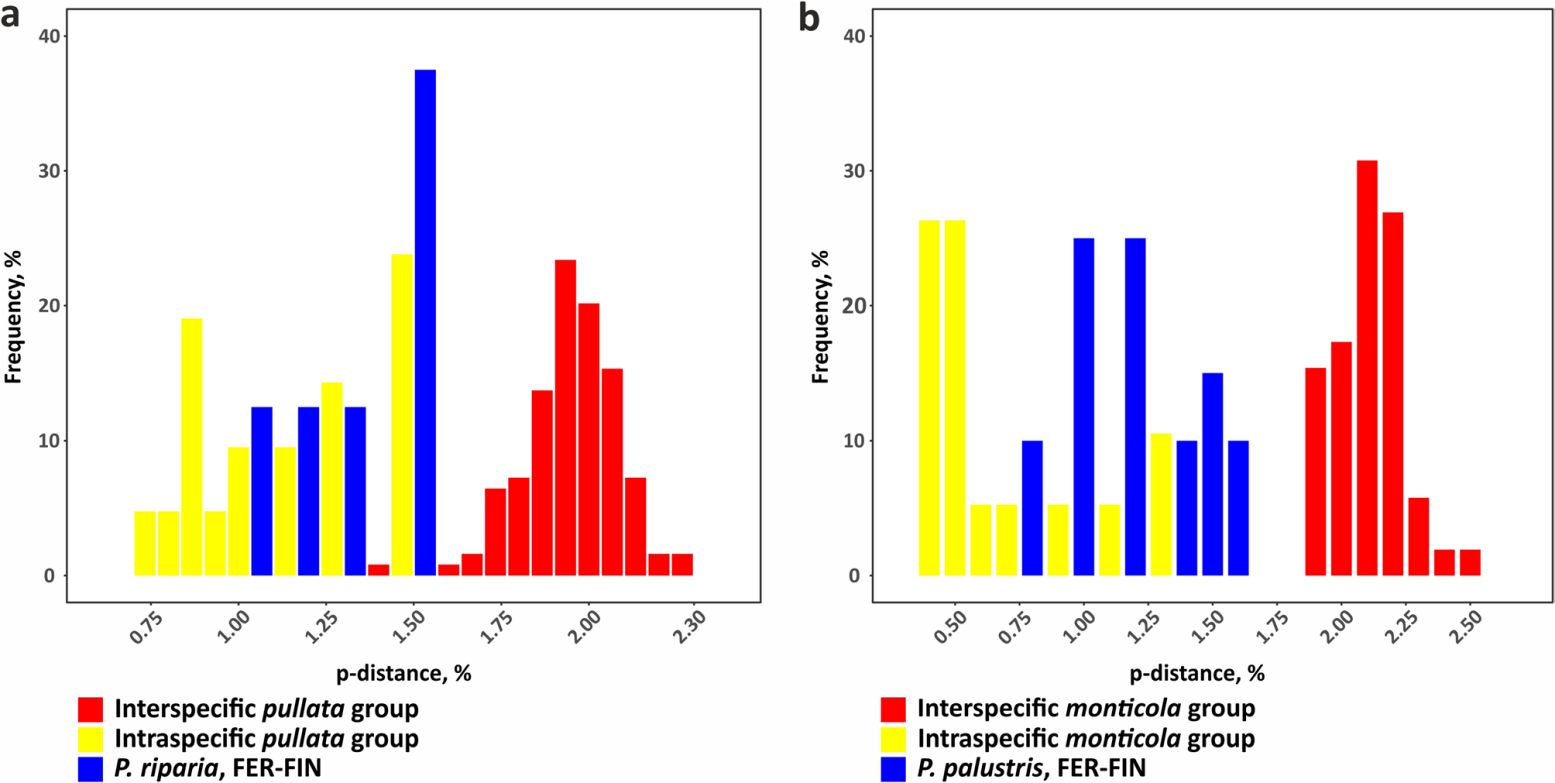
Frequencies of *p*-distance values observed within species, between species and between distant populations of target species at *m* = 6. (**a**) *pullata* group and *P. riparia* specimens, Rip_m6c85 dataset; (**b**) *monticola* group and *P. palustris* specimens, Pal_mon_m6c85 dataset.

The *p*-distances were visualized similarly to the barcode gap, i.e., as plotted frequencies of *p*-distances for interspecific, target populations and intraspecific values. There was evident gap between intra- and interspecific distances and target populations are likely to belong to intraspecific values distribution at *m* = 6 (Fig. 5).

## Discussion

While species delimitation in spiders using molecular methods has a long tradition (e.g.^70^), there are only a few studies that incorporate both morphology and genetics in Lycosidae and even less so using multiple genes (e.g.^41,43,56,59,71^). Therefore, this study is one of the first combining ddRADseq, DNA barcodes and morphology to study the problem of allopatric species in wolf spiders and, to our best knowledge, the first to compare FER populations of target species to European populations. While results of different analyses are not entirely conclusive, similar trends are observed for both *P. riparia* and *P. palustris*. It is acknowledged that limited sampling imposes difficulties in drawing completely unbiased conclusions. However, we suggest that the abundance of data collected should elucidate the questions asked. The congruence of the results are discussed below, their biological meaning, taxonomic decisions that can be made based on the presented evidence and implications of this research for further studies.

All types of data (morphological, COI and ddRADseq) clearly show divergence between distant populations. It is to be expected for the case of true allopatric populations where interaction between populations is prohibited by a natural barrier. Therefore, one line of evidence that supports the two-species hypothesis for both cases is geographical isolation that lasted long enough to promote detectable divergence in morphological characters and DNA. Distribution maps provided (Fig. S1, SM1) are the most up to date for both species.

The traditional morphological approach that was followed in this study was aiming to find characters that would be indicative of divergence to the extent of prezygotic reproductive isolation and/or considerable divergence between populations. In entomology and arachnology, genital differences have played a particular role in often showing rapid divergent evolution^72^. Differences in genital structures have been thought to form a mechanical reproductive barrier between species through character displacement (the lock-and-key hypothesis), but genital evolution is more likely driven by sexual selection^73^. Qualitative assessment of morphological characters indeed shows several indicative features that could be used for reliable identification including the copulatory organs. Measurements suggest that length of the first leg of *P. riparia* is a simple way to tell two populations from each other which should be considered given importance of the first pair of legs for *Pardosa* reproduction (e.g.^74^). However, in *P. palustris* populations there is no such striking difference in the measurements. Moreover, the failed Bartlett test suggests that additional measurements are needed or, alternatively, leg length might be misleading and should be avoided as a diagnostic character for identification and delimitation at this stage. Further intensive sampling is required to confirm if this character could be used as a diagnostic feature. PCA based on the same measurements suggests greater divergence in *P. riparia* than in *P. palustris*. Variation reported here coupled with the geographical distribution could be enough to erect FER populations to at least subspecies status. However, we believe that morphology alone is insufficient in this case as subjectivity of character choice is an inherent problem for traditional taxonomic studies. DNA sequences were thus examined for congruence with the morphological results, as the DNA sequence analyses rely on more general evolutionary theories and follow more standardized procedures for acquiring and analyzing data.

DNA barcoding again proved to be limited source of evidence for species delimitation in *Pardosa*. Nevertheless, both FER populations have diverged haplotypes from European ones, especially in *P. riparia*. The eastern population of *P. palustris* is clearly closer to Alaskan populations than to European (one mutation difference against five) while *P. riparia* populations form separate clusters in both ML trees and haplotype networks. Species delimitation methods such as GMYC and bPTP are heavily criticized for oversplitting species in spiders^33,75^ and despite the results being reported here we refrained including them in the final taxonomic conclusion. Briefly, in the context of closely related species both populations in both species are split into three to 41 species by GMYC or bPTP. If two species groups are combined, then the *P. palustris* populations appear as one homogenous cluster irrespective of geography. While *P. riparia* populations are lumped with other species of the *pullata* group much like the species ID trees from BOLD and the COI ML trees. Overall, COI remains inconclusive for *Pardosa* species delimitation, but slight differences observed corresponded to the distribution data, i.e., isolated FER populations have different haplotypes from European populations, and it can be used as proxy for quick identification using DNA barcodes.

Genomic data provided evidence that FER populations in both species belong to a separate cluster based on ML trees and the species delimitation results from SNAPP for both populations in both species. It should be noted here that multispecies coalescent methods (MSC) have been criticized for delimiting structure rather than species^76^, while it is suggested that they are better justified from an evolutionary point of view^77^. Therefore, given the limited number of scenarios that could be tested with SNAPP and the lack of agreement about the utility of MSC for species delimitation, the supported two species hypotheses should be treated as a reflection of divergence between distant populations rather than decisive evidence for species boundaries. The species tree computed in PAUP* has high support for the split but low support for separation of the target species from their respective species groups. Thus confirming the presence of genetic differences between populations but telling little about actual species statuses. Simultaneously, *p*-distances and population genetic analysis suggest that distant populations are a single entity rather than two diverged species when compared to closely related species. Only in the case of STRUCTURE results for *P. palustris,* distant populations are forming two clusters with limited shared ancestry. However, this could be an artefact of insufficient sampling. Overall, ddRADseq data suggest detectable divergence between European and FER populations but do not unequivocally support the presence of separate species in the Magadan region.

The taxonomic decision about FER populations is straightforward given a certain level of congruence between DNA and morphological data. All lines of evidence despite some discrepancies suggest a level of divergence of FER populations of *P. riparia* and *P. palustris* detectable by morphological examination and DNA sequencing. However, not to the extent to confidently claim species level divergence if using closely related species as a yardstick. Therefore, subspecies status for eastern populations of *P. riparia* and *P. palustris* is the most appropriate and compliant to the data analyzed. The main point of distinguishing differences between populations in scientific name is that it shows detected diversity in a quick and straightforward way, easily accessible for various specialists thus enabling proper faunistic, taxonomic and evolutionary studies planning and provoking further research. There seems to be a tradition of avoiding subspecies status in arachnology when no clear morphospecies can be described^78^, thus we do not suggest names at this stage to avoid inflation. Nevertheless, this current research implies that described cases of *P. riparia* and *P. palustris* could serve as model populations for studying recent divergence and speciation as such. However, a more favorable outcome would be a clear taxonomic decision that we are planning to present when additional data is collected. A somewhat similar view is stated in a considerably more data rich study of the *Habronattus tarsalis* species complex (Salticidae). There, multiple isolated populations exhibited deep genetic and morphological differences but the authors were reluctant to discuss their taxonomic status as the process of divergence is believed to be more important for future studies and naming species is “somewhat missing the point”^79^. While for evolutionary and population genetics studies such a view can be justified, it does not add to the documentation of biodiversity which is one of the major unfulfilled tasks for humanity. As genome scale sequencing is becoming more and more accessible it can strongly facilitate species discovery and validation, we would encourage researchers to be more bold in using DNA evidence alone for taxonomic decisions especially if genome level sequencing is available.

To conclude, ddRADseq is a powerful tool for research in taxonomy as it can supplement traditional morphological methods with historical perspective and provide data on current genetic processes. Therefore, genomic tools can give insight into the old problem of species delimitation in allopatry by adding additional layers of objectivity despite inherently arbitrary final decisions. However, at the current stage of the research we would like to restrain from giving formal description of subspecies before the major populations across the whole area of distribution are studied even though the amount of evidence is more than sufficient for such a conclusion.

## Materials and methods

### Sampling

Specimens for morphological (155 specimens), COI (25 specimens) and ddRADseq (17 specimens) analyses were sampled from target populations of *P. riparia* and *P. palustris* in Finland and the Magadan Region, FER. These were coupled with representatives of *pullata* and *monticola* species groups from Europe and Asia, several other *Pardosa* species and outgroup species from Lycosinae subfamily [*Alopecosa aculeata* (Clerck, 1757) and *Trochosa spinipalpis* (F. O. P.-Cambridge, 1895)]. Wider sampling of species from target species groups was done by mining public records from the Barcode of Life Database (BOLD) for COI analysis (212 specimens, 14 species). Detailed information about all the specimens used in the study is in Tables S1, S2 and S3 (SM1). Specimens were collected by hand or using pit-fall traps and preserved in absolute or 70% ethanol. No experiments were performed on live animals hence ethical approval was not required. Protected species were not utilized in this study and no permits were needed to collect the specimens.

### Morphological examination

Specimens were carefully examined to identify species, and the whole body and copulatory organs of the target species were photographed. Differences in structures of copulatory organs are traditionally used as the most reliable source of qualitative information for spider species delimitation^12^. Characters that are reported to be informative for *Pardosa*^61^ were photographed and measured, with the following modifications: CarLen and CarWid—dorsal length and width of cephalothorax in males and females, PatTib—length of patella + tibia in males and females, CymbLen—length of male palpal tarsus, CymbTip—length of tip of male palpal tarsus, BulbLen and BulbWid—length and width of bulbus in males, ApophLen—length of tegular apophysis for *P. riparia* males*,* SeptLen and SeptWid—length and width of septum in females. Males (*P. riparia* 15 specimens from each population; *P. palustris* 26 from Finland, 22 from FER) and females (*P. riparia* 15 specimens from each population; *P. palustris* 21 from Finland, 22 from FER) were analyzed separately. The measurement scheme can be found in Fig. S4 (SM1) and the measurements in Tables S4 and S5 (SM1). Principle Component Analysis (PCA) was used to analyze variation in the morphological data using RStudio with *pcomp* command^80^ and the results were visualized with *ggplot2* package^81^. Each character between two populations was checked for homoscedasticity in R (bartlett.test) and compared with ANCOVA using CarLen as a covariate to control for body size variation following the same rationale as in^57^. In addition, a t-test with unequal variances was utilized as an additional comparison between measurements. To visualize overlap between measured characters boxplots were produced with the *ggplot2* package.

### Mitochondrial DNA sequencing

The COI sequencing was performed in the Canadian Centre for DNA barcoding (CCDB). One leg from each specimen was placed into individual wells in a 96-well plate prefilled with ethanol and the plate was sent to CCDB. There, DNA was extracted and the COI gene (654 bp) was sequenced following standard protocol^82^. All collection data and specimens’ images were uploaded into BOLD together with successful sequences by corresponding Sample ID (SM1). In addition, publicly available sequences were included. For the full list of samples see Table S3 (SM1).

### ddRADseq library preparation

Genomic DNA (gDNA) was extracted with DNeasy Blood & Tissue Kit (Qiagen) according to the manufacturer’s protocol. One to four legs provided the required amount of gDNA for subsequent whole genome amplification (WGA). The rationale for using WGA was that the specimens were quite small and for morphological analysis it was important to keep them as intact as possible. Furthermore, the amount of gDNA extracted from the legs would not suffice for ddRADseq library preparation. The REPLI-g Mini Kit (Qiagen) for WGA was used according to the manufacturer’s instructions. It was assumed that WGA did not bias the results significantly as there was evidence of a negligible effect on the study outcome^83–85^ and examples of other studies successfully implementing WGA^86–89^.

Previously published protocols for the ddRADseq library preparation were followed^90,91^. Briefly, a combination of PstI and MseI restriction enzymes were used for shearing the DNA. Ligated adapters designed for this pair of enzymes were ligated and the samples were purified with AMPure XP magnetic beads (Agencourt). Samples were pooled into six sub-pools based on concentration measurements using the PicoGreen Kit (Molecular Probes). Automated size-selection was performed using Blue Pippin (Sage Science, 1.5% agarose cartridge) to obtain a library with a mean of 300 bp long fragments. Selected fragments were amplified with the Phusion High-Fidelity PCR Master Mix (Finnzymes) and the products were purified with AMPure XP magnetic beads. Prior to sequencing, the size distribution and concentration of sub-pools and the final pool were checked using Bioanalyzer (Agilent). The final library was sequenced on an Illumina HiSeq 2500 machine, 100 PE in FIMM, (Institute for Molecular Medicine, Finland). Raw DNA reads from ddRADseq are available at the NCBI Sequence Read Archive (BioProject ID: PRJNA345307 and PRJNA595572).

### ddRADseq data bioinformatics

Quality control was performed with FastQC^92^. Paired-end reads were assembled de novo using the ipyrad workflow (https://ipyrad.readthedocs.io/). The software provides strict filtering steps to ensure the absence of paralogs and low-quality base calls in the assembly. The most important parameter altered from the default was the minimum number of samples per locus (*m*). Test datasets were assembled with different combination of the parameter to find optimum values as well as to investigate the influence of missing data on consecutive analyses. Values of *m* from a minimum of three to a maximum of 22 were tested. Two types of datasets were assembled, the first included all species with an outgroup and the second contained only target species with their closest relatives. The first dataset was used for phylogenetic inference and *p*-distance computation and the second set for species delimitation and population genetic analyses. All strict filtering steps allowed for additional control of possible WGA bias. The proportion of missing data was calculated with Mesquite version 3.51^93^.

### Phylogenetic analyses and haplotype networks

Phylogenetic analyses were required to reveal historical relationships among taxa and to test the validity of the prevailing species hypotheses. Maximum likelihood (ML) trees for ddRADseq datasets were inferred with the IQ-Tree program^94^ with 5000 ultrafast bootstrap support for branches^95^. Haplotype networks for COI were computed with a modified script in RStudio (originally from https://johnbhorne.wordpress.com/2016/09/15/still-making-haplotype-networks-the-old-way-how-to-do-it-in-r/) using packages *ape*/5.3^96^ and *pegas*/0.11^97^ and PopArt/1.7 using the TCS method^98,99^. Maximum likelihood trees for COI datasets were constructed using MEGA7^100^ with bootstrap support estimated from 500 replicates and the GTR + G substitution model.

### Population genetics

STRUCTURE version 2.3.1 was used to detect admixture between populations^101^. To optimize the runs, StrAuto program^102^ was used with the following parameters: 500,000 replicates with 50,000 burn-in, 10 replicates for each K (K = 1–6 for the *pullata* group and K = 1–4 for the *monticola* group). An optimal K was estimated with STRUCTURE HARVESTER^103^ based on the ad hoc ∆*K* statistics^104^. Replicates were permuted in the program CLUMPP^105^ and bar plots were visualized with the program Distruct^106^.

### Species delimitation

For COI datasets, species discovery methods were utilized^7^ to assess the overall efficiency of DNA barcodes for species delimitation purposes in the focal species. Firstly, the BIN system was used for the initial assignment of specimens to operational taxonomic units (OTUs) as implemented in BOLD^11^. The species ID trees for all available *P. palustris* and *P. riparia* are presented in the SM3.

Secondly, a browser version of bPTP^107^ was utilized with the following parameters: unrooted tree, 100,000 Markov chain Monte Carlo (MCMC) generation, 0.1 burn-in. Input trees used were COI ML trees built with IQ-Tree for five datasets: *P. riparia* only (n = 21), *P. palustris* only (n = 45), *pullata* group (n = 117), *monticola* group (95 specimens) and combined *pullata* and *monticola* groups (n = 212).

The third species discovery method used was a browser version of GMYC^108^. The same datasets employed for bPTP were utilized. Ultrametric trees required as GMYC input were computed in BEAST 2.5^109^ with Strict Clock Model, 10 million MCMC chain length, 100,000 pre-burn. Priors: coalescent constant population model, uniform frequency parameter, exponential gamma rate, population size 1/X, uniform rates from 1 to 100. Single threshold was used for the GMYC run^110^.

For species delimitation with the ddRADseq data, Bayes-factor species delimitation (BFD*) was implemented in SNAPP, BEAST 2.60^34^. Specimens were subsampled from the initial ddRADseq assemblies to form two datasets. The first dataset included 18 specimens from the *pullata* group with 543 unlinked SNPs (40.6% missing data) while the second included ten specimens from the *monticola* group with 1153 unlinked SNPs, (1% missing data). Dataset selection was based on a minimum missing data parameter. Two alternative scenarios were tested for both datasets, where representatives of *P. riparia* or *P. palustris* from different populations belong to the same or different species. Bayes factor was calculated to evaluate the alternative hypotheses. Run parameters and specimens included in each dataset can be found in SM1.

Species trees for ddRADseq datasets were estimated in a coalescent framework using all data with the program SVDquartets^111^ implemented in PAUP*^112^. Sub-sampling was done at 10,000 quartets. Sampled quartets were assembled into a species tree using a variant of Quartet FM^113^, which was the implementation recommended by the developers of SVDquartets.

### Pairwise distances

The estimation of *p*-distances was performed using PAUP* for the ddRADseq datasets. The goal of the analysis was to determine if the levels of variation in ddRADseq corresponded to the different species hypotheses derived from the COI data, morphology and ddRADseq data analyses. The *p*-distances for ddRADseq data were calculated for the dataset that included all species and an outgroup. Measured *p*-distances were grouped into intraspecific, interspecific and *p*-distances between compared geographically distant populations. Mean, quartiles and 95% confidence intervals were calculated and absolute values of *p*-distances were visualized as boxplots using *ggplot2* package in RStudio for ddRADseq datasets with different *m* values. In addition, frequencies of *p*-distance values within species, between target populations and between distinct species were computed and plotted using *ggplot2* package in RStudio.

## Supplementary Information

Supplementary Information 1.

Supplementary Information 2.

Supplementary Information 3.

Supplementary Information 4.

## Data Availability

Raw ddRADseq reads can be found in NCBI Sequence Read Archive (BioProject ID: PRJNA345307 and PRJNA595572).
