# Supplementary material for: Relevance of ddRADseq method for species and population delimitation of closely related and widely distributed wolf spiders (Araneae, Lycosidae)
Source: Sci Rep. 2021 Jan 26;11:2177. doi: 10.1038/s41598-021-81788-2 (PMC7838170; doi:10.1038/s41598-021-81788-2)
Supplement: Supplementary file 1 — Supplementary Information 1. [file 41598_2021_81788_MOESM1_ESM.pdf]

Relevance of ddRADseq method for species and population delimitation of closely related and widely distributed wolf spiders (Araneae, Lycosidae)

*Vladislav Ivanov[1], Yuri Marusik[2,3], Julien Pétillon[4], Marko Mutanen[1]

[1]Department of Ecology and Genetics, University of Oulu, Oulu, Finland

[2]Institute for Biological Problems of the North, RAS, Magadan, Russia

[3]Department of Zoology & Entomology, University of the Free State, Bloemfontein 9300, South Africa

[4]UMR CNRS ECOBIO, Université de Rennes 1, Rennes, France

*Corresponding author email: vladislav.ivanov@oulu.fi
Yuri Marusik yurmar@mail.ru
Julien Pétillon julien.petillon@univ-rennes1.fr
Marko Mutanen marko.mutanen@oulu.fi

a.

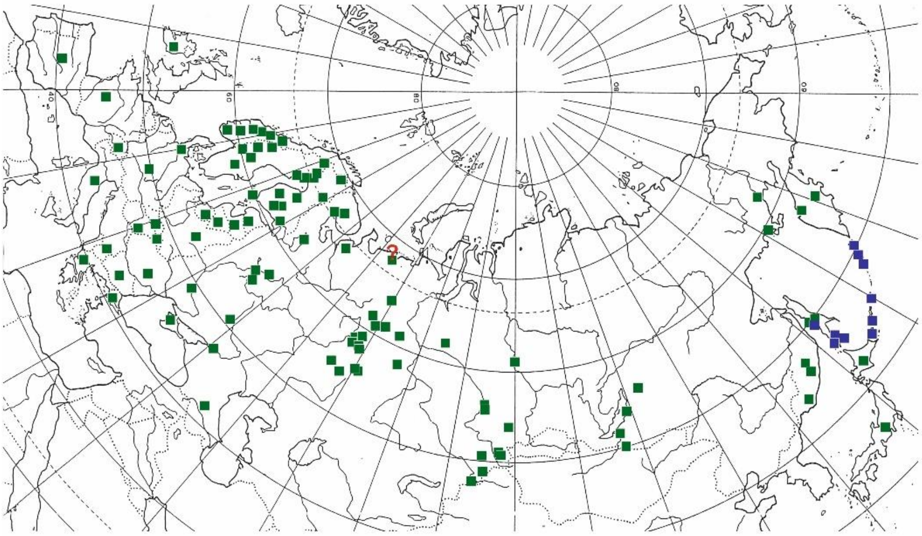

b.

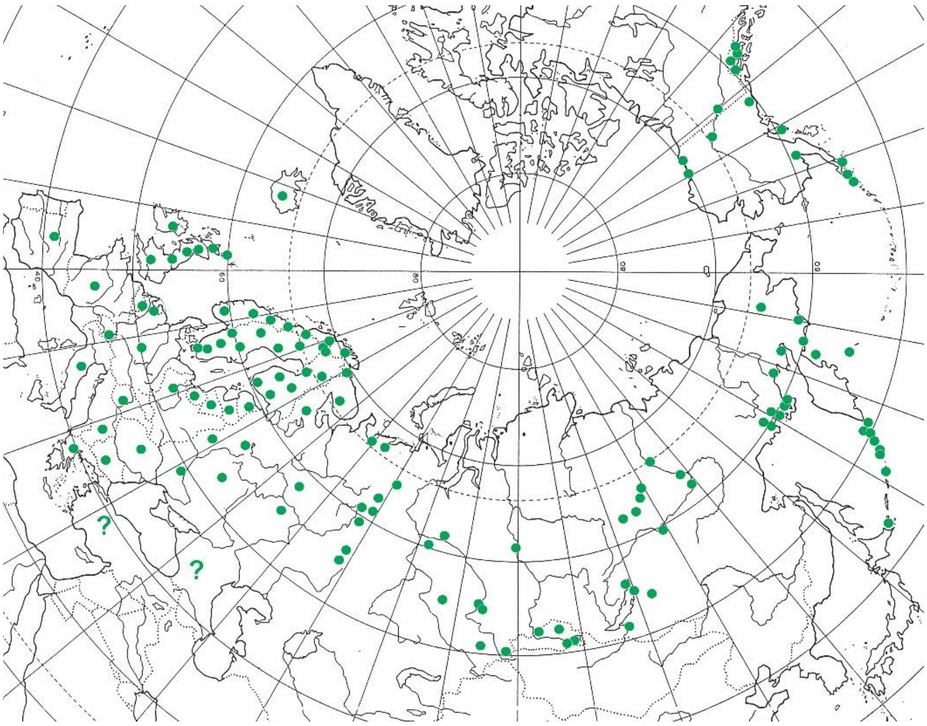

Figure S1. Distribution map of *P. riparia* and *P. palustris* based on published data and personal references. a. *P. riparia*: green squares - published records, blue squares - unpublished records, red question mark - doubtful records; b. *P. palustris*: green circles - published records, green question marks - doubtful records.

References

Azarkina, G. N., Lyubechanskii, I. I., Trilikauskas, L. A., Dudko, R. Y., Bespalov, A. N. & Mordkovich, V. G. 2018. A check-list and zoogeographic analysis of the spider fauna (Arachnida: Aranei) of Novosibirsk Area (West Siberia, Russia). *Arthropoda Selecta* **27**(1): 73-93.

Azarkina, G. N. & Trilikauskas, L. A. 2013. Spider fauna (Aranei) of the Russian Altai, part II: families Gnaphosidae, Hahniidae, Linyphiidae, Liocranidae and Lycosidae. *Euroasian Entomological Journal* **12**: 51-67.

Ballarin F., Marusik Yu.M., Omelko M.M., Koponen S. 2012. On *Pardosa monticola* species-group (Araneae: Lycosidae) from Middle Asia. – Arthropoda Selecta. 21(2): 161-182.

Danilov, S. N. 2008. *Catalogue of the spiders (Arachnida, Aranei) of Transbaikalia*. Buryatian Scientific Center Siberian Branch Russian Academy of Sciences Press Ulan-Ude, 108 pp.

Esyunin, S. L. & Efimik, V. E. 1996. *Catalogue of the Spiders (Arachnida, Aranei) of the Urals*. KMK Scientific Press Moscow, 228 pp.

Marusik Yu.M., Logunov D.V. & Koponen S. 2000. Spiders of Tuva, South Siberia. – IBPN FEB RAS, 252 pp.

Marusik Yu.M., Eskov K.Yu. & Kim J.P. 1992. A check-list of spiders (Aranei) of North-East Asia. - Korean Arachnol., 8(1/2): 129-158.

Marusik Yu.M., Eskov K.Yu., Koponen S. & Vinokurov N.N. 1993.A check-list of the spiders (Aranei) of Yakutia, Siberia. - Arthropoda Selecta, 2(2): 63-79.

Marusik Yu.M., Eskov K.Yu., Logunov D.V. & Basarukin A.M. 1992. A check-list of spiders (Arachnida Aranei) from Sakhalin and Kurile Islands. - Arthropoda Selecta, 1(4): 73-85.

Marusik Yu.M., Rybalov L.B., Koponen S. & Tanasevitch A.V. 2002. Spiders (Aranei) of Middle Siberia, an updated check-list with a special reference to the Mirnoye Field Station. - Arthropoda Selecta 10(4): 323-350.

Marusik Yu.M., Omelko M.M., Ryabukhin A.S. 2013. New data on spiders (Aranei) of eastern Koryakia, Kamchatka Peninsula. – Arthropoda Selecta, 22(3): 363-377.

Databases:

World Spider Catalog https://wsc.nmbe.ch/search

Fauna Europea https://fauna-eu.org/

Spiders of Europe https://araneae.nmbe.ch/

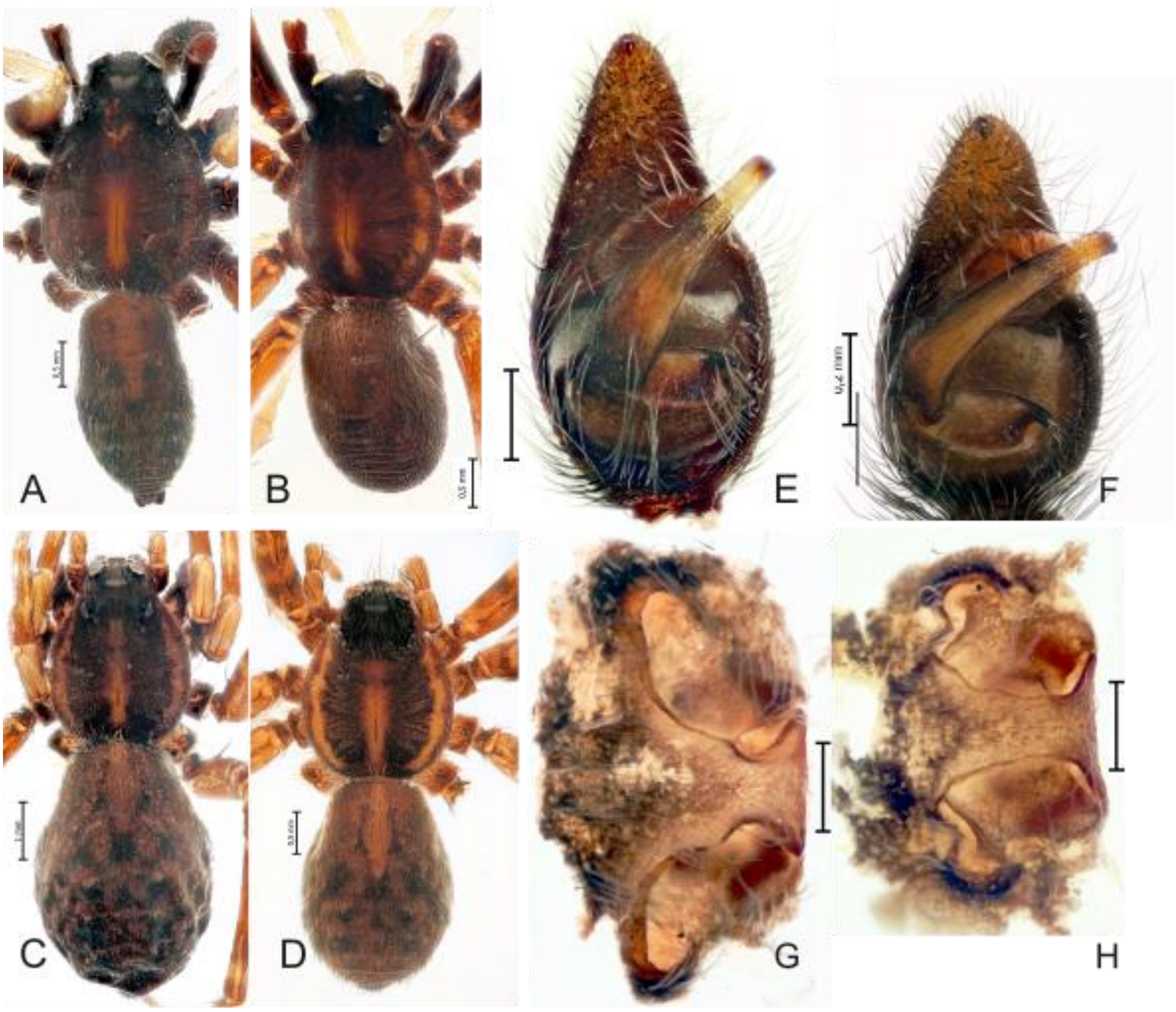

Figure S2. *P. riparia* morphology. Russian population – A,C,E,G; Finnish population – B,D,F,H. Males – A,B; females – C,D; male palp – E,F; epigyna – G,H.

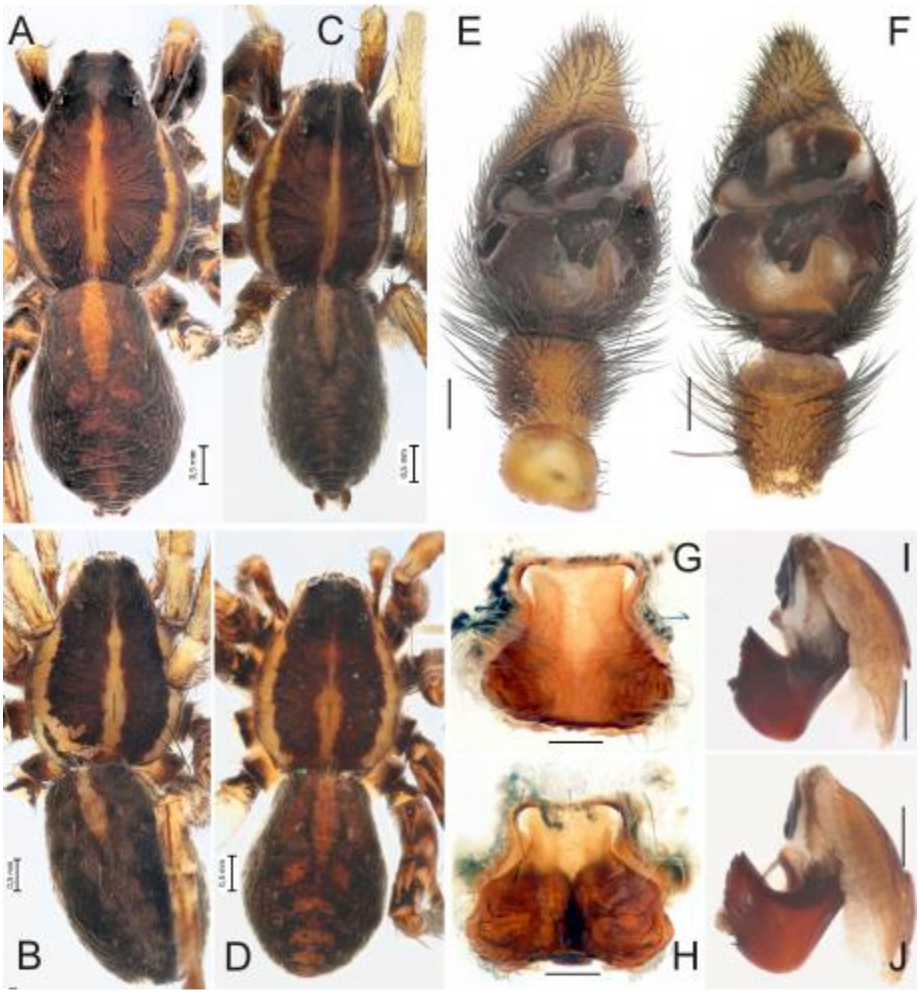

Figure S3. *P. palustris* morphology. Russian population – A,B,E,G,I; Finnish population – C,D,F,H,J. Males – A,C; females – B,D; male palp – E,F; terminal apophysis – I,J; epigyna – G,H.

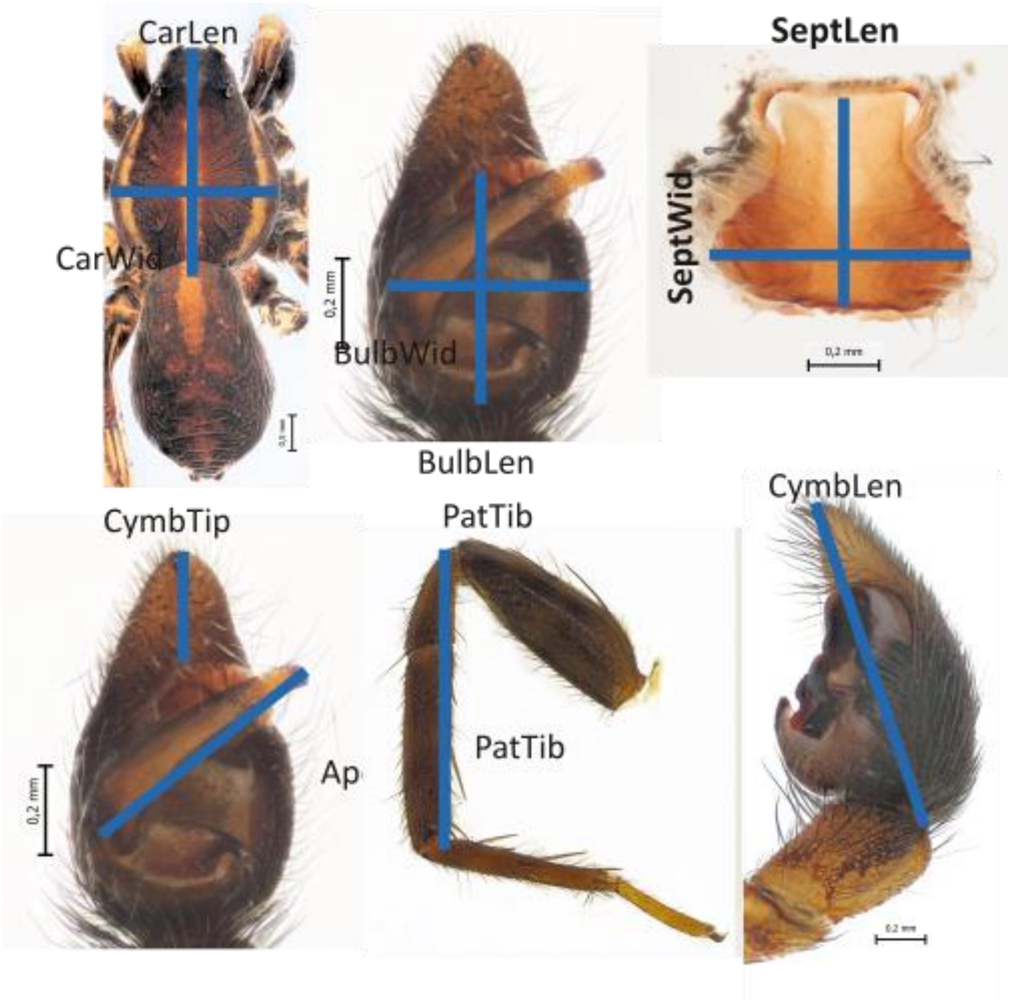

Figure S4. Measurement scheme. CarLen and CarWid - dorsal length and width of cephalothorax in males and females, PatTib - length of patella+tibia in males and females, CymbLen - length of cymbium in males, CymbTip - length of cymbium tip in males, BulbLen and BulbWid - length and width of bulbus in males, Ap - length of tegular apophysis for *P. riparia* males, SeptLen and SeptWid - length and width of septum in females.

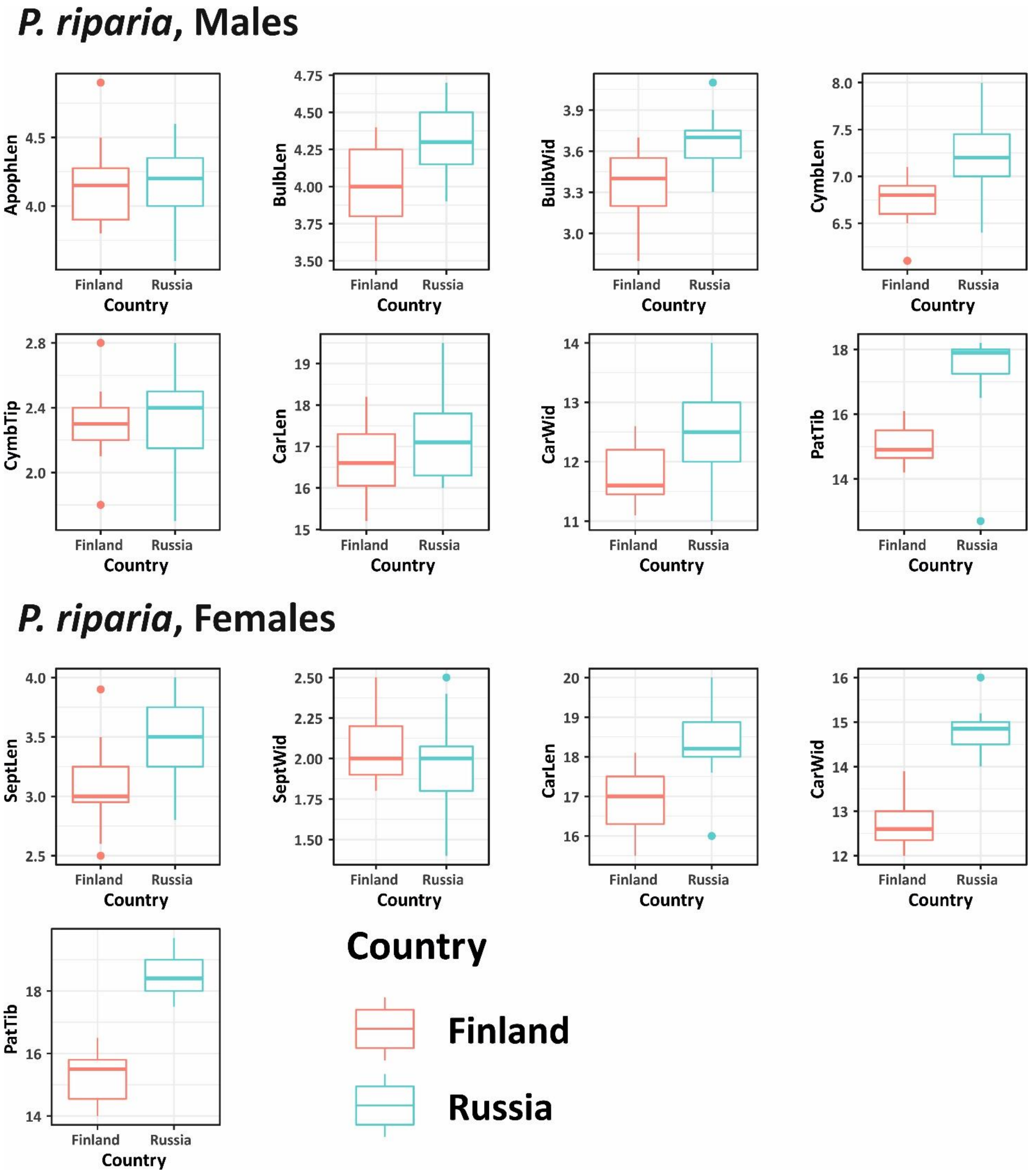

Figure S5. Boxplots of measured characters for *P. riparia*. CarLen and CarWid - dorsal length and width of cephalothorax in males and females, PatTib - length of patella+tibia in males and females, CymbLen - length of cymbium in males, CymbTip - length of cymbium tip in males, BulbLen and BulbWid - length and width of bulbus in males, ApophLen - length of tegular apophysis for *P. riparia* males, SeptLen and SeptWid - length and width of septum in females.

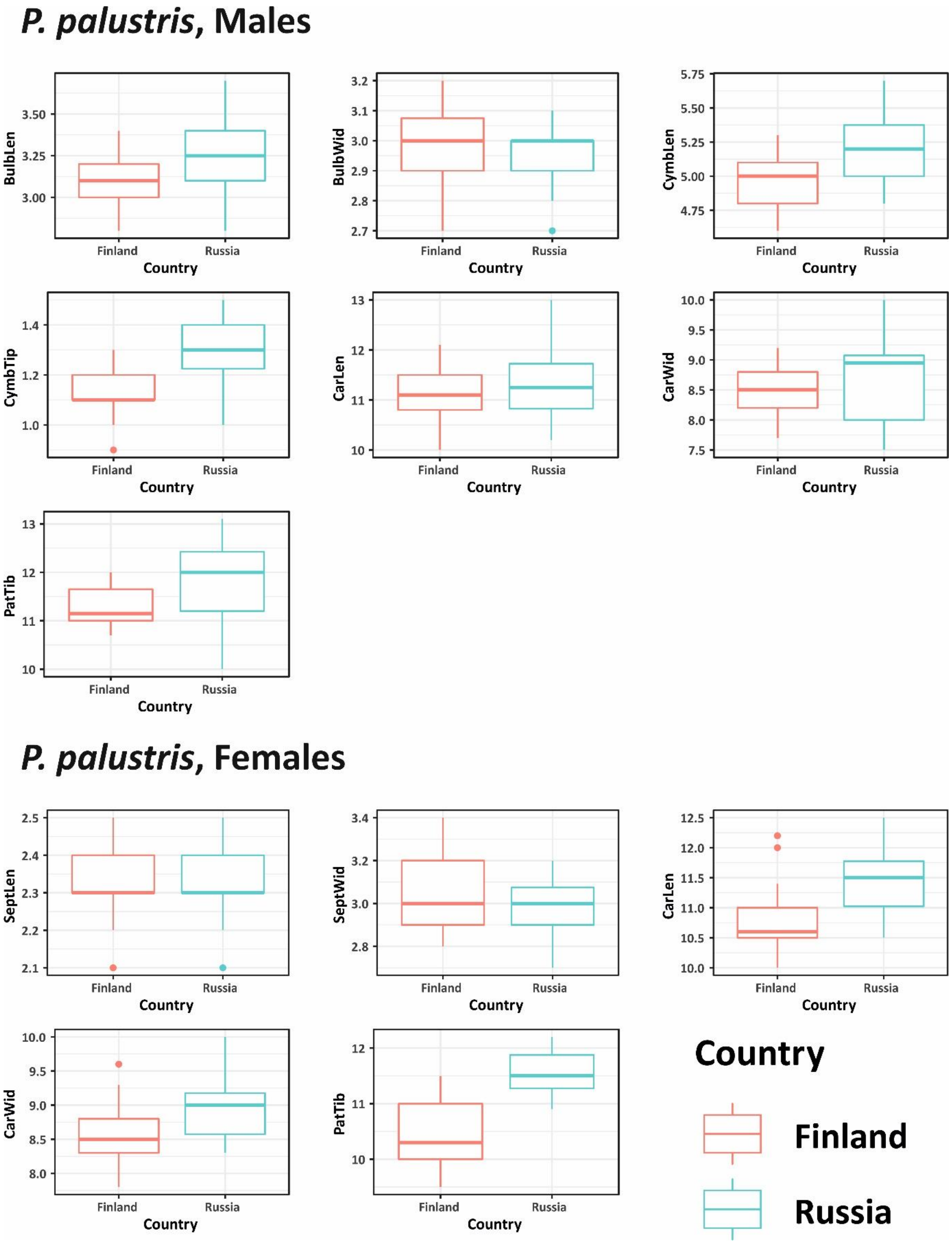

Figure S6. Boxplots of measured characters for *P. palustris*. CarLen and CarWid - dorsal length and width of cephalothorax in males and females, PatTib - length of patella+tibia in males and females, CymbLen - length of cymbium in males, CymbTip - length of cymbium tip in males, BulbLen and BulbWid - length and width of bulbus in males, SeptLen and SeptWid - length and width of septum in females.

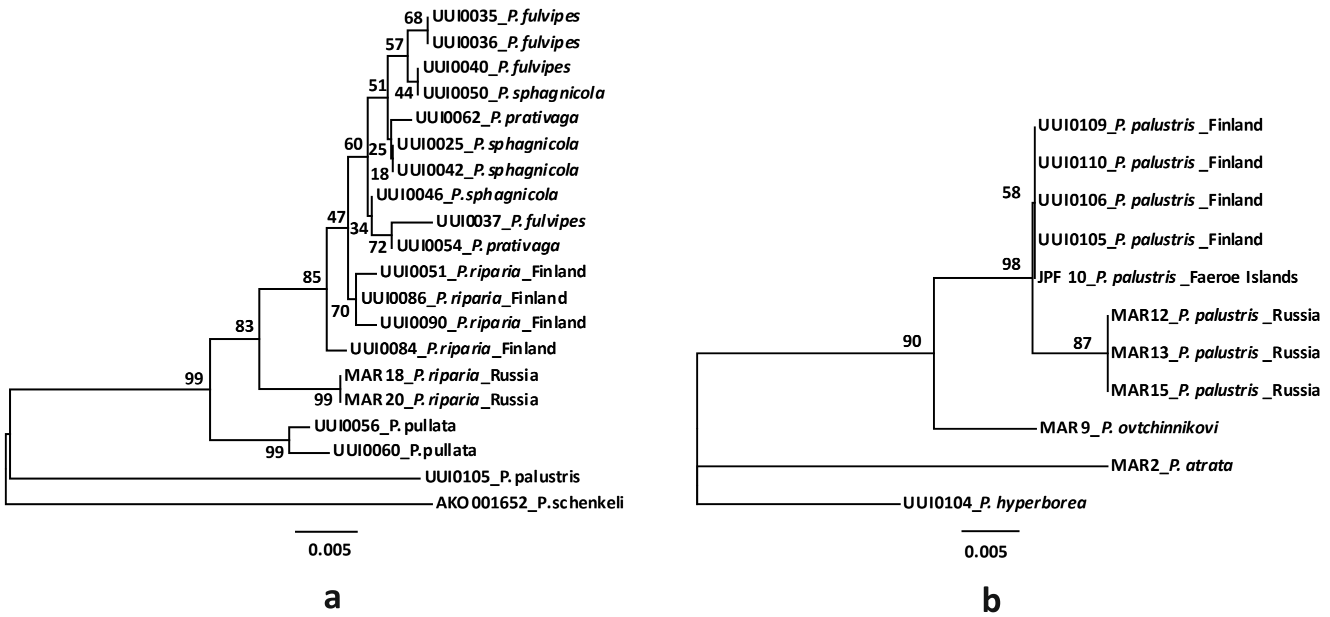

Figure S7. Maximum likelihood trees for COI: a –*pullata* species group, b – for *monticola* species group. Numbers at nodes indicate bootstrap support. Samples included here were attempted to be sequenced with ddRADseq protocol.

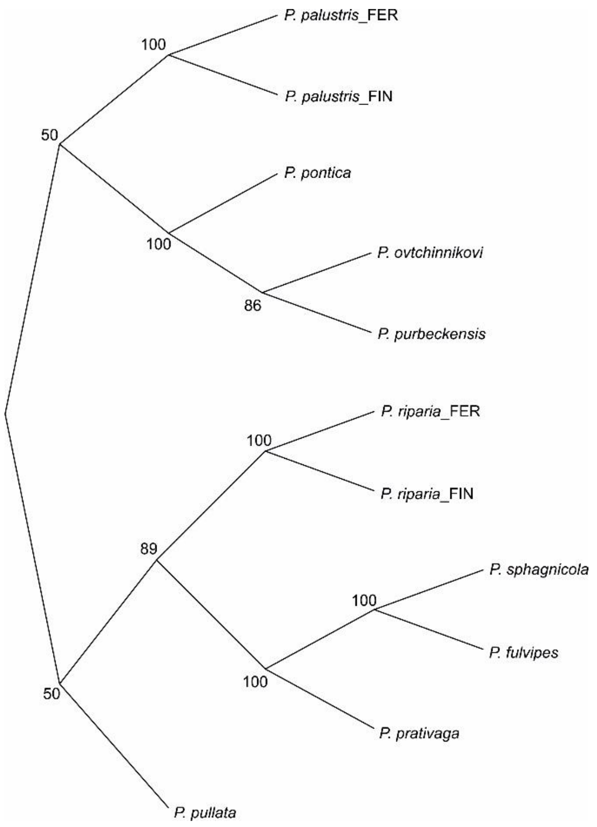

Figure S8. Species tree calculated in SVDquartets. Numbers indicate bootstrap support for the splits. The dataset used: Rip_Pal_IN_m6c85.

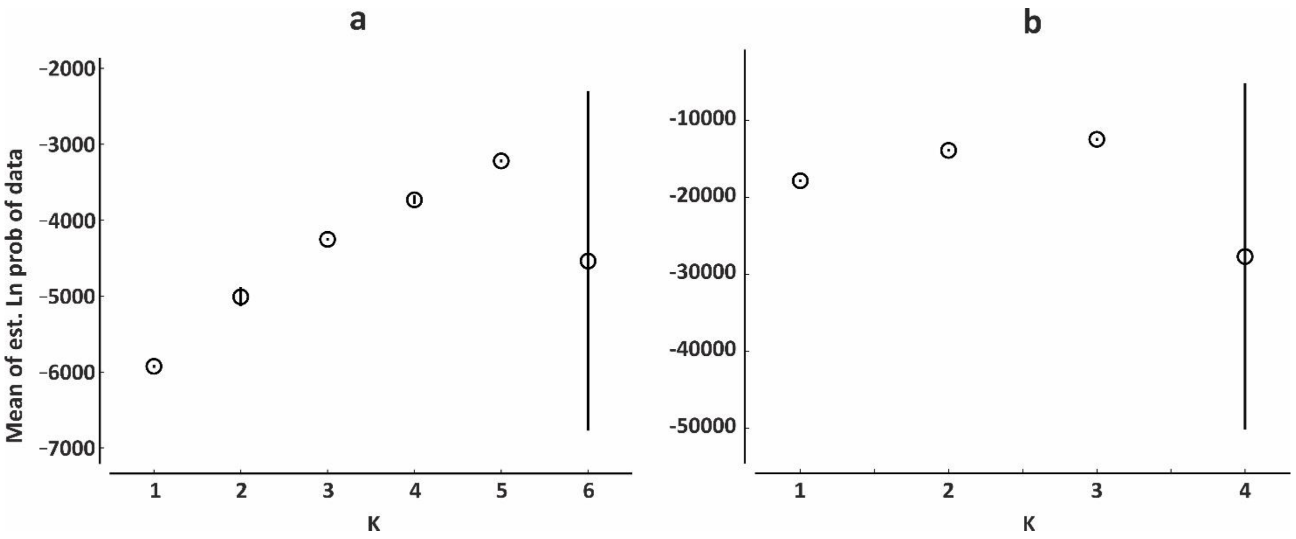

Figure S9. Mean of estimated Ln probability of the data through: a. K 1 to 6, *pullata* group; b. K 1 to 4, *P. palustris* and *P. ovtchinnikovi.* Vertical lines indicate standard deviation of mean.

Table S1. Data for specimens used in ddRADseq analysis

Species	ID	Gender	Country	Location	Date	Collector	Latitude	Longitude	Included in SNAPP analysis
A. aculeata	UUI0003	Male	Finland	Kilpisjärvi, Siilasjärvi	14.7.2015	Vladislav Ivanov	69.081813	20.750465	Not included
P. atrata	MAR_1	Male	Russia	Magadan, Snazhnaya Dolina village, Duktcha River	23.6.15	Yuri Marusik	59.745	150.848	Not included
P. fulvipes	UUI0035	Male	Finland	Tvärminne	11.6.2015	Vladislav Ivanov	59.843292	23.212050	Included
P. fulvipes	UUI0036	Male	Finland	Tvärminne	3.7.2015	Vladislav Ivanov	59.843292	23.212050	Included
P. fulvipes	UUI0037	Male	Finland	Oulu, Kaijonlahti	3.7.2015	Vladislav Ivanov	65.064433	25.490416	Included
P. fulvipes	UUI0040	Male	Finland	Oulu, Kaijonlahti	14.7.2015	Vladislav Ivanov	65.064433	25.490416	Included
P. hyperborea	UUI0104	Male	Finland	Kilpisjärvi, Lake shore	12-16.07.2015	Vladislav Ivanov	69.081813	20.750465	Not included
P. ovtchinnikovi	MAR_6	Female	Tajikistan	Khatlon Area, Shaartuz district, Khushody	20.4.15	Yuri Marusik	37.153	68.071	Not included
P. ovtchinnikovi	MAR_8	Female	Tajikistan	Khatlon Area, Shaartuz district, Khushody	20.4.15	Yuri Marusik	37.153	68.071	Included
P. ovtchinnikovi	MAR_9	Male	Tajikistan	Khatlon Area, Shaartuz district, Khushody	20.4.15	Yuri Marusik	37.153	68.071	Included
P. palustris	JPF_10	Female	Faroe Islands	Torshvan Island	13-17.6.2012	Julien Pétillon	62.02	-6.77	Not included
P. palustris	JPF_11	Male	Faroe Islands		6.2012	Julien Pétillon	62.02	-6.77	Not included
P. palustris	MAR_11	Male	Russia	Magadan, Snazhnaya Dolina village, Duktcha River	23.6.15	Yuri Marusik	59.745	150.848	Included
P. palustris	MAR_12	Female	Russia	Magadan, Snazhnaya Dolina village, Duktcha River	23.6.15	Yuri Marusik	59.745	150.848	Included
P. palustris	MAR_13	Male	Russia	Magadan, Snazhnaya Dolina village, Duktcha River	23.6.15	Yuri Marusik	59.745	150.848	Included
P. palustris	MAR_14	Female	Russia	Magadan, Snazhnaya Dolina village, Duktcha River	23.6.15	Yuri Marusik	59.745	150.848	Included
P. palustris	MAR_15	Male	Russia	Magadan, Snazhnaya Dolina village, Duktcha River	23.6.15	Yuri Marusik	59.745	150.848	Included
P. palustris	UUI0105	Male	Finland	Kilpisjärvi, Lake shore	12-16.7.2015	Vladislav Ivanov	69.081813	20.750465	Included
P. palustris	UUI0106	Female	Finland	Kilpisjärvi, Saana	12-16.7.2015	Vladislav Ivanov	69.056241	20.796986	Included
P. palustris	UUI0109	Male	Finland	Kiiminki	29.6.15	Vladislav Ivanov	65.1478	25.8383	Included
P. palustris	UUI0110	Male	Finland	Oulu, Kaijonlahti	2-4.7.2015	Vladislav Ivanov	65.064433	25.490416	Included
P. pontica	SHAF_2	Female	Iran	Mazandaran	16.7.16	Sepideh Shafaie	36.584444	52.804444	Not included
P. prativaga	UUI0054	Male	Finland	Tvärminne	6.8.2015	Vladislav Ivanov	59.843292	23.212050	Included
P. prativaga	UUI0062	Female	Finland	Tvärminne	15.6.2015	Vladislav Ivanov	59.843292	23.212050	Included
P. pullata	UUI0056	Male	Finland	Kiiminki	19.6.2015	Vladislav Ivanov	59.843292	23.212050	Included
P. pullata	UUI0060	Male	Finland	Oulu, Kaijonlahti	16.8.2011	Vladislav Ivanov	59.843292	23.212050	Included
P. purbeckensis	JPF_16	Female	France	La Frénaye	2.7.13	Julien Pétillon	49.51	0.57	Not included
P. riparia	MAR_18	Male	Russia	Magadan, Snazhnaya Dolina village, Duktcha River	23.6.15	Yuri Marusik	59.745	150.848	Included

P. riparia	MAR_20	Male	Russia	Magadan, Snazhnaya Dolina village, Duktcha River	23.6.15	Yuri Marusik	59.745	150.848	Included
P. riparia	UUI0051	Male	Finland	Kiiminki	19.6.2015	Vladislav Ivanov	65.1478	25.8383	Included
P. riparia	UUI0084	Male	Finland	Kiiminki	19.6.2015	Vladislav Ivanov	65.1478	25.8383	Included
P. riparia	UUI0086	Female	Finland	Kiiminki	26.6.2015	Vladislav Ivanov	65.1478	25.8383	Included
P. riparia	UUI0090	Female	Finland	Kiiminki	19.6.2015	Vladislav Ivanov	65.1478	25.8383	Included
P. sphagnicola	UUI0025	Female	Finland	Kiiminki	11.6.2015	Vladislav Ivanov	65.1478	25.8383	Included
P. sphagnicola	UUI0042	Male	Finland	Kiiminki	11.6.2015	Vladislav Ivanov	65.1478	25.8383	Included
P. sphagnicola	UUI0046	Male	Finland	Tvärminne	1.7.2015	Vladislav Ivanov	65.1478	25.8383	Included
P. sphagnicola	UUI0050	Male	Finland	Kiiminki	3.7.2015	Vladislav Ivanov	65.064433	25.490416	Included
T. spinipalpis	UUI0076	Male	Finland	Kiiminki	19.6.2015	Vladislav Ivanov	65.1478	25.8383	Not included

Table S2. Data for specimens used in morphometry

ID	Species	Gender	Country	Place	Latitude	Longitude	Date
JPF_10	*Pardosa palustris*	Female	Faroeae Islands		62.02	-6.77	13-17.6.2012
JPF_11	*Pardosa palustris*	Male	Faroeae Islands		62.02	-6.77	6.2012
JPF_12	*Pardosa palustris*	Female	Faroeae Islands		62.02	-6.77	6.2012
JPF_13	*Pardosa palustris*	Male	Great Britain	Essex	51.75	0.83	5.6.05
JPF_14	*Pardosa palustris*	Male	France		48.55	-1.92	12.5.2010
MAR_11	*Pardosa palustris*	Male	Russia	Magadan	59.745	150.848	23.6.15
MAR_12	*Pardosa palustris*	Female	Russia	Magadan	59.745	150.848	23.6.15
MAR_13	*Pardosa palustris*	Male	Russia	Magadan	59.745	150.848	23.6.15
MAR_14	*Pardosa palustris*	Female	Russia	Magadan	59.745	150.848	23.6.15
UUI0105	*Pardosa palustris*	Male	Finland	Kilpisjärvi	69.081813	20.750465	12-16.07.2015
UUI0106	*Pardosa palustris*	Female	Finland	Kilpisjärvi	69.056241	20.796986	12-16.07.2015
UUI0107	*Pardosa palustris*	Male	Finland	Oulu	65.040300	25.416001	28.05.-03.06.2015
UUI0108	*Pardosa palustris*	Male	Finland	Kiiminki	65.1478	25.8383	07.2015
UUI0109	*Pardosa palustris*	Male	Finland	Kiiminki	65.1478	25.8383	07.2015
UUI0110	*Pardosa palustris*	Male	Finland	Oulu	65.0644	25.4904	02-04.07.2015
UUI0111	*Pardosa palustris*	Male	Finland	Kiiminki	65.1478	25.8383	07.2015
UUI0307	*Pardosa riparia*	Male	Finland		65.14	25.83	6.2015-7.2015
UUI0308	*Pardosa riparia*	Male	Finland		65.14	25.83	6.2015-7.2015
UUI0309	*Pardosa riparia*	Male	Finland		65.14	25.83	6.2015-7.2015
UUI0310	*Pardosa riparia*	Male	Finland		65.14	25.83	6.2015-7.2015
UUI0311	*Pardosa riparia*	Male	Finland		65.14	25.83	6.2015-7.2015
UUI0312	*Pardosa riparia*	Male	Finland		65.14	25.83	6.2015-7.2015
UUI0313	*Pardosa riparia*	Male	Finland		65.14	25.83	6.2015-7.2015
UUI0314	*Pardosa riparia*	Male	Finland		65.14	25.83	6.2015-7.2015
UUI0315	*Pardosa riparia*	Male	Finland		65.14	25.83	6.2015-7.2015
UUI0316	*Pardosa riparia*	Male	Finland		65.14	25.83	6.2015-7.2015
UUI0317	*Pardosa riparia*	Male	Finland		65.14	25.83	6.2015-7.2015
UUI0318	*Pardosa riparia*	Male	Finland		65.14	25.83	6.2015-7.2015
UUI0319	*Pardosa riparia*	Male	Finland		65.14	25.83	6.2015-7.2015
UUI0320	*Pardosa riparia*	Male	Finland		65.14	25.83	6.2015-7.2015
UUI0321	*Pardosa riparia*	Male	Finland		65.14	25.83	6.2015-7.2015
UUI0322	*Pardosa riparia*	Female	Finland		65.14	25.83	6.2015-7.2015
UUI0323	*Pardosa riparia*	Female	Finland		65.14	25.83	6.2015-7.2015
UUI0324	*Pardosa riparia*	Female	Finland		65.14	25.83	6.2015-7.2015
UUI0325	*Pardosa riparia*	Female	Finland		65.14	25.83	6.2015-7.2015
UUI0326	*Pardosa riparia*	Female	Finland		65.14	25.83	6.2015-7.2015
UUI0327	*Pardosa riparia*	Female	Finland		65.14	25.83	6.2015-7.2015
UUI0328	*Pardosa riparia*	Female	Finland		65.14	25.83	6.2015-7.2015
UUI0329	*Pardosa riparia*	Female	Finland		65.14	25.83	6.2015-7.2015
UUI0330	*Pardosa riparia*	Female	Finland		65.14	25.83	6.2015-7.2015
UUI0331	*Pardosa riparia*	Female	Finland		65.14	25.83	6.2015-7.2015
UUI0332	*Pardosa riparia*	Female	Finland		65.14	25.83	6.2015-7.2015

UUI0333	*Pardosa riparia*	Female	Finland		65.14	25.83	6.2015-7.2015
UUI0334	*Pardosa riparia*	Female	Finland		65.14	25.83	6.2015-7.2015
UUI0335	*Pardosa riparia*	Female	Finland		65.14	25.83	6.2015-7.2015
UUI0336	*Pardosa riparia*	Female	Finland		65.14	25.83	6.2015-7.2015
UUI0337	*Pardosa riparia*	Male	Russia		59.71	151.00	1999-2000
UUI0338	*Pardosa riparia*	Male	Russia		59.71	151.00	1999-2000
UUI0339	*Pardosa riparia*	Male	Russia		59.71	151.00	1999-2000
UUI0340	*Pardosa riparia*	Male	Russia		59.71	151.00	1999-2000
UUI0341	*Pardosa riparia*	Male	Russia		59.71	151.00	1999-2000
UUI0342	*Pardosa riparia*	Male	Russia		59.71	151.00	1999-2000
UUI0343	*Pardosa riparia*	Male	Russia		59.71	151.00	1999-2000
UUI0344	*Pardosa riparia*	Male	Russia		59.71	151.00	1999-2000
UUI0345	*Pardosa riparia*	Male	Russia		59.71	151.00	1999-2000
UUI0346	*Pardosa riparia*	Male	Russia		59.71	151.00	1999-2000
UUI0347	*Pardosa riparia*	Male	Russia		59.71	151.00	1999-2000
UUI0348	*Pardosa riparia*	Male	Russia		59.71	151.00	1999-2000
UUI0349	*Pardosa riparia*	Male	Russia		59.71	151.00	1999-2000
UUI0350	*Pardosa riparia*	Male	Russia		59.71	151.00	1999-2000
UUI0351	*Pardosa riparia*	Male	Russia		59.71	151.00	1999-2000
UUI0352	*Pardosa riparia*	Female	Russia		59.71	151.00	1999-2000
UUI0353	*Pardosa riparia*	Female	Russia		59.71	151.00	1999-2000
UUI0354	*Pardosa riparia*	Female	Russia		59.71	151.00	1999-2000
UUI0355	*Pardosa riparia*	Female	Russia		59.71	151.00	1999-2000
UUI0356	*Pardosa riparia*	Female	Russia		59.71	151.00	1999-2000
UUI0357	*Pardosa riparia*	Female	Russia		59.71	151.00	1999-2000
UUI0358	*Pardosa riparia*	Female	Russia		59.71	151.00	1999-2000
UUI0359	*Pardosa riparia*	Female	Russia		59.71	151.00	1999-2000
UUI0361	*Pardosa riparia*	Female	Russia		59.71	151.00	1999-2000
UUI0362	*Pardosa riparia*	Female	Russia		59.71	151.00	1999-2000
UUI0363	*Pardosa riparia*	Female	Russia		59.71	151.00	1999-2000
UUI0364	*Pardosa riparia*	Female	Russia		59.71	151.00	1999-2000
UUI0365	*Pardosa riparia*	Female	Russia		59.71	151.00	1999-2000
UUI0366	*Pardosa riparia*	Female	Russia		59.71	151.00	1999-2000
UUI0367	*Pardosa palustris*	Male	Finland	Kilpisjärvi	69.057	20.812	12.7.2015-16.7.2015
UUI0368	*Pardosa palustris*	Male	Finland	Kilpisjärvi	69.057	20.812	12.7.2015-16.7.2015
UUI0369	*Pardosa palustris*	Male	Finland	Kilpisjärvi	69.057	20.812	12.7.2015-16.7.2015
UUI0370	*Pardosa palustris*	Male	Finland	Kilpisjärvi	69.057	20.812	12.7.2015-16.7.2015
UUI0371	*Pardosa palustris*	Male	Finland	Kilpisjärvi	69.057	20.812	12.7.2015-16.7.2015
UUI0372	*Pardosa palustris*	Male	Finland	Kilpisjärvi	69.057	20.812	12.7.2015-16.7.2015
UUI0373	*Pardosa palustris*	Male	Finland	Kilpisjärvi	69.057	20.812	12.7.2015-16.7.2015
UUI0374	*Pardosa palustris*	Male	Finland	Kiiminki	65.147	25.838	8.6.2015-29.6.2015
UUI0375	*Pardosa palustris*	Male	Finland	Kiiminki	65.147	25.838	8.6.2015-29.6.2015
UUI0376	*Pardosa palustris*	Male	Finland	Kiiminki	65.147	25.838	8.6.2015-29.6.2015
UUI0377	*Pardosa palustris*	Male	Finland	Kiiminki	65.147	25.838	8.6.2015-29.6.2015
UUI0378	*Pardosa palustris*	Male	Finland	Kiiminki	65.147	25.838	8.6.2015-29.6.2015

UUI0379	*Pardosa palustris*	Male	Finland	Kiiminki	65.147	25.838	8.6.2015-29.6.2015
UUI0380	*Pardosa palustris*	Male	Finland	Kiiminki	65.147	25.838	8.6.2015-29.6.2015
UUI0381	*Pardosa palustris*	Male	Finland	Oulu	65.064	25.490	30.6.2015-2.7.2015
UUI0382	*Pardosa palustris*	Male	Finland	Oulu	65.064	25.490	30.6.2015-2.7.2015
UUI0383	*Pardosa palustris*	Male	Finland	Oulu	65.064	25.490	30.6.2015-2.7.2015
UUI0384	*Pardosa palustris*	Male	Finland	Oulu	65.064	25.490	30.6.2015-2.7.2015
UUI0385	*Pardosa palustris*	Male	Finland	Oulu	65.064	25.490	30.6.2015-2.7.2015
UUI0386	*Pardosa palustris*	Male	Finland	Oulu	65.064	25.490	30.6.2015-2.7.2015
UUI0387	*Pardosa palustris*	Female	Finland	Kilpisjärvi	69.057	20.812	12.7.2015-16.7.2015
UUI0388	*Pardosa palustris*	Female	Finland	Kilpisjärvi	69.057	20.812	12.7.2015-16.7.2015
UUI0389	*Pardosa palustris*	Female	Finland	Kilpisjärvi	69.057	20.812	12.7.2015-16.7.2015
UUI0390	*Pardosa palustris*	Female	Finland	Kilpisjärvi	69.057	20.812	12.7.2015-16.7.2015
UUI0391	*Pardosa palustris*	Female	Finland	Kilpisjärvi	69.057	20.812	12.7.2015-16.7.2015
UUI0392	*Pardosa palustris*	Female	Finland	Kilpisjärvi	69.057	20.812	12.7.2015-16.7.2015
UUI0393	*Pardosa palustris*	Female	Finland	Kilpisjärvi	69.057	20.812	12.7.2015-16.7.2015
UUI0394	*Pardosa palustris*	Female	Finland	Kiiminki	65.147	25.838	8.6.2015-29.6.2015
UUI0395	*Pardosa palustris*	Female	Finland	Kiiminki	65.147	25.838	8.6.2015-29.6.2015
UUI0396	*Pardosa palustris*	Female	Finland	Kiiminki	65.147	25.838	8.6.2015-29.6.2015
UUI0397	*Pardosa palustris*	Female	Finland	Oulu	65.064	25.490	30.6.2015-2.7.2015
UUI0398	*Pardosa palustris*	Female	Finland	Oulu	65.064	25.490	30.6.2015-2.7.2015
UUI0399	*Pardosa palustris*	Female	Finland	Oulu	65.064	25.490	30.6.2015-2.7.2015
UUI0400	*Pardosa palustris*	Female	Finland	Kilpisjärvi	69.057	20.812	12.7.2015-16.7.2015
UUI0401	*Pardosa palustris*	Female	Finland	Kilpisjärvi	69.057	20.812	12.7.2015-16.7.2015
UUI0402	*Pardosa palustris*	Female	Finland	Kilpisjärvi	69.057	20.812	12.7.2015-16.7.2015
UUI0403	*Pardosa palustris*	Female	Finland	Kilpisjärvi	69.057	20.812	12.7.2015-16.7.2015
UUI0404	*Pardosa palustris*	Female	Finland	Kilpisjärvi	69.057	20.812	12.7.2015-16.7.2015
UUI0405	*Pardosa palustris*	Female	Finland	Kiiminki	65.147	25.838	8.6.2015-29.6.2015
UUI0406	*Pardosa palustris*	Female	Finland	Oulu	65.040	25.416	28.6.2015-3.6.2015
UUI0407	*Pardosa palustris*	Female	Russia	Magadan	59.71	151.00	7.7.2017-14.7.2017
UUI0408	*Pardosa palustris*	Female	Russia	Magadan	59.71	151.00	7.7.2017-14.7.2017
UUI0409	*Pardosa palustris*	Female	Russia	Magadan	59.71	151.00	7.7.2017-14.7.2017
UUI0410	*Pardosa palustris*	Female	Russia	Magadan	59.71	151.00	7.7.2017-14.7.2017
UUI0411	*Pardosa palustris*	Female	Russia	Magadan	59.71	151.00	7.7.2017-14.7.2017
UUI0412	*Pardosa palustris*	Female	Russia	Magadan	59.71	151.00	7.7.2017-14.7.2017
UUI0413	*Pardosa palustris*	Female	Russia	Chukotka	64.81	175.97	27.7.2013-9.8.2013
UUI0414	*Pardosa palustris*	Female	Russia	Chukotka	64.81	175.97	27.7.2013-9.8.2013
UUI0415	*Pardosa palustris*	Female	Russia	Chukotka	64.81	175.97	27.7.2013-9.8.2013
UUI0416	*Pardosa palustris*	Female	Russia	Chukotka	64.81	175.97	27.7.2013-9.8.2013
UUI0417	*Pardosa palustris*	Female	Russia	Chukotka	64.81	175.97	27.7.2013-9.8.2013
UUI0418	*Pardosa palustris*	Female	Russia	Magadan	59.71	151.00	7.7.2017-14.7.2017
UUI0419	*Pardosa palustris*	Female	Russia	Magadan	59.71	151.00	7.7.2017-14.7.2017
UUI0420	*Pardosa palustris*	Female	Russia	Magadan	59.71	151.00	7.7.2017-14.7.2017
UUI0421	*Pardosa palustris*	Female	Russia	Chukotka	64.81	175.97	27.7.2013-9.8.2013
UUI0422	*Pardosa palustris*	Female	Russia	Chukotka	64.81	175.97	27.7.2013-9.8.2013
UUI0423	*Pardosa palustris*	Female	Russia	Chukotka	64.81	175.97	27.7.2013-9.8.2013

UUI0424	*Pardosa palustris*	Female	Russia	Chukotka	64.81	175.97	27.7.2013-9.8.2013
UUI0425	*Pardosa palustris*	Female	Russia	Chukotka	64.81	175.97	27.7.2013-9.8.2013
UUI0426	*Pardosa palustris*	Female	Russia	Chukotka	64.81	175.97	27.7.2013-9.8.2013
UUI0427	*Pardosa palustris*	Male	Russia	Magadan	59.567	151.284	7.7.2017-14.7.2017
UUI0428	*Pardosa palustris*	Male	Russia	Magadan	59.567	151.284	7.7.2017-14.7.2017
UUI0429	*Pardosa palustris*	Male	Russia	Magadan	59.567	151.284	7.7.2017-14.7.2017
UUI0430	*Pardosa palustris*	Male	Russia	Magadan	59.567	151.284	7.7.2017-14.7.2017
UUI0431	*Pardosa palustris*	Male	Russia	Magadan	59.567	151.284	7.7.2017-14.7.2017
UUI0432	*Pardosa palustris*	Male	Russia	Magadan	59.567	151.284	7.7.2017-14.7.2017
UUI0433	*Pardosa palustris*	Male	Russia	Magadan	59.567	151.284	7.7.2017-14.7.2017
UUI0434	*Pardosa palustris*	Male	Russia	Magadan	59.567	151.284	7.7.2017-14.7.2017
UUI0435	*Pardosa palustris*	Male	Russia	Magadan	59.567	151.284	7.7.2017-14.7.2017
UUI0436	*Pardosa palustris*	Male	Russia	Magadan	59.567	151.284	7.7.2017-14.7.2017
UUI0437	*Pardosa palustris*	Male	Russia	Chukotka	64.81	175.97	27.7.2013-9.8.2013
UUI0438	*Pardosa palustris*	Male	Russia	Chukotka	64.81	175.97	27.7.2013-9.8.2013
UUI0439	*Pardosa palustris*	Male	Russia	Chukotka	64.81	175.97	27.7.2013-9.8.2013
UUI0440	*Pardosa palustris*	Male	Russia	Chukotka	64.81	175.97	27.7.2013-9.8.2013
UUI0441	*Pardosa palustris*	Male	Russia	Chukotka	64.81	175.97	27.7.2013-9.8.2013
UUI0442	*Pardosa palustris*	Male	Russia	Magadan	64.81	175.97	27.7.2013-9.8.2013
UUI0443	*Pardosa palustris*	Male	Russia	Magadan	59.567	151.284	7.7.2017-14.7.2017
UUI0444	*Pardosa palustris*	Male	Russia	Magadan	59.567	151.284	7.7.2017-14.7.2017
UUI0445	*Pardosa palustris*	Male	Russia	Magadan	59.567	151.284	7.7.2017-14.7.2017
UUI0446	*Pardosa palustris*	Male	Russia	Magadan	59.567	151.284	7.7.2017-14.7.2017

Table S3. Data for specimens used for COI analysis including BOLD records

ID	Species	Gender	Country	Place	Latitude	Longitude	Date
10BGSPI05-G05	*Pardosa agrestis*	M	Bulgaria	Sofiya	42.443	23.58	25-Feb-2010
06-KADIR-D07	*Pardosa agrestis*	F	Bulgaria	Sofiya	42.4431	23.5804	25-Feb-2010
06-KADIR-D10	*Pardosa agrestis*	F	Bulgaria	Sofiya	42.4431	23.5804	25-Feb-2010
AKO_001158	*Pardosa agrestis*	M	Finland		60.13	22.182	18-Jun-2014
AKO_001159	*Pardosa agrestis*	F	Finland		60.13	22.182	18-Jun-2014
AKO_001252	*Pardosa agrestis*	M	Finland		60.367	23.11	18-Jun-2014
AKO_001253	*Pardosa agrestis*	F	Finland		60.367	23.11	18-Jun-2014
ZFMK-TIS-18325	*Pardosa agrestis*	M	Germany	Mecklenburg-Vorpommern	54.47	13.21	07-May-2013
ZFMK-TIS-18353	*Pardosa agrestis*	M	Germany	Mecklenburg-Vorpommern	54.68	13.38	13-May-2013
ZFMK-TIS-22258	*Pardosa agrestis*	M	Germany	Mecklenburg-Vorpommern	54.47	13.21	07-May-2013
ZFMK-TIS-22270	*Pardosa agrestis*	M	Germany	Mecklenburg-Vorpommern	54.68	13.38	13-May-2013
ZFMK-DNA-100424408	*Pardosa agrestis*	M	Germany	Baden-Wuerttemberg	48.97	8.48	
ZFMK-DNA-100415466	*Pardosa agrestis*	F	Germany	Baden-Wuerttemberg	48.81	8.19	
ZFMK-TIS-19077	*Pardosa agrestis*	M	Germany	Schleswig-Holstein	54.47	10.02	05-Jun-2013
ZFMK-TIS-2436	*Pardosa agrestis*	M	Germany	Mecklenburg-Vorpommern	54.32	13.46	24-May-2012
01-KADIR-A03	*Pardosa agrestis*	F	Turkey	Van	38.5046	43.4546	22-Aug-2009
01-KADIR-A06	*Pardosa agrestis*	F	Turkey	Van	38.4828	43.4147	22-Sep-2009
01-KADIR-A07	*Pardosa agrestis*	F	Turkey	Van	38.4828	43.4147	22-Sep-2009
BIOUG00522-E07	*Pardosa agrestis*		Turkey	Erzincan	39.8794	39.3425	25-May-2010
ZMUO.002753	*Pardosa agricola*	M	Finland	Ostrobothnia	63.2288	20.6679	19-Jun-2007
ZMUO.002754	*Pardosa agricola*	F	Finland	Ostrobothnia	63.2288	20.6679	19-Jun-2007
ZMUO.002650	*Pardosa agricola*	M	Finland	Uusimaa	59.825	23.0296	26-Jun-2010
ZMUO.002652	*Pardosa agricola*	F	Finland	Northern Ostrobothnia	66.2912	29.6297	15-Jun-2011
ZMUO.002653	*Pardosa agricola*	M	Finland	Northern Ostrobothnia	66.2912	29.6297	15-Jun-2011
AKO_001150	*Pardosa agricola*	F	Finland		60.13	22.182	18-Jun-2014
AKO_001151	*Pardosa agricola*	F	Finland		60.13	22.182	18-Jun-2014
AKO_001154	*Pardosa agricola*	M	Finland		60.13	22.182	18-Jun-2014
AKO_001155	*Pardosa agricola*	M	Finland		60.13	22.182	18-Jun-2014
AKO_001157	*Pardosa agricola*	F	Finland		60.13	22.182	18-Jun-2014
AKO_001156	*Pardosa agricola*	M	Finland		60.13	22.182	18-Jun-2014
UUI0074	*Pardosa agricola*	F	Finland		66.2906	29.6293	06-Jul-2015
BIOUG00160-A05	*Pardosa albatula*	F	Bulgaria		41.7877	23.4065	08-Aug-2010
BIOUG00526-A08	*Pardosa albatula*	M	Turkey	Kastamonu	41.6357	33.1447	17-May-2009
ZFMK-TIS-19223	*Pardosa blanda*	M	Switzerland	Valais	46.08	7.61	08-Jun-2012
ZFMK-TIS-2649	*Pardosa blanda*	M	Switzerland	Valais	46.08	7.61	08-Jun-2012
ZFMK-TIS-2654	*Pardosa mixta*	F	Switzerland	Valais	46.25	8.05	08-Jul-2012
ZFMK-TIS-2682	*Pardosa mixta*	F	Switzerland	Valais	46.39	8.18	20-Aug-2014
ZFMK-TIS-2524839	*Pardosa monticola*	F	Germany	Schleswig-Holstein	54.34	9.52	18-Jun-2014
ZFMK-TIS-2534450	*Pardosa monticola*	F	Germany	Schleswig-Holstein	53.48	10.74	10-Mar-2015

ZFMK-TIS-2534451	*Pardosa monticola*	F	Germany	Schleswig-Holstein	53.48	10.74	09-Feb-2015
ZFMK-TIS-19079	*Pardosa monticola*	M	Germany	Schleswig-Holstein	54.47	10.02	05-Jun-2013
MAR_6	*Pardosa ovtchinnikovi*	F	Tajikistan		37.153	68.071	20-Apr-2015
MAR_7	*Pardosa ovtchinnikovi*	F	Tajikistan		37.153	68.071	20-Apr-2015
MAR_8	*Pardosa ovtchinnikovi*	F	Tajikistan		37.153	68.071	20-Apr-2015
MAR_9	*Pardosa ovtchinnikovi*	M	Tajikistan		37.153	68.071	20-Apr-2015
JPF_10	*Pardosa palustris*	F	Faeroe Islands		62.02	-6.77	13-Jun-2012
JPF_12	*Pardosa palustris*	F	Faeroe Islands		62.02	-6.77	13-Jun-2012
AKO_001264	*Pardosa palustris*	F	Finland		66.412	26.182	08-Jul-2011
AKO_001265	*Pardosa palustris*	F	Finland		66.412	26.182	08-Jul-2011
AKO_001266	*Pardosa palustris*	F	Finland		66.412	26.182	08-Jul-2011
AKO_001872	*Pardosa palustris*	F	Finland		69.082	21.538	25-Jul-2013
UUI0105	*Pardosa palustris*	M	Finland		69.0818	20.7505	12-Jul-2015
UUI0107	*Pardosa palustris*	M	Finland		65.0403	25.416	28-May-2015
UUI0108	*Pardosa palustris*	M	Finland		65.1478	25.8383	15-Jun-2015
UUI0110	*Pardosa palustris*	M	Finland		65.0644	25.4904	02-Jul-2015
ZMUO.000097	*Pardosa palustris*	F	Finland	Northern Ostrobothnia	65.137	25.887	31-Jul-2011
UUI0070	*Pardosa palustris*	M	Finland		69.0562	20.797	14-Jul-2015
BC ZSM ARA 00465	*Pardosa palustris*	M	Germany	Saxony-Anhalt	51.5016	11.8061	26-May-2011
ZSM_B_ARACH_717	*Pardosa palustris*	M	Germany	Brandenburg	52.03	13.281	19-May-2012
ZFMK-TIS-18355	*Pardosa palustris*	F	Germany	Mecklenburg-Vorpommern	54.32	13.45	15-May-2013
ZFMK-TIS-18398	*Pardosa palustris*	M	Germany	Saxony	51.58	12.7	03-Jun-2013
ZFMK-TIS-22271	*Pardosa palustris*	M	Germany	Mecklenburg-Vorpommern	54.32	13.45	15-May-2013
ZFMK-DNA-100415509	*Pardosa palustris*	F	Germany	Baden-Wuerttemberg	49.06	11.86	
ZFMK-DNA-100415535	*Pardosa palustris*	M	Germany	Baden-Wuerttemberg	48.82	8.84	
ZFMK-DNA-100415537	*Pardosa palustris*	M	Germany	Baden-Wuerttemberg	48.82	8.84	
ZFMK-DNA-100415538	*Pardosa palustris*	M	Germany	Baden-Wuerttemberg	48.82	8.84	
ZFMK-DNA-100415548	*Pardosa palustris*	M	Germany	Baden-Wuerttemberg	48.82	8.84	
ZFMK-DNA-100415551	*Pardosa palustris*	M	Germany	Baden-Wuerttemberg	48.82	8.84	
ZFMK-DNA-100415558	*Pardosa palustris*	F	Germany	Baden-Wuerttemberg	48.82	8.84	
ZFMK-DNA-100415956	*Pardosa palustris*	M	Germany	Baden-Wuerttemberg	48.82	8.84	
ZFMK-DNA-100415976	*Pardosa palustris*	M	Germany	Baden-Wuerttemberg	48.82	8.84	
ZFMK-DNA-100415981	*Pardosa palustris*	M	Germany	Baden-Wuerttemberg	48.82	8.84	
ZFMK-DNA-100416001	*Pardosa palustris*	M	Germany	Baden-Wuerttemberg	48.82	8.84	
ZFMK-DNA-100416026	*Pardosa palustris*	M	Germany	Baden-Wuerttemberg	48.82	8.84	
ZFMK-DNA-100416338	*Pardosa palustris*	M	Germany	Baden-Wuerttemberg	48.82	8.84	
ZFMK-DNA-100416387	*Pardosa palustris*	M	Germany	Baden-Wuerttemberg	48.82	8.84	

ZFMK-TIS-2479	*Pardosa palustris*	F	Germany	Saxony	51.18	13.6	27-Jun-2012
NHMO_ARA00254	*Pardosa palustris*	M	Norway	Oslo			02-Jun-2009
NHMO_ARA00256	*Pardosa palustris*	M	Norway	Oslo			02-Jun-2009
NHMO_ARA00257	*Pardosa palustris*	M	Norway	Oslo			02-Jun-2009
NHMO_ARA00258	*Pardosa palustris*	M	Norway	Oslo			02-Jun-2009
MAR_12	*Pardosa palustris*	F	Russia		59.745	150.848	23-Jun-2015
MAR_13	*Pardosa palustris*	M	Russia		59.745	150.848	23-Jun-2015
MAR_15	*Pardosa palustris*	M	Russia		59.745	150.848	23-Jun-2015
BIOUG01895-E03	*Pardosa palustris*	F	Russia	Kamchatskiy Kray	62.454	166.191	31-Jul-2011
BIOUG00606-C04	*Pardosa palustris*	F	Russia	Magadanskaya Oblast	59.7352	150.867	06-Oct-2010
BIOUG00606-C05	*Pardosa palustris*		Russia	Magadanskaya Oblast	59.7352	150.867	06-Oct-2010
BIOUG00882-E06	*Pardosa palustris*		Russia	Kamchatskiy Kray	59.087	159.942	09-Aug-2009
UAM:Ento:94549	*Pardosa palustris*		United States	Alaska	51.979	-178.499	09-Jun-2008
UAM:Ento:87619	*Pardosa palustris*		United States	Alaska	52.169	-175.525	11-Jun-2008
MZH130930	*Pardosa plumipes*		Finland		64.414	24.05	06-Jun-2012
MZH122018	*Pardosa plumipes*	F	Finland		64.413	24.051	16-Jul-2012
MZH130929	*Pardosa plumipes*		Finland		64.414	24.05	30-Jun-2012
ZMH122148	*Pardosa plumipes*	F	Finland		64.4135	24.0506	08-Jul-2012
BIOUG00887-H02	*Pardosa prativaga*	F	Bulgaria		42.9344	22.9544	22-May-2011
UUI0052	*Pardosa prativaga*	M	Finland		59.8433	23.212	11-Jun-2015
UUI0054	*Pardosa prativaga*	M	Finland		59.8433	23.212	11-Jun-2015
UUI0055	*Pardosa prativaga*	F	Finland		66.3777	29.3077	06-Aug-2015
UUI0057	*Pardosa prativaga*	F	Finland		59.8296	23.1425	11-Jun-2015
UUI0058	*Pardosa prativaga*	F	Finland		59.8296	23.1425	11-Jun-2015
UUI0062	*Pardosa prativaga*	F	Finland		59.8433	23.212	08-Jun-2015
BC ZSM ARA 00348	*Pardosa prativaga*	F	Germany	Saxony-Anhalt	52.2967	11.6922	07-May-2011
BC ZSM ARA 00349	*Pardosa prativaga*	F	Germany	Saxony-Anhalt	52.2967	11.6922	07-May-2011
ZFMK-TIS-18362	*Pardosa prativaga*	M	Germany	Mecklenburg-Vorpommern	54.1	13.75	28-May-2013
ZFMK-TIS-18397	*Pardosa prativaga*	M	Germany	Saxony	51.58	12.7	03-Jun-2013
ZFMK-TIS-2524807	*Pardosa prativaga*	F	Germany	Schleswig-Holstein	53.66	10.87	
ZFMK-TIS-2524827	*Pardosa prativaga*	F	Germany	Schleswig-Holstein	53.81	10.75	02-Jun-2014
ZFMK-DNA-100415514	*Pardosa prativaga*	F	Germany	Baden-Wuerttemberg	49.06	11.86	
ZFMK-TIS-19066	*Pardosa prativaga*	F	Germany	Schleswig-Holstein	54.45	9.86	05-Jun-2013
ZFMK-TIS-2355	*Pardosa prativaga*	M	Germany	Mecklenburg-Vorpommern	54.34	13.45	25-May-2012
ZFMK-TIS-2477	*Pardosa prativaga*	F	Germany	Saxony	51.18	13.6	27-Jun-2012
SMNK-ARA 09007	*Pardosa prativaga*	M	Germany	Baden-Wuerttemberg			
SMNK-ARA 09051	*Pardosa prativaga*	M	Germany	Baden-Wuerttemberg			
SMNK-ARA 09060	*Pardosa prativaga*	M	Germany	Baden-Wuerttemberg			
SMNK-ARA 09070	*Pardosa prativaga*	F	Germany	Baden-Wuerttemberg			
ZFMK-TIS-2528501	*Pardosa pullata*	F	Austria		47.29	11.38	04-Jun-2014
ZMUO.002706	*Pardosa pullata*	F	Finland	Aland Islands	60.1686	19.5605	01-Jun-2011
MZH45971	*Pardosa pullata*		Finland		60.285	25.161	11-May-2006
AKO_001686	*Pardosa pullata*	F	Finland		65.095	25.963	23-Jul-2012

AKO_001775	*Pardosa pullata*	F	Finland		60.367	23.11	25-May-2012
UUI0027	*Pardosa pullata*	F	Finland		59.8433	23.212	11-Jun-2015
UUI0028	*Pardosa pullata*	F	Finland		59.8433	23.212	11-Jun-2015
UUI0029	*Pardosa pullata*	M	Finland		59.8433	23.212	11-Jun-2015
UUI0030	*Pardosa pullata*	M	Finland		59.8433	23.212	11-Jun-2015
UUI0056	*Pardosa pullata*	M	Finland		59.8433	23.212	11-Jun-2015
UUI0060	*Pardosa pullata*	M	Finland		59.8433	23.212	08-Jun-2015
ARACHNO-0046	*Pardosa pullata*	M	France		49.4592	1.0775	01-Apr-2009
11239-A07	*Pardosa pullata*		France		49.4588	1.07787	09-May-2011
11239-A08	*Pardosa pullata*		France		49.4588	1.07787	09-May-2011
11239-G06	*Pardosa pullata*		France		49.4588	1.07787	16-May-2011
11239-G07	*Pardosa pullata*		France		49.4588	1.07787	16-May-2011
11239-H01	*Pardosa pullata*		France		49.4588	1.07787	16-May-2011
11243-G12	*Pardosa pullata*		France	Normandie	49.7112	1.28811	27-May-2011
11235-G01	*Pardosa pullata*		France	Normandie	49.459	1.078	23-May-2011
01336-A02	*Pardosa pullata*		France	Normandie	49.459	1.078	26-May-2011
01336-A03	*Pardosa pullata*		France	Normandie	49.459	1.078	26-May-2011
ARACHNO-0057	*Pardosa pullata*	M	France		49.4592	1.0775	01-Apr-2009
ARACHNO-0066	*Pardosa pullata*	F	France		49.4592	1.0775	01-Apr-2009
ZFMK-TIS-18326	*Pardosa pullata*	F	Germany	Mecklenburg-Vorpommern	54.47	13.21	07-May-2013
ZFMK-TIS-2505011	*Pardosa pullata*	F	Germany	Lower Saxony	53.71	8.8	15-May-2014
ZFMK-TIS-2517366	*Pardosa pullata*	F	Germany	Rhineland-Palatinate	49.89	6.77	
ZFMK-TIS-2523256	*Pardosa pullata*	F	Germany	Rhineland-Palatinate	49.89	6.79	
ZFMK-DNA-100415510	*Pardosa pullata*	F	Germany	Baden-Wuerttemberg	49.06	11.86	
ZFMK-DNA-100415513	*Pardosa pullata*	F	Germany	Baden-Wuerttemberg	49.06	11.86	
ZFMK-DNA-100415515	*Pardosa pullata*	F	Germany	Baden-Wuerttemberg	49.06	11.86	
ZFMK-DNA-100415516	*Pardosa pullata*	F	Germany	Baden-Wuerttemberg	49.06	11.86	
ZFMK-DNA-100415519	*Pardosa pullata*	F	Germany	Baden-Wuerttemberg	49.06	11.86	
ZFMK-DNA-100415520	*Pardosa pullata*	F	Germany	Baden-Wuerttemberg	49.06	11.86	
ZFMK-DNA-100415521	*Pardosa pullata*	F	Germany	Baden-Wuerttemberg	49.06	11.86	
ZFMK-DNA-100415526	*Pardosa pullata*	F	Germany	Baden-Wuerttemberg	48.82	8.84	
ZFMK-DNA-100415527	*Pardosa pullata*	M	Germany	Baden-Wuerttemberg	48.82	8.84	
ZFMK-DNA-100416028	*Pardosa pullata*	F	Germany	Baden-Wuerttemberg	49.06	11.86	
ZFMK-DNA-100424397	*Pardosa pullata*	M	Germany	Baden-Wuerttemberg	48.97	8.48	
ZFMK-TIS-19330	*Pardosa pullata*	F	Germany	Saxony	50.504	13.19	18-Sep-2012
ZFMK-TIS-2699	*Pardosa pullata*	F	Germany	Saxony	50.504	13.19	18-Sep-2012
ZFMK-TIS-2825	*Pardosa pullata*	M	Germany	Mecklenburg-Vorpommern	54.28	13.4	01-May-2013
ZFMK-TIS-7208	*Pardosa pullata*	F	Germany	Schleswig-Holstein	53.92	10.01	

SMNK-ARA 08393	*Pardosa pullata*	M	Germany	Baden-Wuerttemberg			
SMNS-Aran-1091	*Pardosa pullata*	F	Germany		48.73	9.95	01-Jan-2014
NHMO_ARA00226	*Pardosa pullata*	M	Norway	Oslo			02-Jun-2009
NHMO_ARA00252	*Pardosa pullata*	M	Norway	Oslo			02-Jun-2009
Lygra06	*Pardosa pullata*		Norway	Hordaland	60.637	5.214	18-Jun-2016
ZFMK-TIS-15922	*Pardosa riparia*	F	Austria		47.09	12.73	03-Oct-2012
MZH26891	*Pardosa riparia*	M	Finland		66.028	24.958	01-Jun-2001
MZH12624	*Pardosa riparia*	M	Finland		65.836	25.251	14-Jun-2000
MZH12176	*Pardosa riparia*	M	Finland		65.836	25.251	14-Jun-2000
MZH26708	*Pardosa riparia*	M	Finland		66.028	24.958	15-Jun-2001
AKO_001245	*Pardosa riparia*	M	Finland		60.367	23.11	18-Jun-2014
ZMUO.000170	*Pardosa riparia*	F	Finland	Northern Ostrobothnia	65.137	25.887	31-Jul-2011
ZMUO.000171	*Pardosa riparia*	F	Finland	Northern Ostrobothnia	65.137	25.887	31-Jul-2011
ZMUO.000053	*Pardosa riparia*	F	Finland	North Karelia	62.6075	31.101	07-Aug-2011
UUI0084	*Pardosa riparia*	M	Finland		65.1478	25.8383	19-Jun-2015
UUI0085	*Pardosa riparia*	M	Finland		65.1478	25.8383	19-Jun-2015
UUI0086	*Pardosa riparia*	F	Finland		65.1478	25.8383	31-Jul-2011
UUI0087	*Pardosa riparia*	F	Finland		65.1478	25.8383	26-Jun-2015
UUI0088	*Pardosa riparia*	F	Finland		65.1478	25.8383	26-Jun-2015
UUI0089	*Pardosa riparia*	M	Finland		65.1478	25.8383	19-Jun-2015
UUI0090	*Pardosa riparia*	F	Finland		65.1478	25.8383	19-Jun-2015
SMNS-Aran-875	*Pardosa riparia*	M	Germany		48.41	9.85	01-Jan-2013
MAR_18	*Pardosa riparia*	M	Russia		59.745	150.848	23-Jun-2015
MAR_19	*Pardosa riparia*	F	Russia		59.745	150.848	23-Jun-2015
MAR_20	*Pardosa riparia*	M	Russia		59.745	150.848	23-Jun-2015
ZFMK-TIS-2677	*Pardosa riparia*	M	Switzerland	Valais	46.39	8.18	08-Aug-2012
MZH130931	*Pardosa sphagnicola*		Finland		66.858	27.555	02-Jun-2000
MZH130932	*Pardosa sphagnicola*		Finland		67.397	26.727	04-Jun-2008
MZH42977	*Pardosa sphagnicola*	M	Finland		66.859	27.556	02-Apr-2004
ZMUO.000172	*Pardosa sphagnicola*	F	Finland	Northern Ostrobothnia	65.237	26.232	02-Aug-2011
ZMUO.000173	*Pardosa sphagnicola*	F	Finland	Northern Ostrobothnia	65.237	26.232	02-Aug-2011
ZMUO.000174	*Pardosa sphagnicola*	F	Finland	Northern Ostrobothnia	65.237	26.232	02-Aug-2011
ZMUO.000043	*Pardosa sphagnicola*	M	Finland	Northern Ostrobothnia	65.328	25.7296	14-May-2011
ZMUO.000045	*Pardosa sphagnicola*	M	Finland	Northern Ostrobothnia	65.0902	25.9701	28-May-2011
ZMUO.000046	*Pardosa sphagnicola*	M	Finland	Northern Ostrobothnia	65.2343	25.8378	28-May-2011
ZMUO.000047	*Pardosa sphagnicola*	M	Finland	Northern Ostrobothnia	65.0902	25.9701	28-May-2011
ZMUO.000048	*Pardosa sphagnicola*	M	Finland	Northern Ostrobothnia	65.3281	25.7318	24-May-2011
ZMUO.000049	*Pardosa sphagnicola*	F	Finland	North Karelia	62.6075	31.101	07-Aug-2011
ZMUO.000050	*Pardosa sphagnicola*	F	Finland	North Karelia	62.6075	31.101	07-Aug-2011
ZMUO.000051	*Pardosa sphagnicola*	F	Finland	North Karelia	62.6075	31.101	07-Aug-2011
ZMUO.000052	*Pardosa sphagnicola*	F	Finland	North Karelia	62.6075	31.101	07-Aug-2011
ZMUO.000055	*Pardosa sphagnicola*	F	Finland	North Karelia	62.6075	31.101	07-Aug-2011
ZMUO.000056	*Pardosa sphagnicola*	F	Finland	North Karelia	62.6075	31.101	07-Aug-2011

ZMUO.000057	*Pardosa sphagnicola*	F	Finland	Northern Ostrobothnia	65.9961	29.1427	22-Aug-2011
ZMUO.000058	*Pardosa sphagnicola*	F	Finland	Northern Ostrobothnia	65.2343	25.8378	04-Aug-2011
ZMUO.000059	*Pardosa sphagnicola*	F	Finland	Northern Ostrobothnia	65.2367	26.2317	02-Aug-2011
ZMUO.000060	*Pardosa sphagnicola*	F	Finland	Northern Ostrobothnia	65.2343	25.8378	21-Jul-2011
UUI0025	*Pardosa sphagnicola*	F	Finland		65.1478	25.8383	15-Jun-2015
UUI0041	*Pardosa sphagnicola*	M	Finland		65.1478	25.8383	19-Jun-2015
UUI0042	*Pardosa sphagnicola*	M	Finland		65.1478	25.8383	02-Jul-2015
UUI0043	*Pardosa sphagnicola*	M	Finland		66.3777	29.3077	09-Jun-2015
UUI0044	*Pardosa sphagnicola*	F	Finland		59.8433	23.212	11-Jun-2015
UUI0046	*Pardosa sphagnicola*	M	Finland		65.1478	25.8383	19-Jun-2015
UUI0050	*Pardosa sphagnicola*	M	Finland		65.0644	25.4904	01-Jul-2015
GBOL08323	*Pardosa torrentum*	M	Germany	Bavaria	47.727	11.566	28-May-2010

Table S4. *Pardosa riparia* measurements in microscope scale units.

ID	Species	Gender	Country	CarLen	CarWid	PatTib	CymbLen	CymbTip	BulbLen	BulbWid	ApophLen	SeptLen	SeptWid
UUI0307	*Pardosa riparia*	Male	Finland	16.1	11.4	15.5	6.9	2.2	3.5	2.8	4.2		
UUI0308	*Pardosa riparia*	Male	Finland	16.6	11.8	14.9	6.9	2.1	3.9	3.2	3.9		
UUI0309	*Pardosa riparia*	Male	Finland	17.5	12.5	14.9	6.8	2.8	4.3	3.1	4.3		
UUI0310	*Pardosa riparia*	Male	Finland	17.4	12.6	14.6	6.5	2.2	4	3.6	3.8		
UUI0311	*Pardosa riparia*	Male	Finland	18.2	12.6	15.5	6.8	2.4	4.3	3.6	3.9		
UUI0312	*Pardosa riparia*	Male	Finland	15.8	11.3	14.6	6.7	2.1	4	3.4	3.9		
UUI0313	*Pardosa riparia*	Male	Finland	16	11.6	15.8	6.7	2.4	4.3	3.2	4.3		
UUI0314	*Pardosa riparia*	Male	Finland	18.1	12.4	16.1	7.1	2.4	3.8	3.5	4.2		
UUI0315	*Pardosa riparia*	Male	Finland	15.4	11.1	14.2	6.1	1.8	3.8	3.2	3.9		
UUI0316	*Pardosa riparia*	Male	Finland	17	11.7	14.9	6.5	2.5	3.6	3.5			
UUI0317	*Pardosa riparia*	Male	Finland	16.5	11.2	14.7	6.8	2.3	4.2	3.3	4.2		
UUI0318	*Pardosa riparia*	Male	Finland	17.2	11.6	15.3	7	2.2	3.8	3.7	4.9		
UUI0319	*Pardosa riparia*	Male	Finland	16.5	12	15	6.5	2.2	3.8	3.4	4.1		
UUI0320	*Pardosa riparia*	Male	Finland	16.8	11.6	14.2	6.9	2.3	4.1	3.5	4.1		
UUI0321	*Pardosa riparia*	Male	Finland	15.2	11.5	15.5	7.1	2.5	4.4	3.6	4.5		
UUI0322	*Pardosa riparia*	Female	Finland	16.1	12.3	15.8						2.8	1.9
UUI0323	*Pardosa riparia*	Female	Finland	18	12	15.8						2.6	2
UUI0324	*Pardosa riparia*	Female	Finland	15.5	12.3	14.5						3	2.4
UUI0325	*Pardosa riparia*	Female	Finland	16	12.5	14						2.5	2.2
UUI0326	*Pardosa riparia*	Female	Finland	16.7	13.5	16.2						3.9	2.5
UUI0327	*Pardosa riparia*	Female	Finland	17	12.4	15.6						3	1.9
UUI0328	*Pardosa riparia*	Female	Finland	17.4	12.5	15.5						2.9	1.8
UUI0329	*Pardosa riparia*	Female	Finland	18	13.9	16.5						3.2	1.8
UUI0330	*Pardosa riparia*	Female	Finland	17.5	13	15.8						3.2	2.5
UUI0331	*Pardosa riparia*	Female	Finland	17.2	12.8	14.6						3.3	1.9
UUI0332	*Pardosa riparia*	Female	Finland	17.5	12.6	15.5						3.5	2
UUI0333	*Pardosa riparia*	Female	Finland	15.5	12	14.2						3.4	2.2
UUI0334	*Pardosa riparia*	Female	Finland	17	13	15						3	2

UUI0335	*Pardosa riparia*	Female	Finland	16.5	13	15.4						3	2.1
UUI0336	*Pardosa riparia*	Female	Finland	18.1	12.6	14.5						3.1	2.1
UUI0337	*Pardosa riparia*	Male	Russia	17.4	12	17.9	7	1.8	4.7	3.4	3.7		
UUI0338	*Pardosa riparia*	Male	Russia	16	12.8	17.5	6.9	2.3	4.2	4.1	3.6		
UUI0339	*Pardosa riparia*	Male	Russia	16.1	11	16.5	6.4	1.7	4	3.4	3.9		
UUI0340	*Pardosa riparia*	Male	Russia	16.5	12	17	7	2.4	4.3	3.3	4.1		
UUI0341	*Pardosa riparia*	Male	Russia	17.8	12.2	17.5	7.2	2.5	4.5	3.7	4.3		
UUI0342	*Pardosa riparia*	Male	Russia	16	12	12.7	6.5	2.4	4.2	3.6	4.2		
UUI0343	*Pardosa riparia*	Male	Russia	17	14	18	7.5	2.8	4.5	3.7	4.6		
UUI0344	*Pardosa riparia*	Male	Russia	17.1	13	17.5	7.4	2.2	4.7	3.7	4.6		
UUI0345	*Pardosa riparia*	Male	Russia	17.8	12.4	17.9	7.1	2.1	3.9	3.6	3.7		
UUI0346	*Pardosa riparia*	Male	Russia	19.5	14	17.9	7.4	2.5	4.3	3.9	4.2		
UUI0347	*Pardosa riparia*	Male	Russia	16	11.2	16.9	7	2	4.3	3.5	4.4		
UUI0348	*Pardosa riparia*	Male	Russia	17	12.5	18.2	7.7	2.8	4.1	3.7	4.5		
UUI0349	*Pardosa riparia*	Male	Russia	17.1	13.1	18.1	7.6	2.5	4.5	3.8	4.3		
UUI0350	*Pardosa riparia*	Male	Russia	17.8	12.5	18	7.2	2.3	4	3.6	4.1		
UUI0351	*Pardosa riparia*	Male	Russia	18	13	18.2	8	2.8	4.4	3.8	4.3		
UUI0352	*Pardosa riparia*	Female	Russia	18.2	14.5	17.5						3.5	2
UUI0353	*Pardosa riparia*	Female	Russia	16	15	18.1						3.2	1.9
UUI0354	*Pardosa riparia*	Female	Russia	18	14	19						2.8	1.8
UUI0355	*Pardosa riparia*	Female	Russia	18	16	18.9						3.4	2
UUI0356	*Pardosa riparia*	Female	Russia	18.2	14.5	17.5						3.5	2.5
UUI0357	*Pardosa riparia*	Female	Russia	20	15	19						4	2.4
UUI0358	*Pardosa riparia*	Female	Russia	19	14	18.3						3.8	1.6
UUI0359	*Pardosa riparia*	Female	Russia	18	15	18						4	2
UUI0361	*Pardosa riparia*	Female	Russia	17.6	15.2	19.7						4	1.5
UUI0362	*Pardosa riparia*	Female	Russia	18.2	15	17.5						3.6	1.8
UUI0363	*Pardosa riparia*	Female	Russia	18	14	18						3.4	1.4
UUI0364	*Pardosa riparia*	Female	Russia	18.5	14.5	19						3.2	2
UUI0365	*Pardosa riparia*	Female	Russia	19.7	14.7	18.5						3.5	2.2
UUI0366	*Pardosa riparia*	Female	Russia	19.4	15	19.5						3.2	2.1

Table S5. *Pardosa palustris* measurements in microscope scale units.

ID	Species	Gender	Country	Place	CarLen	CarWid	PatTib	CymbLen	CymbTip	BulbLen	BulbWid	SeptLen	SeptWid
UUI0367	*Pardosa palustris*	Male	Finland	Kilpisjärvi	12	9	11	4.8	1	2.9	3.2		
UUI0368	*Pardosa palustris*	Male	Finland	Kilpisjärvi	11.8	8.6	11.5	5	1.1	3.3	3.1		
UUI0369	*Pardosa palustris*	Male	Finland	Kilpisjärvi	12	8.8	11.7	5.2	1.2	3.3	3.1		
UUI0370	*Pardosa palustris*	Male	Finland	Kilpisjärvi	11.5	8.3	11.1	4.9	1.1	3.1	3		
UUI0371	*Pardosa palustris*	Male	Finland	Kilpisjärvi	11.4	8.8	11.4	5.2	1.2	3	3		
UUI0372	*Pardosa palustris*	Male	Finland	Kilpisjärvi	11.8	9	11.8	5.2	1.2	3.2	3.1		
UUI0373	*Pardosa palustris*	Male	Finland	Kilpisjärvi	11.7	9	11.7	5.1	1.1	3	3		
UUI0374	*Pardosa palustris*	Male	Finland	Kiiminki	11	8.2	11.2	4.8	1	2.8	2.7		
UUI0375	*Pardosa palustris*	Male	Finland	Kiiminki	11	7.9	11.1	5	1.1	3.1	3		
UUI0376	*Pardosa palustris*	Male	Finland	Kiiminki	10.8	8.2	10.7	5.1	1.1	3.2	3.1		
UUI0377	*Pardosa palustris*	Male	Finland	Kiiminki	10.6	7.7	10.9	5	1.1	3.1	3		
UUI0378	*Pardosa palustris*	Male	Finland	Kiiminki	10.2	7.7	11	4.8	0.9	3	3		
UUI0379	*Pardosa palustris*	Male	Finland	Kiiminki	10.6	8	11	4.9	1.2	2.9	3		
UUI0380	*Pardosa palustris*	Male	Finland	Kiiminki	10.3	7.9	11	4.6	1.1	3	2.8		
UUI0381	*Pardosa palustris*	Male	Finland	Oulu	11.5	8.8	11	5.2	1.3	3.3	3		
UUI0382	*Pardosa palustris*	Male	Finland	Oulu	11.5	8.6	11.8	5	1.1	3.2	3.1		
UUI0383	*Pardosa palustris*	Male	Finland	Oulu	12.1	9.2	12	5.3	1.3	3.4	2.9		
UUI0384	*Pardosa palustris*	Male	Finland	Oulu	11	8.5	11.7	5.1	1	3.3	3.1		
UUI0385	*Pardosa palustris*	Male	Finland	Oulu	11.2	8.7	11.1	5	1.1	3.1	3		
UUI0386	*Pardosa palustris*	Male	Finland	Oulu	11.5	9	11.7	5	1.2	3.1	3		
UUI0111	*Pardosa palustris*	Male	Finland	Kiiminki	10	8.2	10.7	4.8	1.2	2.8	2.8		
UUI0110	*Pardosa palustris*	Male	Finland	Oulu	11.3	8.5	11.2	5	1.1	3.1	2.9		
UUI0105	*Pardosa palustris*	Male	Finland	Kilpisjärvi	10.8	8.5	11.4	4.8	1.2	2.9	2.9		
UUI0107	*Pardosa palustris*	Male	Finland	Oulu	10.8	8.2	11	5.1	1.3	3	2.9		
UUI0108	*Pardosa palustris*	Male	Finland	Kiiminki	10.8	8.3	11.4	4.8	1	2.9	2.7		
UUI0109	*Pardosa palustris*	Male	Finland	Kiiminki	10.8	8	10.9	4.7	1	3	2.8		
UUI0106	*Pardosa palustris*	Female	Finland	Kilpisjärvi	10.6	8.2	10.7					2.3	3.2
UUI0387	*Pardosa palustris*	Female	Finland	Kilpisjärvi	10.5	8.5	10					2.3	2.8

UUI0388	*Pardosa palustris*	Female	Finland	Kilpisjärvi	10.6	8.5	10.1					2.4	2.9
UUI0389	*Pardosa palustris*	Female	Finland	Kilpisjärvi	10.7	9	10					2.3	3
UUI0390	*Pardosa palustris*	Female	Finland	Kilpisjärvi	10.5	8.7	10.5					2.4	2.9
UUI0391	*Pardosa palustris*	Female	Finland	Kilpisjärvi	11.1	8.8	10.3					2.5	3
UUI0392	*Pardosa palustris*	Female	Finland	Kilpisjärvi	10.5	8.8	11					2.3	3
UUI0393	*Pardosa palustris*	Female	Finland	Kilpisjärvi	10.3	8.5	10					2.3	2.9
UUI0394	*Pardosa palustris*	Female	Finland	Kiiminki	10.9	8.6	11					2.4	3.2
UUI0395	*Pardosa palustris*	Female	Finland	Kiiminki	10.5	8.5	10.2					2.3	3.3
UUI0396	*Pardosa palustris*	Female	Finland	Kiiminki	10.5	8	9.5					2.3	3
UUI0397	*Pardosa palustris*	Female	Finland	Oulu	10	8.3	10					2.3	3
UUI0398	*Pardosa palustris*	Female	Finland	Oulu	11.3	9.3	11.1					2.4	3.4
UUI0399	*Pardosa palustris*	Female	Finland	Oulu	11	8.3	10.8					2.4	3.2
UUI0400	*Pardosa palustris*	Female	Finland	Kilpisjärvi	11	9	10.5					2.3	3
UUI0401	*Pardosa palustris*	Female	Finland	Kilpisjärvi	10.5	8.2	10.3					2.3	2.9
UUI0402	*Pardosa palustris*	Female	Finland	Kilpisjärvi	10.7	8.5	10.2					2.2	3.2
UUI0403	*Pardosa palustris*	Female	Finland	Kilpisjärvi	12.2	9.6	11.5					2.4	3.2
UUI0404	*Pardosa palustris*	Female	Finland	Kilpisjärvi	11.4	8.6	11.2					2.3	2.9
UUI0405	*Pardosa palustris*	Female	Finland	Kiiminki	10.5	7.8	9.5					2.1	3
UUI0406	*Pardosa palustris*	Female	Finland	Oulu	12	9	11					2.4	3.2
UUI0407	*Pardosa palustris*	Female	Russia	Magadan	11.5	9	12					2.5	3.1
UUI0408	*Pardosa palustris*	Female	Russia	Magadan	11.5	9.6	11.8					2.5	3
UUI0409	*Pardosa palustris*	Female	Russia	Magadan	11.8	9.5	11.9					2.4	3.2
UUI0410	*Pardosa palustris*	Female	Russia	Magadan	11.5	9	11.5					2.5	3.2
UUI0411	*Pardosa palustris*	Female	Russia	Magadan	11.1	8.5	11.7					2.3	3
UUI0412	*Pardosa palustris*	Female	Russia	Magadan	11.7	9	12					2.4	2.8
UUI0413	*Pardosa palustris*	Female	Russia	Chukotka	12	8.3	11.2					2.3	2.7
UUI0414	*Pardosa palustris*	Female	Russia	Chukotka	11.8	8.8	11					2.3	3
UUI0415	*Pardosa palustris*	Female	Russia	Chukotka	10.5	8.5	11					2.2	3
UUI0416	*Pardosa palustris*	Female	Russia	Chukotka	11.4	8.5	11					2.2	3
UUI0417	*Pardosa palustris*	Female	Russia	Chukotka	11.5	8.9	11.5					2.4	2.9
UUI0418	*Pardosa palustris*	Female	Russia	Magadan	11.5	9	10.9					2.4	3.2

UUI0419	*Pardosa palustris*	Female	Russia	Magadan	11	9.1	11.5					2.3	3
UUI0420	*Pardosa palustris*	Female	Russia	Magadan	11	8.8	11.5					2.2	3.1
UUI0421	*Pardosa palustris*	Female	Russia	Chukotka	10.9	8.5	11.5					2.3	2.8
UUI0422	*Pardosa palustris*	Female	Russia	Chukotka	11.2	8.9	11.5					2.3	3
UUI0423	*Pardosa palustris*	Female	Russia	Chukotka	11	9.2	12					2.2	2.8
UUI0424	*Pardosa palustris*	Female	Russia	Chukotka	12	9.5	12					2.4	3
UUI0425	*Pardosa palustris*	Female	Russia	Chukotka	11.8	9.2	11.6					2.1	3
UUI0426	*Pardosa palustris*	Female	Russia	Chukotka	11	8.5	11					2.5	2.9
MAR_12	*Pardosa palustris*	Female	Russia	Magadan	11.2	9.1	11.5					2.3	2.8
MAR_14	*Pardosa palustris*	Female	Russia	Magadan	12.5	10	12.2					2.4	3.1
UUI0427	*Pardosa palustris*	Male	Russia	Magadan	10.5	7.8	11	4.9	1.3	3.4	2.9		
UUI0428	*Pardosa palustris*	Male	Russia	Magadan	10.5	7.5	11.2	4.9	1.4	2.8	2.9		
UUI0429	*Pardosa palustris*	Male	Russia	Magadan	10.2	9	11.5	5.3	1.4	3.5	3		
UUI0430	*Pardosa palustris*	Male	Russia	Magadan	11	9.3	12.5	5.4	1.5	3.4	3.1		
UUI0431	*Pardosa palustris*	Male	Russia	Magadan	10.9	9	11.2	5.3	1.1	3.4	3		
UUI0432	*Pardosa palustris*	Male	Russia	Magadan	10.5	8	10	5	1	3.4	3		
UUI0433	*Pardosa palustris*	Male	Russia	Magadan	13	10	13.1	5.7	1.5	3.7	3.1		
UUI0434	*Pardosa palustris*	Male	Russia	Magadan	11	8	10.8	5	1.1	3.2	2.9		
UUI0435	*Pardosa palustris*	Male	Russia	Magadan	10.5	7.8	11	4.8	1.1	3	3		
UUI0436	*Pardosa palustris*	Male	Russia	Magadan	11	8.6	12.5	5.4	1.3	3.5	3.1		
UUI0437	*Pardosa palustris*	Male	Russia	Chukotka	11.5	9	12	5	1.3	3.1	2.7		
UUI0438	*Pardosa palustris*	Male	Russia	Chukotka	12	8.9	12	5.5	1.4	3.2	3		
UUI0439	*Pardosa palustris*	Male	Russia	Chukotka	11.9	9.1	12	5.1	1.2	3	2.9		
UUI0440	*Pardosa palustris*	Male	Russia	Chukotka	11.5	8.5	12.5	5	1.3	3.3	2.7		
UUI0441	*Pardosa palustris*	Male	Russia	Chukotka	12	7.8	11.5	5.2	1.3	3.4	2.9		
UUI0442	*Pardosa palustris*	Male	Russia	Magadan	11	9.2	12	5	1.4	3.1	3		
UUI0443	*Pardosa palustris*	Male	Russia	Magadan	11.5	9	12.2	5.3	1.5	3.5	3		
UUI0444	*Pardosa palustris*	Male	Russia	Magadan	11.8	9.6	12.9	5.2	1.3	3.2	3.1		
UUI0445	*Pardosa palustris*	Male	Russia	Magadan	11.5	9	11.9	5.5	1.4	3.5	3.1		
UUI0446	*Pardosa palustris*	Male	Russia	Magadan	10.8	8	11.2	5	1.2	3.1	2.9		
MAR_11	*Pardosa palustris*	Male	Russia	Magadan	11.5	8.6	12	5.2	1.4	3	2.8		

MAR_13	*Pardosa palustris*	Male	Russia	Magadan	12	9.2	12.5	5.4	1.4	3	2.9		
JPF_11	*Pardosa palustris*	Male	Faroeae Islands		11.8	8.8	11	5.2	1.4	3	2.9		
JPF_12	*Pardosa palustris*	Female	Faroeae Islands		11.7	9	10.9					2.4	2.9
JPF_10	*Pardosa palustris*	Female	Faroeae Islands		10.8	8.5	10.8					2.4	3.3
JPF_13	*Pardosa palustris*	Male	Great Britain	Essex	11.2	8.2	11	4.8	1.2	3.2	2.9		
JPF_14	*Pardosa palustris*	Male	France		11	9	11.1	5.3	1.2	3.2	2.8		

Table S6. Summary of ANCOVA and t-test for morphological measurements.

Characters	Finland		Russia		Bartlett test	ANCOVA		t-test
	mean	sd	mean	sd	p	F	p	p
P. riparia males								
CarLen	16.7	0.931	17.1	0.968	0.8891	Covariate		0.189
CarWid	11.8	0.529	12.5	0.855	0.09113	5.133	**0.032**	**0.012**
PatTib	15	0.579	17.3	1.38	**0.003426**	28.824	**1.27E-05**	**1.24E-05**
CymbLen	6.75	0.267	7.19	0.428	0.09654	7.346	**0.012**	**0.00384**
CymbTip	2.29	0.229	2.34	0.34	0.1635	0.012	0.912	0.571
BulbLen	3.99	0.263	4.31	0.246	0.8162	8.151	**0.008**	**0.0047**
BulbWid	3.37	0.247	3.65	0.207	0.5208	9.06	**0.006**	**0.00219**
ApophLen	4.157	0.293	4.167	0.32	0.7492	0.002	0.963	0.934
P. riparia females								
CarLen	16.9	0.866	18.3	0.99	0.6294	Covariate		**3.91E-04**
CarWid	12.7	0.16	14.7	0.167	0.8555	58.03	**4.37E-08**	**1E-10**
PatTib	15.5	0.208	18.3	0.217	0.9364	69.926	**7.63E-09**	**5.57E-12**
SeptLen	3.13	0.105	3.47	0.109	0.9791	3.97	0.057	**0.00392**
SeptWid	2.1	0.082	1.93	0.0856	0.2856	1.809	0.19	0.175
P. palustris males								
CarLen	11.2	0.576	11.3	0.68	0.435	Covariate		0.506
CarWid	8.45	0.433	8.68	0.669	**0.04**	1.902	0.175	0.173
PatTib	11.3	0.369	11.8	0.756	**0.0008719**	12.509	**9.52E-04**	**0.00577**
CymbLen	4.98	0.177	5.19	0.236	0.1785	14.728	**3.85E-04**	**0.00146**
CymbTip	1.12	0.103	1.32	0.141	0.1386	27.816	**3.67E-06**	**8.94E-06**
BulbLen	3.08	0.163	3.26	0.228	0.1134	9.719	**0.003**	**0.00341**
BulbWid	2.97	0.129	2.95	0.118	0.6898	0.374	0.544	0.683
P. palustris females								
CarLen	10.8	0.538	11.4	0.458	0.4693	Covariate		3.15E-04
CarWid	8.77	0.42	8.82	0.42	0.997	0.149	0.701	**0.00699**
PatTib	10.6	0.55	11.4	0.39	0.1259	28.711	**3.76E-06**	**9.1E-09**
SeptLen	2.35	0.08	2.32	0.11	0.1904	1.013	0.32	0.799
SeptWid	3.09	0.16	2.95	0.14	0.5563	6.446	**0.015**	0.109

Table S7. GMYC analysis summary

GMYC analysis	Riparia group	Pullata only	Palustris only	Monticola group	Pullata AND Monticola groups
likelihood of null model	190.6797	1483.335	435.7763	890.5629	2521.164
maximum likelihood of GMYC model	192.027	1484.82	436.1033	893.5164	2523.41
likelihood ratio	2.6947	2.9694	0.654	5.9069	4.4913
result of LR test	0.2599 n.s.	0.2266 n.s.	0.7211 n.s.	0.0522 n.s.	0.1059 n.s.
number of ML clusters	4	17	15	21	6
confidence interval	1-7	1-38	1-16	3-28	3-61
number of ML entities	6	19	23	32	6
confidence interval	1-21	1-62	1-44	3-49	3-106
threshold time	-0.0003	-0.0011	-0.0001	-0.0006	-0.0126
P. palustris taxonomy, number of species	NA	NA	23	11	1
P. palustris lumped with other species	NA	NA	NA	NO	NO
P. riparia taxonomy, number of species	6	3	NA	NA	1
P. riparia lumped with other species	NA	NO	NA	NA	YES

Table S8. bPTP analysis summary. NA - not available

PTP analysis	Riparia group	Pullata only	Palustris only	Monticola group	Pullata AND Monticola groups
Accptance rate	0.78297	0.916	0.8332	0.88044	0.90793
Merge	49558	49859	49966	49862	49969
Split	49937	50141	50034	50138	50031
Estimated number of species	1-22	56-121	5-43	28-85	95-178
Mean number of species	12.55	87.74	25.59	55.86	136.73
P. palustris taxonomy Bayesian. number of species	NA	NA	41	41	30
P. palustris lumped with other species Bayesian	NA	NA	NA	NO	NO
P. palustris taxonomy ML. number of species	NA	NA	5	1	1
P. palustris lumped with other species. ML	NA	NA	NA	NO	NO
P. riparia taxonomy Bayesian. number of species	20	20	NA	NA	16
P. riparia lumped with other species Bayesian	NA	NO	NA	NA	NO
P. riparia taxonomy ML. number of species	2	2	NA	NA	2
P. riparia lumped with other species ML	NA	YES	NA	NA	NO

BFD* runs

Specimens included in the runs are in Table S1. We followed Rough Guide to SNAPP (https://github.com/BEAST2-Dev/SNAPP/releases/download/v1.2.0/SNAPP.pdf) and BFD tutorial (http://evomicsorg.wpengine.netdna-cdn.com/wp-content/uploads/2018/01/BFD-tutorial-1.pdf) to set up the parameters of the runs. Model parameters: mutation rates u and v were set to 1 and not sampled, coalescent rate was set to 10 and sampled, non-polymorphic sites were not included, log likelihood correction was used. Priors: alpha = 1, beta = 125, Kappa = 1, Lambda = 10, all not sampled. MCMC chain length = 2 000 000, number of steps = 48. We used values mentioned in tutorial as we do not have good estimates of these parameters for the target species.

Marginal likelihoods for *P. ripara*: 1 species: -6573.768635, 2 species: -6383.252103; *P. palustris*: 1 species: -5562,85564, 2 species: -5415,244414.
