# Supplementary figures and images for: Relevance of ddRADseq method for species and population delimitation of closely related and widely distributed wolf spiders (Araneae, Lycosidae)
Source: Sci Rep. 2021 Jan 26;11:2177. doi: 10.1038/s41598-021-81788-2 (PMC7838170; doi:10.1038/s41598-021-81788-2)

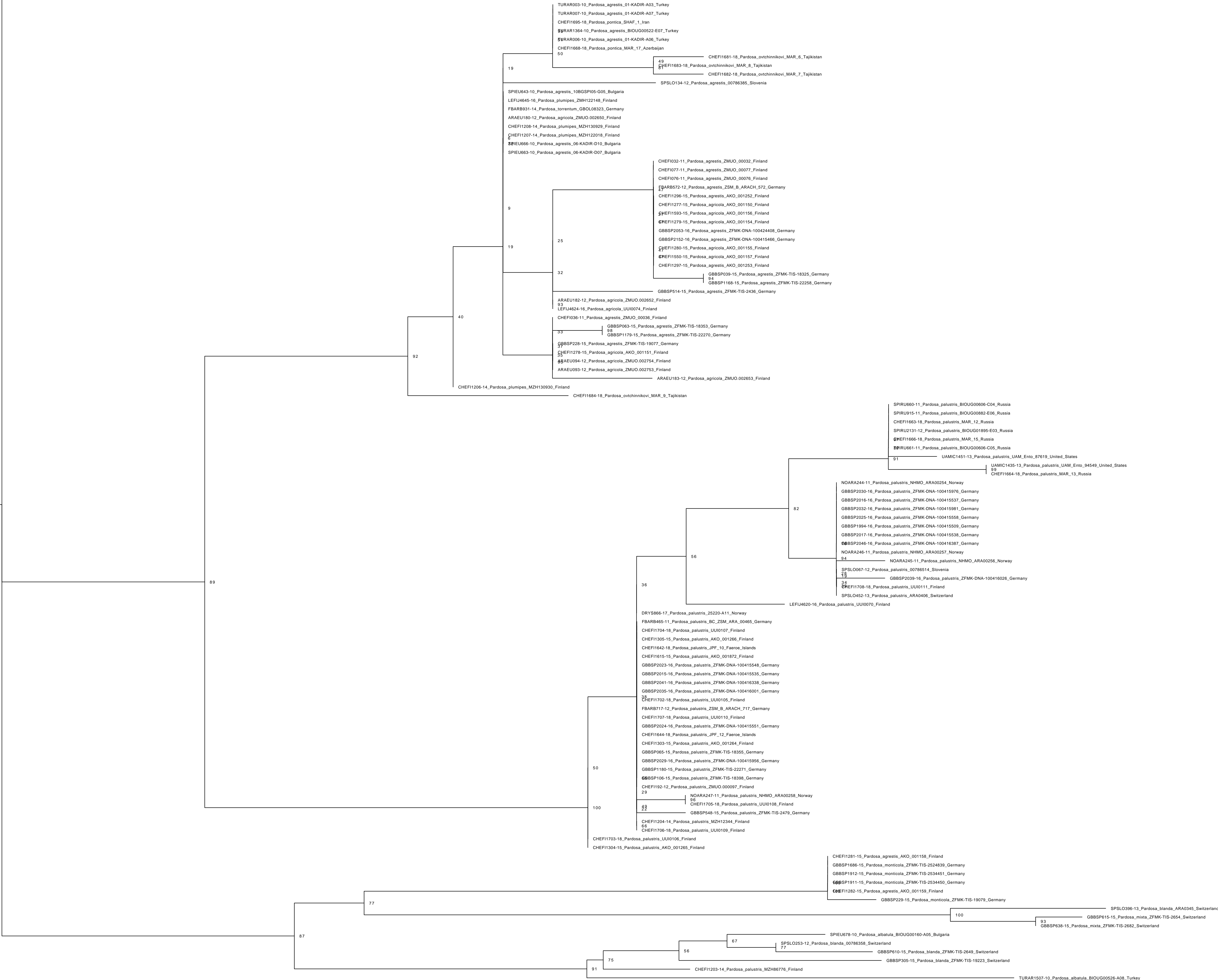

Supplement: Supplementary file 3 — Supplementary Information 3. [file 41598_2021_81788_MOESM3_ESM.zip › Supplementary_material_3_COI_ML_trees/P.monticola_group_COI_ML_tree.pdf]

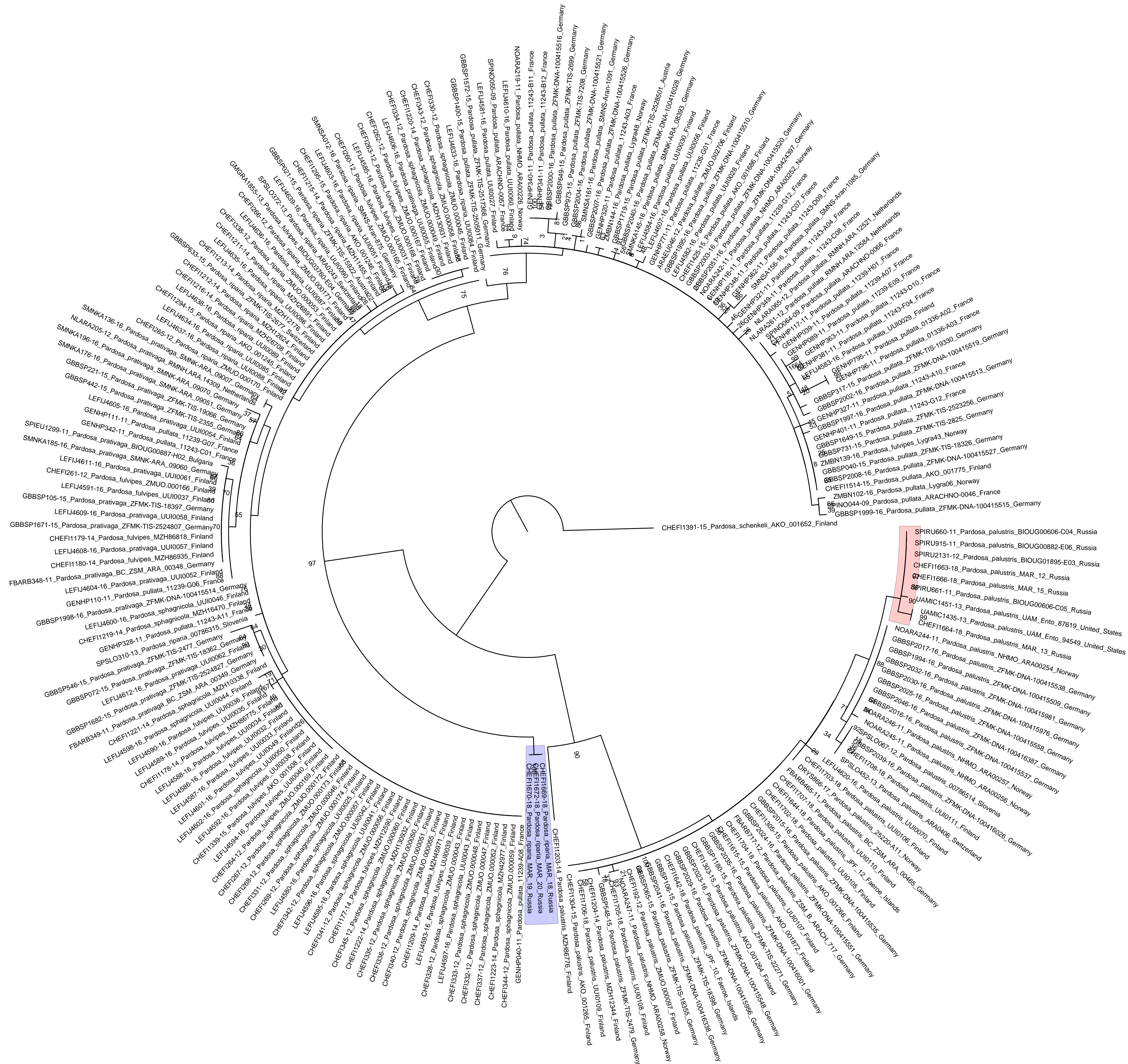

Supplement: Supplementary file 3 — Supplementary Information 3. [file 41598_2021_81788_MOESM3_ESM.zip › Supplementary_material_3_COI_ML_trees/P.riparia_and_P.palustris_COI_ML_tree.pdf]
