# Supplementary material for: Relevance of ddRADseq method for species and population delimitation of closely related and widely distributed wolf spiders (Araneae, Lycosidae)
Source: Sci Rep. 2021 Jan 26;11:2177. doi: 10.1038/s41598-021-81788-2 (PMC7838170; doi:10.1038/s41598-021-81788-2)
Supplement: Supplementary file 4 — Supplementary Information 4. [file 41598_2021_81788_MOESM4_ESM.zip › Supplementary_material_4_BOLD_ID_Trees/Palustris_BOLD_ID_Tree.pdf]

# BOLD TaxonID Tree

Title : Tree Result - Search: Sample IDs; Include public records (95 records returned) (95 records selected)
Date : 15-Oct-2020
Data Type : Nucleotide
Distance Model : Kimura 2 Parameter
Marker : COI-5P
Colourization : [blue]=Stop Codons [red]=Contamination or misidentification

Label : Process ID
Label : Taxon
Label : Country
Label : Barcode Cluster (BIN)

Sequence Count : 95
Species count : 10
Genus count : 1
Family count : 1
Unidentified : 0

BIN Count : 5

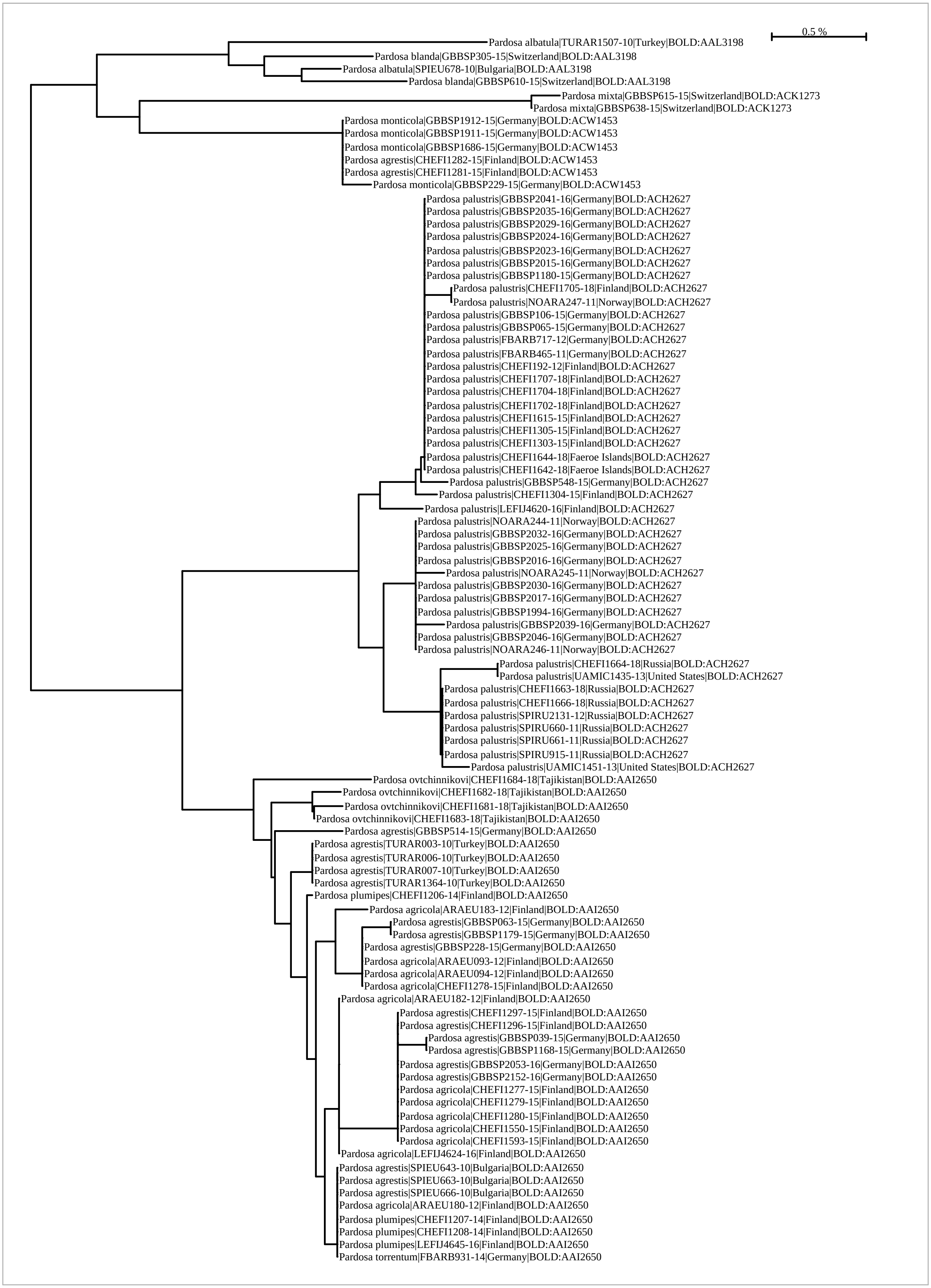
0.5 %
Pardosa albatula|TURAR1507-10|Turkey|BOLD:AAL3198
Pardosa blanda|GBBSP305-15|Switzerland|BOLD:AAL3198
Pardosa albatula|SPIEU678-10|Bulgaria|BOLD:AAL3198
Pardosa blanda|GBBSP610-15|Switzerland|BOLD:AAL3198
Pardosa mixta|GBBSP615-15|Switzerland|BOLD:ACK1273
Pardosa mixta|GBBSP638-15|Switzerland|BOLD:ACK1273
Pardosa monticola|GBBSP1912-15|Germany|BOLD:ACW1453
Pardosa monticola|GBBSP1911-15|Germany|BOLD:ACW1453
Pardosa monticola|GBBSP1686-15|Germany|BOLD:ACW1453
Pardosa agrestis|CHEFI1282-15|Finland|BOLD:ACW1453
Pardosa agrestis|CHEFI1281-15|Finland|BOLD:ACW1453
Pardosa monticola|GBBSP229-15|Germany|BOLD:ACW1453
Pardosa palustris|GBBSP2041-16|Germany|BOLD:ACH2627
Pardosa palustris|GBBSP2035-16|Germany|BOLD:ACH2627
Pardosa palustris|GBBSP2029-16|Germany|BOLD:ACH2627
Pardosa palustris|GBBSP2024-16|Germany|BOLD:ACH2627
Pardosa palustris|GBBSP2023-16|Germany|BOLD:ACH2627
Pardosa palustris|GBBSP2015-16|Germany|BOLD:ACH2627
Pardosa palustris|GBBSP1180-15|Germany|BOLD:ACH2627
Pardosa palustris|CHEFI1705-18|Finland|BOLD:ACH2627
Pardosa palustris|NOARA247-11|Norway|BOLD:ACH2627
Pardosa palustris|GBBSP106-15|Germany|BOLD:ACH2627
Pardosa palustris|GBBSP065-15|Germany|BOLD:ACH2627
Pardosa palustris|FBARB717-12|Germany|BOLD:ACH2627
Pardosa palustris|FBARB465-11|Germany|BOLD:ACH2627
Pardosa palustris|CHEFI192-12|Finland|BOLD:ACH2627
Pardosa palustris|CHEFI1707-18|Finland|BOLD:ACH2627
Pardosa palustris|CHEFI1704-18|Finland|BOLD:ACH2627
Pardosa palustris|CHEFI1702-18|Finland|BOLD:ACH2627
Pardosa palustris|CHEFI1615-15|Finland|BOLD:ACH2627
Pardosa palustris|CHEFI1305-15|Finland|BOLD:ACH2627
Pardosa palustris|CHEFI1303-15|Finland|BOLD:ACH2627
Pardosa palustris|CHEFI1644-18|Faeroe Islands|BOLD:ACH2627
Pardosa palustris|CHEFI1642-18|Faeroe Islands|BOLD:ACH2627
Pardosa palustris|GBBSP548-15|Germany|BOLD:ACH2627
Pardosa palustris|CHEFI1304-15|Finland|BOLD:ACH2627
Pardosa palustris|LEFIJ4620-16|Finland|BOLD:ACH2627
Pardosa palustris|NOARA244-11|Norway|BOLD:ACH2627
Pardosa palustris|GBBSP2032-16|Germany|BOLD:ACH2627
Pardosa palustris|GBBSP2025-16|Germany|BOLD:ACH2627
Pardosa palustris|GBBSP2016-16|Germany|BOLD:ACH2627
Pardosa palustris|NOARA245-11|Norway|BOLD:ACH2627
Pardosa palustris|GBBSP2030-16|Germany|BOLD:ACH2627
Pardosa palustris|GBBSP2017-16|Germany|BOLD:ACH2627
Pardosa palustris|GBBSP1994-16|Germany|BOLD:ACH2627
Pardosa palustris|GBBSP2039-16|Germany|BOLD:ACH2627
Pardosa palustris|GBBSP2046-16|Germany|BOLD:ACH2627
Pardosa palustris|NOARA246-11|Norway|BOLD:ACH2627
Pardosa palustris|CHEFI1664-18|Russia|BOLD:ACH2627
Pardosa palustris|UAMIC1435-13|United States|BOLD:ACH2627
Pardosa palustris|CHEFI1663-18|Russia|BOLD:ACH2627
Pardosa palustris|CHEFI1666-18|Russia|BOLD:ACH2627
Pardosa palustris|SPIRU2131-12|Russia|BOLD:ACH2627
Pardosa palustris|SPIRU660-11|Russia|BOLD:ACH2627
Pardosa palustris|SPIRU661-11|Russia|BOLD:ACH2627
Pardosa palustris|SPIRU915-11|Russia|BOLD:ACH2627
Pardosa palustris|UAMIC1451-13|United States|BOLD:ACH2627
Pardosa ovtchinnikovi|CHEFI1684-18|Tajikistan|BOLD:AAI2650
Pardosa ovtchinnikovi|CHEFI1682-18|Tajikistan|BOLD:AAI2650
Pardosa ovtchinnikovi|CHEFI1681-18|Tajikistan|BOLD:AAI2650
Pardosa ovtchinnikovi|CHEFI1683-18|Tajikistan|BOLD:AAI2650
Pardosa agrestis|GBBSP514-15|Germany|BOLD:AAI2650
Pardosa agrestis|TURAR003-10|Turkey|BOLD:AAI2650
Pardosa agrestis|TURAR006-10|Turkey|BOLD:AAI2650
Pardosa agrestis|TURAR007-10|Turkey|BOLD:AAI2650
Pardosa agrestis|TURAR1364-10|Turkey|BOLD:AAI2650
Pardosa plumipes|CHEFI1206-14|Finland|BOLD:AAI2650
Pardosa agricola|ARAEU183-12|Finland|BOLD:AAI2650
Pardosa agrestis|GBBSP063-15|Germany|BOLD:AAI2650
Pardosa agrestis|GBBSP1179-15|Germany|BOLD:AAI2650
Pardosa agrestis|GBBSP228-15|Germany|BOLD:AAI2650
Pardosa agricola|ARAEU093-12|Finland|BOLD:AAI2650
Pardosa agricola|ARAEU094-12|Finland|BOLD:AAI2650
Pardosa agricola|CHEFI1278-15|Finland|BOLD:AAI2650
Pardosa agricola|ARAEU182-12|Finland|BOLD:AAI2650
Pardosa agrestis|CHEFI1297-15|Finland|BOLD:AAI2650
Pardosa agrestis|CHEFI1296-15|Finland|BOLD:AAI2650
Pardosa agrestis|GBBSP039-15|Germany|BOLD:AAI2650
Pardosa agrestis|GBBSP1168-15|Germany|BOLD:AAI2650
Pardosa agrestis|GBBSP2053-16|Germany|BOLD:AAI2650
Pardosa agrestis|GBBSP2152-16|Germany|BOLD:AAI2650
Pardosa agricola|CHEFI1277-15|Finland|BOLD:AAI2650
Pardosa agricola|CHEFI1279-15|Finland|BOLD:AAI2650
Pardosa agricola|CHEFI1280-15|Finland|BOLD:AAI2650
Pardosa agricola|CHEFI1550-15|Finland|BOLD:AAI2650
Pardosa agricola|CHEFI1593-15|Finland|BOLD:AAI2650
Pardosa agricola|LEFIJ4624-16|Finland|BOLD:AAI2650
Pardosa agrestis|SPIEU643-10|Bulgaria|BOLD:AAI2650
Pardosa agrestis|SPIEU663-10|Bulgaria|BOLD:AAI2650
Pardosa agrestis|SPIEU666-10|Bulgaria|BOLD:AAI2650
Pardosa agricola|ARAEU180-12|Finland|BOLD:AAI2650
Pardosa plumipes|CHEFI1207-14|Finland|BOLD:AAI2650
Pardosa plumipes|CHEFI1208-14|Finland|BOLD:AAI2650
Pardosa plumipes|LEFIJ4645-16|Finland|BOLD:AAI2650
Pardosa torrentum|FBARB931-14|Germany|BOLD:AAI2650
