# Supplementary material for: Relevance of ddRADseq method for species and population delimitation of closely related and widely distributed wolf spiders (Araneae, Lycosidae)
Source: Sci Rep. 2021 Jan 26;11:2177. doi: 10.1038/s41598-021-81788-2 (PMC7838170; doi:10.1038/s41598-021-81788-2)
Supplement: Supplementary file 4 — Supplementary Information 4. [file 41598_2021_81788_MOESM4_ESM.zip › Supplementary_material_4_BOLD_ID_Trees/Riparia_BOLD_ID_Tree.pdf]

BOLD TaxonID Tree

Title	:	Tree for Barcode Index Number - BOLD:AAF7515
Date	:	3-December-2018
Data Type	:	Nucleotide
Distance Model	:	Kimura 2 Parameter
Codon Positions	:	1st, 2nd, 3rd
Labels	:	Country & Province, ProcessID, BIN uri

Sequence Count	:	226
Species count	:	6
Genus count	:	1
Family count	:	1
Unidentified	:	9

BIN Count	:	2

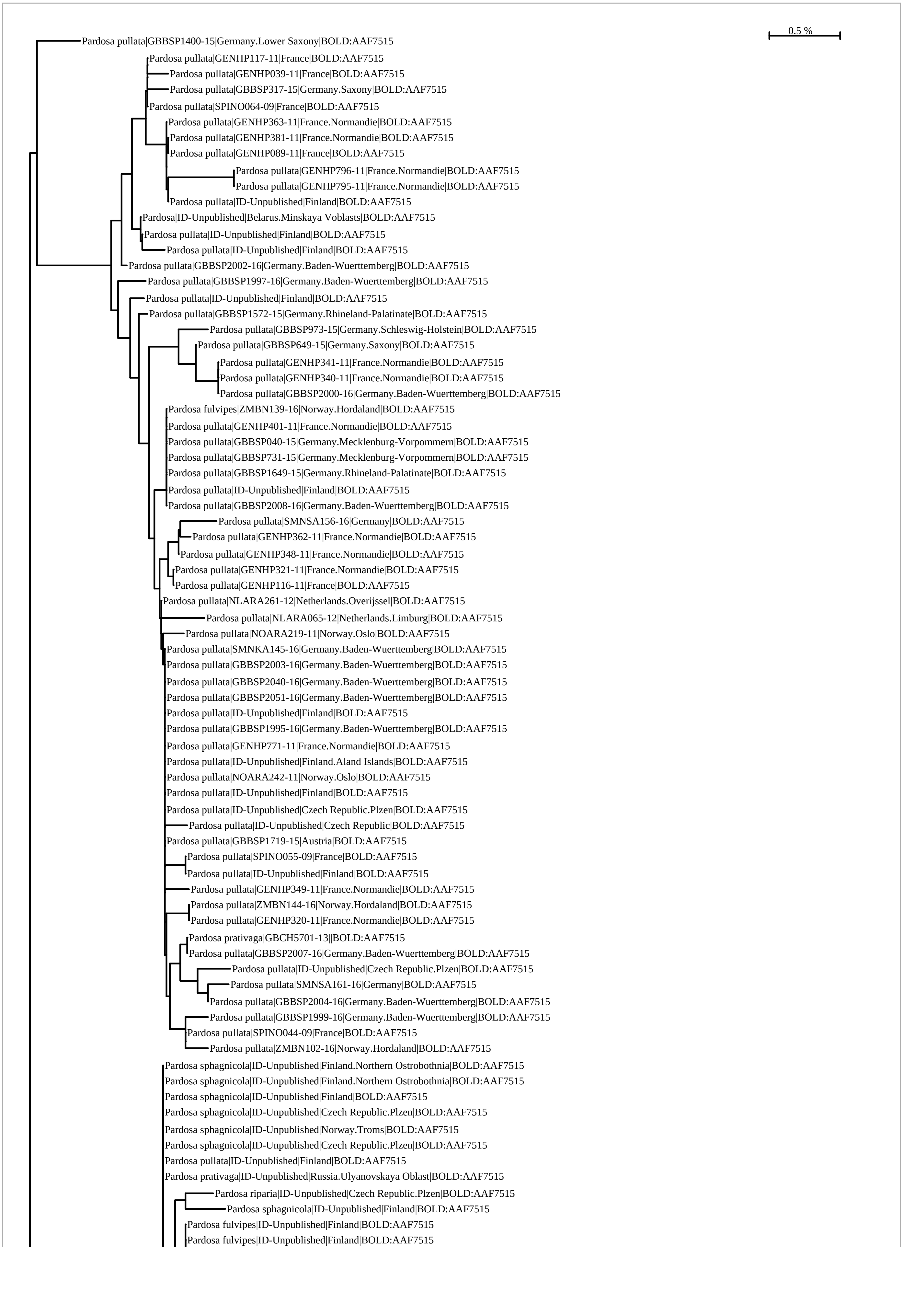
0.5 %
Pardosa pullata|GBBSP1400-15|Germany.Lower Saxony|BOLD:AAF7515
Pardosa pullata|GENHP117-11|France|BOLD:AAF7515
Pardosa pullata|GENHP039-11|France|BOLD:AAF7515
Pardosa pullata|GBBSP317-15|Germany.Saxony|BOLD:AAF7515
Pardosa pullata|SPINO064-09|France|BOLD:AAF7515
Pardosa pullata|GENHP363-11|France.Normandie|BOLD:AAF7515
Pardosa pullata|GENHP381-11|France.Normandie|BOLD:AAF7515
Pardosa pullata|GENHP089-11|France|BOLD:AAF7515
Pardosa pullata|GENHP796-11|France.Normandie|BOLD:AAF7515
Pardosa pullata|GENHP795-11|France.Normandie|BOLD:AAF7515
Pardosa pullata|ID-Unpublished|Finland|BOLD:AAF7515
Pardosa|ID-Unpublished|Belarus.Minskaya Voblasts|BOLD:AAF7515
Pardosa pullata|ID-Unpublished|Finland|BOLD:AAF7515
Pardosa pullata|ID-Unpublished|Finland|BOLD:AAF7515
Pardosa pullata|GBBSP2002-16|Germany.Baden-Wuerttemberg|BOLD:AAF7515
Pardosa pullata|GBBSP1997-16|Germany.Baden-Wuerttemberg|BOLD:AAF7515
Pardosa pullata|ID-Unpublished|Finland|BOLD:AAF7515
Pardosa pullata|GBBSP1572-15|Germany.Rhineland-Palatinate|BOLD:AAF7515
Pardosa pullata|GBBSP973-15|Germany.Schleswig-Holstein|BOLD:AAF7515
Pardosa pullata|GBBSP649-15|Germany.Saxony|BOLD:AAF7515
Pardosa pullata|GENHP341-11|France.Normandie|BOLD:AAF7515
Pardosa pullata|GENHP340-11|France.Normandie|BOLD:AAF7515
Pardosa pullata|GBBSP2000-16|Germany.Baden-Wuerttemberg|BOLD:AAF7515
Pardosa fulvipes|ZMBN139-16|Norway.Hordaland|BOLD:AAF7515
Pardosa pullata|GENHP401-11|France.Normandie|BOLD:AAF7515
Pardosa pullata|GBBSP040-15|Germany.Mecklenburg-Vorpommern|BOLD:AAF7515
Pardosa pullata|GBBSP731-15|Germany.Mecklenburg-Vorpommern|BOLD:AAF7515
Pardosa pullata|GBBSP1649-15|Germany.Rhineland-Palatinate|BOLD:AAF7515
Pardosa pullata|ID-Unpublished|Finland|BOLD:AAF7515
Pardosa pullata|GBBSP2008-16|Germany.Baden-Wuerttemberg|BOLD:AAF7515
Pardosa pullata|SMNSA156-16|Germany|BOLD:AAF7515
Pardosa pullata|GENHP362-11|France.Normandie|BOLD:AAF7515
Pardosa pullata|GENHP348-11|France.Normandie|BOLD:AAF7515
Pardosa pullata|GENHP321-11|France.Normandie|BOLD:AAF7515
Pardosa pullata|GENHP116-11|France|BOLD:AAF7515
Pardosa pullata|NLARA261-12|Netherlands.Overijssel|BOLD:AAF7515
Pardosa pullata|NLARA065-12|Netherlands.Limburg|BOLD:AAF7515
Pardosa pullata|NOARA219-11|Norway.Oslo|BOLD:AAF7515
Pardosa pullata|SMNKA145-16|Germany.Baden-Wuerttemberg|BOLD:AAF7515
Pardosa pullata|GBBSP2003-16|Germany.Baden-Wuerttemberg|BOLD:AAF7515
Pardosa pullata|GBBSP2040-16|Germany.Baden-Wuerttemberg|BOLD:AAF7515
Pardosa pullata|GBBSP2051-16|Germany.Baden-Wuerttemberg|BOLD:AAF7515
Pardosa pullata|ID-Unpublished|Finland|BOLD:AAF7515
Pardosa pullata|GBBSP1995-16|Germany.Baden-Wuerttemberg|BOLD:AAF7515
Pardosa pullata|GENHP771-11|France.Normandie|BOLD:AAF7515
Pardosa pullata|ID-Unpublished|Finland.Aland Islands|BOLD:AAF7515
Pardosa pullata|NOARA242-11|Norway.Oslo|BOLD:AAF7515
Pardosa pullata|ID-Unpublished|Finland|BOLD:AAF7515
Pardosa pullata|ID-Unpublished|Czech Republic.Plzen|BOLD:AAF7515
Pardosa pullata|ID-Unpublished|Czech Republic|BOLD:AAF7515
Pardosa pullata|GBBSP1719-15|Austria|BOLD:AAF7515
Pardosa pullata|SPINO055-09|France|BOLD:AAF7515
Pardosa pullata|ID-Unpublished|Finland|BOLD:AAF7515
Pardosa pullata|GENHP349-11|France.Normandie|BOLD:AAF7515
Pardosa pullata|ZMBN144-16|Norway.Hordaland|BOLD:AAF7515
Pardosa pullata|GENHP320-11|France.Normandie|BOLD:AAF7515
Pardosa prativaga|GBCH5701-13||BOLD:AAF7515
Pardosa pullata|GBBSP2007-16|Germany.Baden-Wuerttemberg|BOLD:AAF7515
Pardosa pullata|ID-Unpublished|Czech Republic.Plzen|BOLD:AAF7515
Pardosa pullata|SMNSA161-16|Germany|BOLD:AAF7515
Pardosa pullata|GBBSP2004-16|Germany.Baden-Wuerttemberg|BOLD:AAF7515
Pardosa pullata|GBBSP1999-16|Germany.Baden-Wuerttemberg|BOLD:AAF7515
Pardosa pullata|SPINO044-09|France|BOLD:AAF7515
Pardosa pullata|ZMBN102-16|Norway.Hordaland|BOLD:AAF7515
Pardosa sphagnicola|ID-Unpublished|Finland.Northern Ostrobothnia|BOLD:AAF7515
Pardosa sphagnicola|ID-Unpublished|Finland.Northern Ostrobothnia|BOLD:AAF7515
Pardosa sphagnicola|ID-Unpublished|Finland|BOLD:AAF7515
Pardosa sphagnicola|ID-Unpublished|Czech Republic.Plzen|BOLD:AAF7515
Pardosa sphagnicola|ID-Unpublished|Norway.Troms|BOLD:AAF7515
Pardosa sphagnicola|ID-Unpublished|Czech Republic.Plzen|BOLD:AAF7515
Pardosa pullata|ID-Unpublished|Finland|BOLD:AAF7515
Pardosa prativaga|ID-Unpublished|Russia.Ulyanovskaya Oblast|BOLD:AAF7515
Pardosa riparia|ID-Unpublished|Czech Republic.Plzen|BOLD:AAF7515
Pardosa sphagnicola|ID-Unpublished|Finland|BOLD:AAF7515
Pardosa fulvipes|ID-Unpublished|Finland|BOLD:AAF7515
Pardosa fulvipes|ID-Unpublished|Finland|BOLD:AAF7515

[illegible]
Pardosa fulvipes|ID-Unpublished|Finland|BOLD:AAF7515
Pardosa fulvipes|ID-Unpublished|Finland|BOLD:AAF7515
Pardosa fulvipes|ID-Unpublished|Finland|BOLD:AAF7515
Pardosa fulvipes|ID-Unpublished|Finland|BOLD:AAF7515
Pardosa fulvipes|ID-Unpublished|Norway.Vestfold|BOLD:AAF7515
Pardosa fulvipes|ID-Unpublished|Finland.Northern Ostrobothnia|BOLD:AAF7515
Pardosa fulvipes|ID-Unpublished|Finland|BOLD:AAF7515
Pardosa fulvipes|ID-Unpublished|Finland|BOLD:AAF7515
Pardosa fulvipes|ID-Unpublished|Finland|BOLD:AAF7515
Pardosa fulvipes|ID-Unpublished|Finland|BOLD:AAF7515
Pardosa fulvipes|ID-Unpublished|Finland|BOLD:AAF7515
Pardosa fulvipes|ID-Unpublished|Finland|BOLD:AAF7515
Pardosa sphagnicola|ID-Unpublished|Finland|BOLD:AAF7515
Pardosa prativaga|ID-Unpublished|Italy|BOLD:AAF7515
Pardosa prativaga|GBBSP546-15|Germany.Saxony|BOLD:AAF7515
Pardosa|ID-Unpublished|Belarus.Minskaya Voblasts|BOLD:AAF7515
Pardosa|ID-Unpublished|Belarus.Minskaya Voblasts|BOLD:AAF7515
Pardosa prativaga|ID-Unpublished|Finland|BOLD:AAF7515
Pardosa prativaga|FBARB349-11|Germany.Saxony-Anhalt|BOLD:AAF7515
Pardosa prativaga|GBBSP072-15|Germany.Mecklenburg-Vorpommern|BOLD:AAF7515
Pardosa prativaga|GBBSP1682-15|Germany.Schleswig-Holstein|BOLD:AAF7515
Pardosa sphagnicola|ID-Unpublished|Finland|BOLD:AAF7515
Pardosa|ID-Unpublished|Belarus.Minskaya Voblasts|BOLD:AAF7515
Pardosa sphagnicola|ID-Unpublished|Norway.Hedmark|BOLD:AAF7515
Pardosa sphagnicola|ID-Unpublished|Germany.Saxony-Anhalt|BOLD:AAF7515
Pardosa sphagnicola|ID-Unpublished|Finland.North Karelia|BOLD:AAF7515
Pardosa sphagnicola|ID-Unpublished|Finland.North Karelia|BOLD:AAF7515
Pardosa sphagnicola|ID-Unpublished|Finland.Northern Ostrobothnia|BOLD:AAF7515
Pardosa sphagnicola|ID-Unpublished|Finland.Northern Ostrobothnia|BOLD:AAF7515
Pardosa prativaga|ID-Unpublished|Russia.Ulyanovskaya Oblast|BOLD:AAF7515
Pardosa fulvipes|ID-Unpublished|Finland|BOLD:AAF7515
Pardosa fulvipes|ID-Unpublished|Finland|BOLD:AAF7515
Pardosa prativaga|ID-Unpublished|Russia.Ulyanovskaya Oblast|BOLD:AAF7515
Pardosa pullata|GENHP040-11|France|BOLD:AAF7515
Pardosa riparia|ID-Unpublished|Norway.Oppland|BOLD:AAF7515
Pardosa sphagnicola|ID-Unpublished|Finland.Northern Ostrobothnia|BOLD:AAF7515
Pardosa sphagnicola|ID-Unpublished|Finland|BOLD:AAF7515
Pardosa sphagnicola|ID-Unpublished|Czech Republic.Plzen|BOLD:AAF7515
Pardosa sphagnicola|ID-Unpublished|Finland.North Karelia|BOLD:AAF7515
Pardosa sphagnicola|ID-Unpublished|Czech Republic.Plzen|BOLD:AAF7515
Pardosa sphagnicola|ID-Unpublished|Finland|BOLD:AAF7515
Pardosa sphagnicola|ID-Unpublished|Finland|BOLD:AAF7515
Pardosa sphagnicola|ID-Unpublished|Finland.Northern Ostrobothnia|BOLD:AAF7515
Pardosa sphagnicola|ID-Unpublished|Finland.North Karelia|BOLD:AAF7515
Pardosa sphagnicola|ID-Unpublished|Finland.Northern Ostrobothnia|BOLD:AAF7515
Pardosa sphagnicola|ID-Unpublished|Finland.Northern Ostrobothnia|BOLD:AAF7515
Pardosa sphagnicola|ID-Unpublished|Finland.North Karelia|BOLD:AAF7515
Pardosa sphagnicola|ID-Unpublished|Finland.Northern Ostrobothnia|BOLD:AAF7515
Pardosa sphagnicola|ID-Unpublished|Finland|BOLD:AAF7515
Pardosa sphagnicola|ID-Unpublished|Finland.Northern Ostrobothnia|BOLD:AAF7515
Pardosa sphagnicola|ID-Unpublished|Norway.Troms|BOLD:AAF7515
Pardosa sphagnicola|ID-Unpublished|Finland|BOLD:AAF7515
Pardosa riparia|ID-Unpublished|Czech Republic.Plzen|BOLD:AAF7515
Pardosa prativaga|ID-Unpublished|Russia.Ulyanovskaya Oblast|BOLD:AAF7515
Pardosa riparia|ID-Unpublished|Austria.Salzburg|BOLD:AAF7515
Pardosa pullata|GENHP328-11|France.Normandie|BOLD:AAF7515
Pardosa sphagnicola|ID-Unpublished|Finland|BOLD:AAF7515
Pardosa pullata|GENHP342-11|France.Normandie|BOLD:AAF7515
Pardosa fulvipes|ID-Unpublished|Italy|BOLD:AAF7515
Pardosa fulvipes|ID-Unpublished|Italy|BOLD:AAF7515
Pardosa prativaga|SMNKA196-16|Germany.Baden-Wuerttemberg|BOLD:AAF7515
Pardosa fulvipes|ID-Unpublished|Italy|BOLD:AAF7515
Pardosa prativaga|SMNKA176-16|Germany.Baden-Wuerttemberg|BOLD:AAF7515
Pardosa prativaga|ID-Unpublished|Finland|BOLD:AAF7515
Pardosa prativaga|GBBSP442-15|Germany.Mecklenburg-Vorpommern|BOLD:AAF7515
Pardosa prativaga|GBBSP221-15|Germany.Schleswig-Holstein|BOLD:AAF7515
Pardosa pullata|GENHP111-11|France|BOLD:AAF7515
Pardosa|ID-Unpublished|Belarus.Minskaya Voblasts|BOLD:AAF7515
Pardosa|ID-Unpublished|Belarus.Minskaya Voblasts|BOLD:AAF7515
Pardosa prativaga|ID-Unpublished|Finland|BOLD:AAF7515
Pardosa pullata|GENHP110-11|France|BOLD:AAF7515
Pardosa fulvipes|ID-Unpublished|Norway|BOLD:AAF7515
Pardosa prativaga|SMNKA185-16|Germany.Baden-Wuerttemberg|BOLD:AAF7515
Pardosa prativaga|ID-Unpublished|Norway.Vestfold|BOLD:AAF7515
Pardosa prativaga|ID-Unpublished|Norway.Vestfold|BOLD:AAF7515
Pardosa prativaga|ID-Unpublished|Norway.Vestfold|BOLD:AAF7515
Pardosa prativaga|ID-Unpublished|Finland|BOLD:AAF7515
Pardosa fulvipes|ID-Unpublished|Finland.Northern Ostrobothnia|BOLD:AAF7515

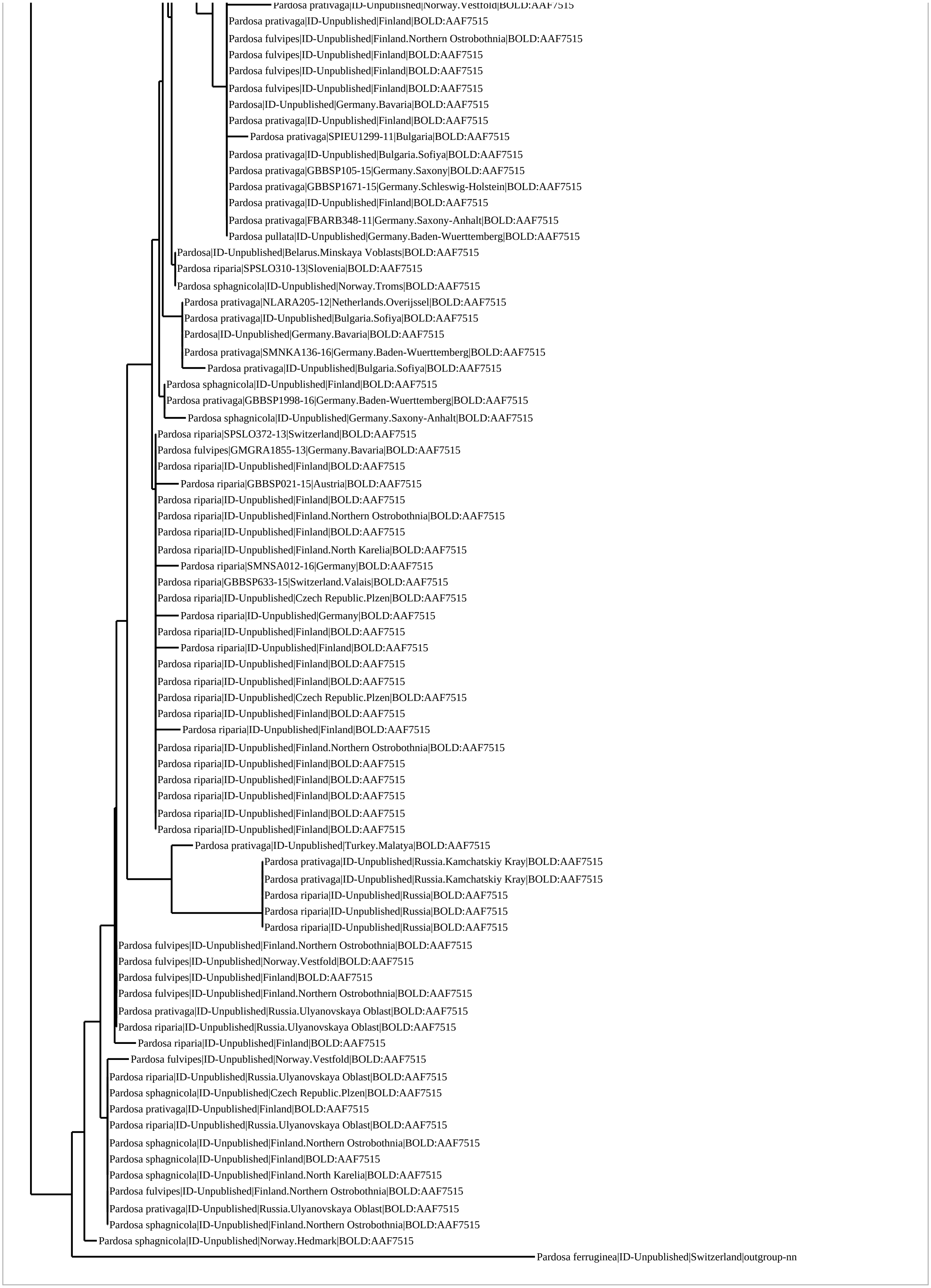
Pardosa prativaga|ID-Unpublished|Norway.Vestfold|BOLD:AAF7515
Pardosa prativaga|ID-Unpublished|Finland|BOLD:AAF7515
Pardosa fulvipes|ID-Unpublished|Finland.Northern Ostrobothnia|BOLD:AAF7515
Pardosa fulvipes|ID-Unpublished|Finland|BOLD:AAF7515
Pardosa fulvipes|ID-Unpublished|Finland|BOLD:AAF7515
Pardosa fulvipes|ID-Unpublished|Finland|BOLD:AAF7515
Pardosa|ID-Unpublished|Germany.Bavaria|BOLD:AAF7515
Pardosa prativaga|ID-Unpublished|Finland|BOLD:AAF7515
Pardosa prativaga|SPIEU1299-11|Bulgaria|BOLD:AAF7515
Pardosa prativaga|ID-Unpublished|Bulgaria.Sofiya|BOLD:AAF7515
Pardosa prativaga|GBBSP105-15|Germany.Saxony|BOLD:AAF7515
Pardosa prativaga|GBBSP1671-15|Germany.Schleswig-Holstein|BOLD:AAF7515
Pardosa prativaga|ID-Unpublished|Finland|BOLD:AAF7515
Pardosa prativaga|FBARB348-11|Germany.Saxony-Anhalt|BOLD:AAF7515
Pardosa pullata|ID-Unpublished|Germany.Baden-Wuerttemberg|BOLD:AAF7515
Pardosa|ID-Unpublished|Belarus.Minskaya Voblasts|BOLD:AAF7515
Pardosa riparia|SPSLO310-13|Slovenia|BOLD:AAF7515
Pardosa sphagnicola|ID-Unpublished|Norway.Troms|BOLD:AAF7515
Pardosa prativaga|NLARA205-12|Netherlands.Overijssel|BOLD:AAF7515
Pardosa prativaga|ID-Unpublished|Bulgaria.Sofiya|BOLD:AAF7515
Pardosa|ID-Unpublished|Germany.Bavaria|BOLD:AAF7515
Pardosa prativaga|SMNKA136-16|Germany.Baden-Wuerttemberg|BOLD:AAF7515
Pardosa prativaga|ID-Unpublished|Bulgaria.Sofiya|BOLD:AAF7515
Pardosa sphagnicola|ID-Unpublished|Finland|BOLD:AAF7515
Pardosa prativaga|GBBSP1998-16|Germany.Baden-Wuerttemberg|BOLD:AAF7515
Pardosa sphagnicola|ID-Unpublished|Germany.Saxony-Anhalt|BOLD:AAF7515
Pardosa riparia|SPSLO372-13|Switzerland|BOLD:AAF7515
Pardosa fulvipes|GMGRA1855-13|Germany.Bavaria|BOLD:AAF7515
Pardosa riparia|ID-Unpublished|Finland|BOLD:AAF7515
Pardosa riparia|GBBSP021-15|Austria|BOLD:AAF7515
Pardosa riparia|ID-Unpublished|Finland|BOLD:AAF7515
Pardosa riparia|ID-Unpublished|Finland.Northern Ostrobothnia|BOLD:AAF7515
Pardosa riparia|ID-Unpublished|Finland|BOLD:AAF7515
Pardosa riparia|ID-Unpublished|Finland.North Karelia|BOLD:AAF7515
Pardosa riparia|SMNSA012-16|Germany|BOLD:AAF7515
Pardosa riparia|GBBSP633-15|Switzerland.Valais|BOLD:AAF7515
Pardosa riparia|ID-Unpublished|Czech Republic.Plzen|BOLD:AAF7515
Pardosa riparia|ID-Unpublished|Germany|BOLD:AAF7515
Pardosa riparia|ID-Unpublished|Finland|BOLD:AAF7515
Pardosa riparia|ID-Unpublished|Finland|BOLD:AAF7515
Pardosa riparia|ID-Unpublished|Finland|BOLD:AAF7515
Pardosa riparia|ID-Unpublished|Finland|BOLD:AAF7515
Pardosa riparia|ID-Unpublished|Czech Republic.Plzen|BOLD:AAF7515
Pardosa riparia|ID-Unpublished|Finland|BOLD:AAF7515
Pardosa riparia|ID-Unpublished|Finland|BOLD:AAF7515
Pardosa riparia|ID-Unpublished|Finland.Northern Ostrobothnia|BOLD:AAF7515
Pardosa riparia|ID-Unpublished|Finland|BOLD:AAF7515
Pardosa riparia|ID-Unpublished|Finland|BOLD:AAF7515
Pardosa riparia|ID-Unpublished|Finland|BOLD:AAF7515
Pardosa riparia|ID-Unpublished|Finland|BOLD:AAF7515
Pardosa riparia|ID-Unpublished|Finland|BOLD:AAF7515
Pardosa prativaga|ID-Unpublished|Turkey.Malatya|BOLD:AAF7515
Pardosa prativaga|ID-Unpublished|Russia.Kamchatskiy Kray|BOLD:AAF7515
Pardosa prativaga|ID-Unpublished|Russia.Kamchatskiy Kray|BOLD:AAF7515
Pardosa riparia|ID-Unpublished|Russia|BOLD:AAF7515
Pardosa riparia|ID-Unpublished|Russia|BOLD:AAF7515
Pardosa riparia|ID-Unpublished|Russia|BOLD:AAF7515
Pardosa fulvipes|ID-Unpublished|Finland.Northern Ostrobothnia|BOLD:AAF7515
Pardosa fulvipes|ID-Unpublished|Norway.Vestfold|BOLD:AAF7515
Pardosa fulvipes|ID-Unpublished|Finland|BOLD:AAF7515
Pardosa fulvipes|ID-Unpublished|Finland.Northern Ostrobothnia|BOLD:AAF7515
Pardosa prativaga|ID-Unpublished|Russia.Ulyanovskaya Oblast|BOLD:AAF7515
Pardosa riparia|ID-Unpublished|Russia.Ulyanovskaya Oblast|BOLD:AAF7515
Pardosa riparia|ID-Unpublished|Finland|BOLD:AAF7515
Pardosa fulvipes|ID-Unpublished|Norway.Vestfold|BOLD:AAF7515
Pardosa riparia|ID-Unpublished|Russia.Ulyanovskaya Oblast|BOLD:AAF7515
Pardosa sphagnicola|ID-Unpublished|Czech Republic.Plzen|BOLD:AAF7515
Pardosa prativaga|ID-Unpublished|Finland|BOLD:AAF7515
Pardosa riparia|ID-Unpublished|Russia.Ulyanovskaya Oblast|BOLD:AAF7515
Pardosa sphagnicola|ID-Unpublished|Finland.Northern Ostrobothnia|BOLD:AAF7515
Pardosa sphagnicola|ID-Unpublished|Finland|BOLD:AAF7515
Pardosa sphagnicola|ID-Unpublished|Finland.North Karelia|BOLD:AAF7515
Pardosa fulvipes|ID-Unpublished|Finland.Northern Ostrobothnia|BOLD:AAF7515
Pardosa prativaga|ID-Unpublished|Russia.Ulyanovskaya Oblast|BOLD:AAF7515
Pardosa sphagnicola|ID-Unpublished|Finland.Northern Ostrobothnia|BOLD:AAF7515
Pardosa sphagnicola|ID-Unpublished|Norway.Hedmark|BOLD:AAF7515
Pardosa ferruginea|ID-Unpublished|Switzerland|outgroup-nn
